# Addressing barriers and identifying facilitators to support informed consent and recruitment in the Cavernous malformations A Randomised Effectiveness (CARE) pilot phase trial: insights from the integrated QuinteT recruitment intervention (QRI)

**DOI:** 10.1016/j.eclinm.2024.102557

**Published:** 2024-04-18

**Authors:** Julia Wade, Nicola Farrar, Alba X. Realpe, Jenny L. Donovan, Laura Forsyth, Kirsty A. Harkness, Peter J.A. Hutchinson, Neil Kitchen, Steff C. Lewis, James J.M. Loan, Jacqueline Stephen, Rustam Al-Shahi Salman, CARE pilot trial collaboration, Conor Mallucci, Philip M. White, Madeleine Eriksson, Raza Hayat, Elaine Kinsella, Katherine Lewis, Aileen R. Neilson, David C.S. White, Julia Boyd, Alastair Bullen, Morag Maclean, Andrew Stoddart, Sandra Phair, Helen Evans, Jo Noakes, Debra Alexander, Catriona Keerie, Christopher Linsley, Garry Milne, John Norrie, Janet Bunch, Kathryn Douthwaite, Simon Temple, James Hogg, David Scott, Pat Spallone, Ian Stuart, Joanna M. Wardlaw, Jeb Palmer, Eleni Sakka, Nitin Mukerji, Emanuel Cirstea, Susan Davies, Venetia Giannakaki, Ammar Kadhim, Oliver Kennion, Moidul Islam, Lucie Ferguson, Manjunath Prasad, Andrew Bacon, Emma Richards, Jo Howe, Christine Kamara, Jonathan Gardner, Madalina Roman, Mary Sikaonga, Julian Cahill, Alex Rossdeutsch, Varduhi Cahill, Imron Hamina, Kishor Chaudhari, Mihai Danciut, Emma Clarkson, Anna Bjornson, Diederik Bulters, Ronneil Digpal, Winnington Ruiz, Mirriam Taylor, Divina Anyog, Katarzyna Tluchowska, Jackson Nolasco, Daniel Brooks, Kleopatra Angelopoulou, Bethany Welch, Nicole Broomes, Ioannis Fouyas, Allan MacRaild, Chandru Kaliaperumal, Jessica Teasdale, Michelle Coakley, Paul Brennan, Drahoslav Sokol, Anthony Wiggins, Mairi MacDonald, Sarah Risbridger, Pragnesh Bhatt, Janice Irvine, Sohail Majeed, Sandra Williams, John Reid, Annika Walch, Farah Muir, Janneke van Beijnum, Paul Leach, Tom Hughes, Milan Makwana, Khalid Hamandi, Dympna McAleer, Belinda Gunning, Daniel Walsh, Oliver Wroe Wright, Sabina Patel, Nihal Gurusinghe, Saba Raza-Knight, Terri-Louise Cromie, Allan Brown, Sonia Raj, Ruth Pennington, Charlene Campbell, Shakeelah Patel, Francesca Colombo, Mario Teo, Jack Wildman, Kerry Smith, Elizabeth Goff, Deanna Stephens, Borislava Borislavova, Ruth Worner, Sandeep Buddha, Philip Clatworthy, Richard Edwards, Evangeline Clayton, Karen Coy, Lisa Tucker, Sandra Dymond, Andrew Mallick, Rebecca Hodnett, Francesca Spickett-Jones, Patrick Grover, Azra Banaras, Sifelani Tshuma, William Muirhead, Ciaran Scott Hill, Rupal Shah, Thomas Doke, Rebecca Hall, Sonny Coskuner, Laura Aslett, Raghu Vindlacheruvu, Anthony Ghosh, Teresa Fitzpatrick, Lauren Harris, Tom Hayton, Arlo Whitehouse, Andrew McDarby, Rebecca Hancox, Claudia Kate Auyeung, Ramesh Nair, Rhys Thomas, Heather McLachlan, Athanasia Kountourgioti, Guillelme Orjales, Jan Kruczynski, Sophie Hunter, Niamh Bohnacker, Rosette Marimon, Lydia Parker, Oishik Raha, Puneet Sharma, Christopher Uff, Geetha Boyapati, Marios Papadopoulos, Siobhan Kearney, Ravindran Visagan, Ellaine Bosetta, Hasan Asif, Adel Helmy, Liliana Chapas, Silvia Tarantino, Karen Caldwell, Mathew Guilfoyle, Smriti Agarwal, Daniel Brown, Sarah Holland, Tamara Tajsic, Clare Fletcher, Aisha Sebyatki, Shungu Ushewokunze, Sarah Ali, John Preston, Carole Chambers, Mohammed Patel, Daniel Holsgrove, Danielle McLaughlan, Tracey Marsden, Francesca Colombo, Kathryn Cawley, Hellen Raffalli, Stephanie Lee, Anil Israni, Rachael Dore, Taya Anderson, Dawn Hennigan, Shelley Mayor, Samantha Glover, Emmanuel Chavredakis, Debbie Brown, Giannis Sokratous, John Williamson, Cathy Stoneley, Andrew Brodbelt, Jibril Osman Farah, Sarah Illingworth, Anastasios Benjamin Konteas, Deborah Davies, Carol Owen, Loretta Kerr

**Affiliations:** a Bristol Medical School, University of Bristol, 39 Whatley Road, Bristol, BS8 2PS, UK; b Edinburgh Clinical Trials Unit, Usher Institute, University of Edinburgh, NINE Edinburgh BioQuarter, 9 Little France Road, EH16 4UK, UK; c Department of Neurology, The Royal Hallamshire Hospital, Glossop Road, Sheffield, S10 2JF, UK; d Department of Clinical Neurosciences, University of Cambridge, Cambridge Biomedical Campus, CB3 0QQ, UK; e Department of Neurosurgery, National Hospital for Neurology and Neurosurgery, University College London Hospitals NHS Foundation Trust, Queen's Square, London, WC1N 3BG, UK; f Centre for Clinical Brain Sciences, University of Edinburgh, 49 Little France Crescent, Edinburgh, EH16 4SB, UK

**Keywords:** Adult, Child, Humans, Hemangioma, Cavernous, Central nervous system, Neurosurgery, Radiosurgery, Feasibility studies, Pilot projects, Qualitative research, Randomised controlled trials as topic

## Abstract

**Background:**

It was anticipated that recruitment to the Cavernous malformations: A Randomised Effectiveness (CARE) pilot randomised trial would be challenging. The trial compared medical management and surgery (neurosurgical resection or stereotactic radiosurgery) with medical management alone, for people with symptomatic cerebral cavernous malformation (ISRCTN41647111). Previous trials comparing surgical and medical management for intracranial vascular malformations failed to recruit to target. A QuinteT Recruitment Intervention was integrated during trial accrual, September 2021–April 2023 inclusive, to improve informed consent and recruitment.

**Methods:**

The QuinteT Recruitment Intervention combined iterative collection and analysis of quantitative data (28 trial site screening logs recording numbers/proportions screened, eligible, approached and randomised) and qualitative data (79 audio-recorded recruitment discussions, 19 interviews with healthcare professionals, 11 interviews with patients, 2 investigator workshops, and observations of study meetings, all subject to thematic, content or conversation analysis). We triangulated quantitative and qualitative data to identify barriers and facilitators to recruitment and how and why these arose. Working with the chief investigators and trial management group, we addressed barriers and facilitators with corresponding actions to improve informed consent and recruitment.

**Findings:**

Barriers identified included how usual care practices made equipoise challenging, multi-disciplinary teams sometimes overrode recruiter equipoise and logistical issues rendered symptomatic cavernoma diagnosis and assessment for stereotactic radiosurgery challenging. Facilitators identified included the preparedness of some neurosurgeons’ to offer surgery to people otherwise offered medical management alone, multi-disciplinary team equipoise, and effective information provision presenting participation as a solution to equipoise regarding management. Actions, before and during recruitment, to improve inclusivity of site screening, approach and effectiveness of information provision resulted in 72 participants recruited following a 5-month extension, exceeding the target of 60 participants.

**Interpretation:**

QuinteT Recruitment Intervention insights revealed barriers and facilitators, enabling identification of remedial actions. Recruitment to a definitive trial would benefit from further training/support to encourage clinicians to be comfortable approaching patients to whom medical management is usually offered, and broadening the pool of neurosurgeons and multi-disciplinary team members prepared to offer surgery, particularly stereotactic radiosurgery.

**Funding:**

National Institute for Health and Care Research.

**Research in context**

**Evidence before this study**

Randomised controlled trials comparing surgical and medical interventions are challenging to deliver for a number of reasons, particularly in relation to equipoise. The leading research priority for people with symptomatic cerebral cavernous malformation is whether medical management and surgery (neurosurgical resection or stereotactic radiosurgery) or medical management alone is superior for improving outcome. Randomised controlled trials comparing surgical and medical interventions for other types of intracranial vascular malformation have not recruited to target.

**Added value of this study**

The QuinteT Recruitment Intervention is a useful and important adjunct to understanding the barriers and addressing the facilitators to recruitment to trials comparing surgical and medical interventions. The Cavernous malformations: A Randomised Effectiveness (CARE) pilot trial was the first randomised controlled trial to address the therapeutic dilemma for symptomatic cerebral cavernous malformation, and to embed a QuinteT Recruitment Intervention to understand recruitment barriers and facilitators and develop corresponding actions to optimise informed consent and recruitment. The main barriers related to equipoise and patient eligibility, with conventions of usual care making a position of equipoise challenging, lack of multi-disciplinary teams equipoise preventing the approach of some patients with symptomatic brain cavernoma, and logistical issues surrounding diagnosis and assessment for stereotactic radiosurgery adding further barriers. The main facilitators included the high level of commitment of the chief investigators and key principal investigators prepared to change practice within the research context to offer surgery to people who would have been offered medical management alone outside the study. Multi-disciplinary team equipoise and skilful information provision to facilitate patient understanding that the trial could be viewed as a solution to uncertainty regarding management were key in optimising recruitment.

**Implications of all the available evidence**

Challenges with clinical equipoise and eligibility are common in trials comparing surgical techniques and medical management, particularly where routine care defaults to one of the interventions compared. An integrated QuinteT Recruitment Intervention can identify barriers and facilitators specific to the trial with tailored actions to optimise informed consent and achieve target recruitment. Recruitment to a definitive trial would benefit from training and support to encourage more clinicians to be comfortable approaching patients for whom medical management alone would conventionally be offered, and broaden the pool of surgeons and multi-disciplinary team members prepared to offer surgical interventions, particularly stereotactic radiosurgery, within a trial context.

## Introduction

Cerebral cavernous malformations cause intracranial haemorrhage, focal neurological deficit or epileptic seizure, and affect around 106,000 in the UK,^1^ with an annual incidence of ∼160 people.^2^ Treatment options include medical management, neurosurgical excision and stereotactic radiosurgery. Observational studies comparing treatments for cerebral cavernous malformations exist^2^, ^3^, ^4^, ^5^ but no randomised controlled trials. A recent James Lind Alliance priority-setting exercise identified the top research priority for cerebral cavernous malformation as ‘Does treatment (neurosurgery or stereotactic radiosurgery) or no treatment improve outcome for people with symptomatic cerebral cavernous malformation’? The Cavernous malformations: A Randomised Effectiveness (CARE) pilot trial (ISRCTN41647111) aimed to determine feasibility of a trial comparing medical management and surgery (neurosurgical resection or stereotactic radiosurgery) with medical management alone for improving outcome for people with symptomatic cerebral cavernous malformation.^6^

We anticipated recruitment to this pilot trial would be challenging: similar trials closed early following recruitment challenges^7^ or showing short term benefit for medical management alone.^8^ It was anticipated there would be concerns about equipoise within an open trial comparing surgery with medical management, when intracranial haemorrhage/focal neurological deficit or epileptic seizures from cerebral cavernous malformation are mostly of mild-moderate severity, which in turn has led to a ‘watch and wait’ approach being commonplace in the UK and Ireland. Concerns about use of stereotactic radiosurgery for some potential participants, the low incidence of cerebral cavernous malformation,^2^ variation in access to stereotactic radiosurgery in usual care^9^^,^^10^ and potential patient and/or family preferences could also negatively affect recruitment.

The QuinteT Recruitment Intervention (QRI),^11^ integrated within the CARE pilot phase trial,^6^ aimed to understand barriers and facilitators and develop corresponding actions optimising informed consent and recruitment.

## Methods

The protocol for the CARE pilot and feasibility trial is described in detail elsewhere.^6^ Potential participants or families/carers were approached by neurosurgeons, neurologists or at joint trial neurosurgeon/neurologist/specialist nurse clinics. Patient support organisations (Cavernoma Alliance UK [https://cavernoma.org.uk/] and Cavernoma Ireland [https://www.facebook.com/CavernomaIrelandSupport/]) promoted the study from May 2021 onwards^12^ and throughout recruitment (a 20-month period from September 2021 to April 2023 inclusive) which incorporated a 5-month extension onto the originally projected 18-month recruitment period, June 2021–November 2022.^13^

### Study design and data collection

The CARE-QRI was a complex, adaptive, mixed methods intervention to improve information provision and recruitment, and the QRI methodology is described in detail elsewhere.^11^^,^^14^ The QRI methodology is a pragmatic intervention adapted to each host trial^14^ and uses data collection and analysis in line with what is most appropriate for the research objective.^15^ The CARE-QRI was adapted to incorporate pre-accrual data collection and recruiter training. Pre-accrual we investigated barriers and facilitators during healthcare professional workshops, then worked with the participant advisory group to co-design participant information. Site-initiation involved pre-recorded online training on the trial protocol and optimising communication with potential participants, followed by live online training to discuss recruitment pathways and investigate site-team equipoise using case discussions, delivered in collaboration with the chief investigators. During recruitment, we collected site and trial-wide screening log data, applying the QRI-Screened, Eligible, Approached, Randomised (SEAR) framework^16^ to identify where on the recruitment pathway into the trial and at which sites patients were ‘lost’ to recruitment, with reasons why. Patterns identified from screening log data, as to where and why substantial proportions of patients were leaving the pathway into the study at individual sites, guided the sampling of audio-recorded recruitment discussions and healthcare professional and patient interviews. In turn, findings from qualitative data collection informed further scrutiny of screening log data, in an iterative process to confirm or contradict findings.^14^ The aim was to look for convergence and divergence across all data sources, with qualitative data used to expand and explain patterns found in the quantitative screening log data.

Qualitative data sampling was purposive: we aimed for as diverse a sample as possible in terms of high vs low recruiting sites, whilst investigating patterns identified in the screening log data. We aimed for a sample of patients declining participation or withdrawing from the trial, across a range of sites and for whom a paired audio-recorded recruitment discussion was available. We observed all trial management group/principal investigator meetings and trial correspondence for recruitment-related issues. Qualitative data collection investigated all evidence regarding site-, recruiter-, and patient-level patterns and decision-making processes to investigate variations and systematic patterning in: i) how eligibility criteria were applied; ii) proportions of eligible patients approached (by individual recruiters and across sites); and iii) proportions of approached patients who consented to participate. Table 1 shows the CARE-QRI components: screening log and qualitative data were triangulated to identify recruitment barriers and facilitators and corresponding actions, in collaboration with the chief investigators and trial management group.

**Table 1:** Components of the QuinteT recruitment intervention as applied within the CARE pilot trial.

Data collection^a^	Issues identified
**Pre accrual**	
**1. Health care professional workshops** **2. Site initiation visits**	Small patient pool highlighted importance of including all people with symptomatic brain cavernoma in screening. Usual care practice (monitor until intervention required) rendered equipoise challenging. Shifting usual care practice towards earlier neurosurgical intervention for those presenting with seizures. Variations in application of eligibility criteria and recruiter perception of equipoise via case discussion. Concerns about offering SRS.
**During accrual**	
**3. Screening log data**	Cross site variation (see Table 2) indicated that at some sites there was: - Exclusion of some people with symptomatic brain cavernoma from entry on screening logs; - Exclusion of some people with symptomatic brain cavernoma from approach; - Variation in proportion of those approached accepting participation.
**4. Recruitment discussions** (audio recorded)	Recruiter difficulties with: - Conveying equipoise, in particular to those for whom medical management has previously been recommended, those presenting with seizures, those referred for stereotactic radiosurgery (SRS) prior to trial start. - Addressing patient preferences; - Establishing patient equipoise prior to randomisation; - Conveying reason for randomisation. Some patients reluctant for intervention if no longer experiencing symptoms and generally patient preferences favoured less invasive intervention (SRS over neurosurgery, medical management over surgical intervention); Some patients found randomisation within CARE acceptable >12 months post initial presentation of symptoms (note this was counter to recruiter expectations) with some perceiving CARE trial to be offering surgical intervention not otherwise offered outside the trial; Parental equipoise for randomisation between neurosurgery and medical management for small number (N = 3 audio recordings) of paediatric patients.
**5. Interviews** **1) Healthcare professionals** **2) Patients declining or withdrawing from participation**	Recruiter controversy: regarding SRS for paediatric participants; regarding SRS for people presenting with symptomatic cavernoma generally and for people presenting with seizures in particular; regarding comparison of surgical interventions vs medical management over the limited timespan of a clinical trial. Some recruiters prepared to offer randomisation to people to whom medical management offered outside the trial. Patient misunderstanding of information provision: monitoring followed by intervention. Patient preferences generally mirrored what they understood to have been recommended by clinicians and generally favoured non-invasive intervention (medical management or SRS) but small number saw trial as means to access surgical intervention not perceived as offered outside of the trial.
**6. Observation of trial management group, CARE club discussions, Investigator and research practitioner/coordinator/nurse meetings, trial emails.**	Recruiter concerns at approaching prevalent patients, previously recommended medical management. Controversy regarding SRS for paediatric participants, for symptomatic cavernoma generally, for symptomatic cavernoma presenting with seizures specifically. Uncertainty regarding process of referral for SRS within trial. Withdrawal of participants immediately following randomisation, indicating lack of participant equipoise at point of randomisation.
**Actions, training, feedback**^b^	**Issues addressed**

**Pre accrual**	
**7. Co-design of participant facing information**	Presentation of equipoise using table comparing processes, benefits and risks of each intervention. Terminology used: ‘treatment including surgery’ vs ‘treatment without surgery’, ‘study’ in place of ‘trial’.
**8. QRI site initiation training in information provision**	Framing study (UK wide, government funded, in response to uncertainty in evidence base), terminology (as above), presentation of equipoise – 339 views.
**9. Co-delivery of site initiation visits**	Discussion of site multi disciplinary team and screening processes to maximise N of patients screened for trial. Discussion of case studies to optimise screening for eligibility and encourage equipoise.
**During accrual**	
**10. Tips and guidance documents** v1 (October 2021) v2 (March 2022) v3 (December 2022)	Framing study (as above), terminology (as above), equipoise (as above) As above, plus addressing patient preferences, explaining reason for randomisation, presenting the surgical intervention arm first (most commonly the non-preferred arm), presenting benefits prior to risks, optimising site screening processes, use of terminology.
**11. CARE CI chats**: Pre-recorded online discussions involving chief investigators, trial management groups, QRI team exploring relevant issues via 5–10 min conversation (January–May 2022)	Do I really have to do these audio recordings and will they help?—47 views. How to complete screening logs—59 views Screen-as-you-go—27 views Logistics of recruitment—33 views When to randomise?—55 views Approaching patients diagnosed long ago & treated without surgery – 15 views Tips for conversations about the CARE study—26 views Latest top tips for recruitment conversations—6 views Audio Recordings—20 views How to describe randomisation—15 views SRS process for referral—35 views
**12. 3x online Investigator and 1x online research nurse/coordinator meetings** May–September 2021	Optimising site screening/approach processes, addressing patient preferences, explaining reason for randomisation, presenting the surgical intervention arm first (commonly non-preferred), presenting benefits prior to risks.
**13. CARE clubs** December–March 2021–22 Live 30 min online discussion meetings to which all principal investigators invited.	Addressing questions about trial/QRI processes.
**14. Individual recruiter feedback** (doubling as interviews where possible)	Individual feedback to optimise recruitment discussions, including terminology, order of presentation, dealing with participant preferences, establishing participant equipoise prior to randomisation taking place.

a Some QRI activity simultaneously combined data collection and training/feedback.

b Weekly CARE Newsflashes and monthly CARE newsletters (emailed to sites teams) reinforced key QRI messages in addition to all above.

### Data analysis and actions to support recruitment

**Fig. 1:**
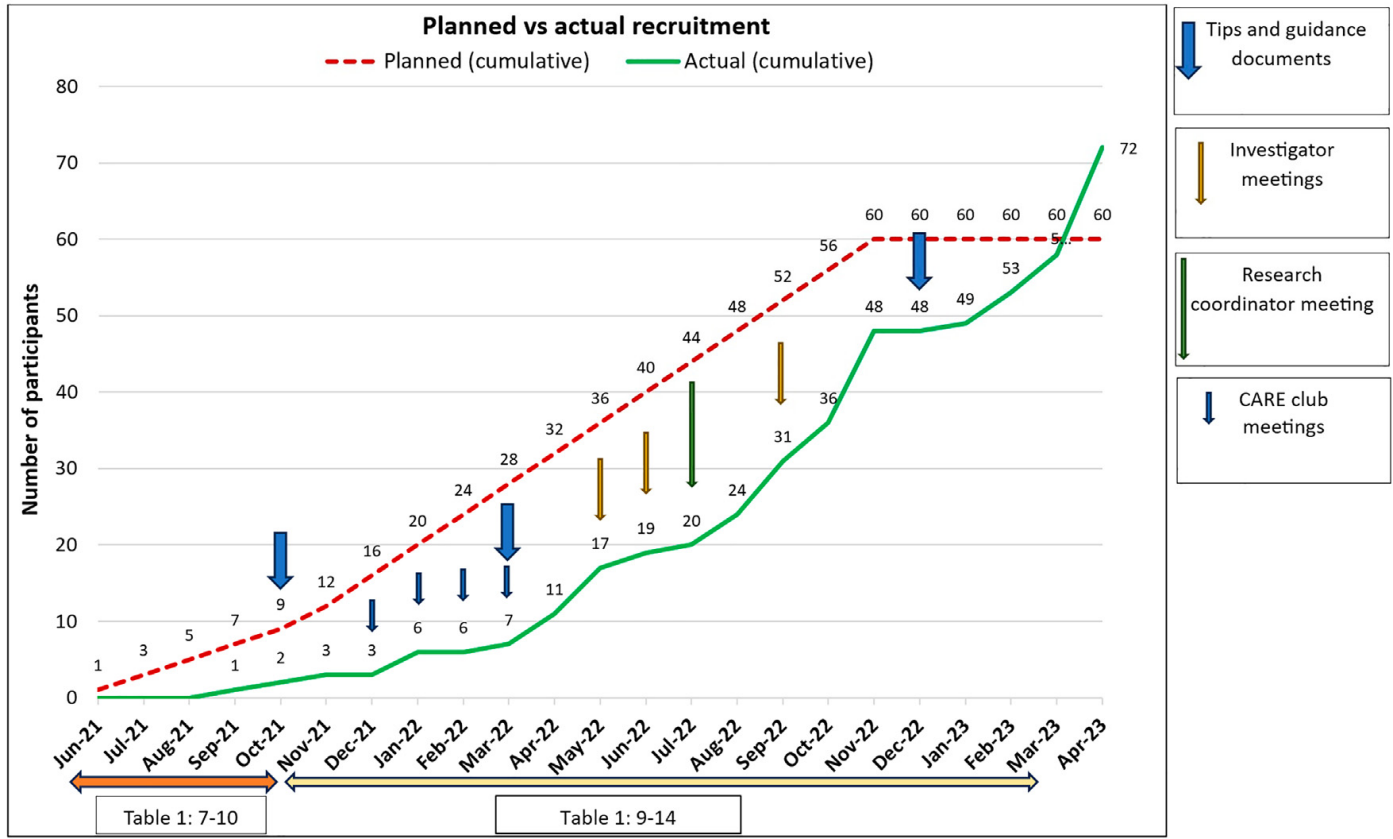
**QRI interventions shown against recruitment**.

QRI screening data^16^ analysis reported descriptive statistics: numbers and percentages of patients screened, eligible, approached and randomised at individual sites and overall (Table 2). Descriptive statistics were used^13^ as the interpretation of the raw data enabled detection of differences between sites in reporting and screening practice, and provided insight into variations in approaches to screening and recruiting patients. Sites where numbers or percentages were substantially higher or lower than the mean across all sites were noted for scrutiny to determine whether and what further investigation using qualitative data collection was warranted. We analysed workshop, site initiation visit, recruitment discussion, interview and observational data (Table 1) using techniques of thematic,^17^ content^18^ and conversation analysis^19^ and in line with reporting criteria for qualitative studies^20^ [Appendix pp 1–5] to identify recruitment barriers and facilitators, impacting on individual sites or trial wide. We discussed findings with the chief investigators, principal investigators and trial management group and collaboratively developed trial-wide and individual site/recruiter training, actions and feedback to improve informed consent and recruitment (Table 1, Fig. 1). Data collection, analysis and implementation of training and actions took place iteratively, pre-accrual then throughout recruitment (Table 1).^11^^,^^14^ Full analysis of the entire dataset was completed after recruitment ended.

**Table 2:** Numbers and percentages screened, eligible, approached and randomised,^16^ shown by site across 28 sites activated and totals.

Site ID	N screened	Eligible (of those screened)^b^	Approached (of those eligible)^c^	Doctor/patient uncertain (of those approached)	Consented (of those doctor & patient uncertain)	Randomised (of those eligible)	Randomised (of those approached)^d^
11	37	30 (81%)	18 (60%)	5 (28%)	4 (80%)	4 (13%)	4 (22%)
13	4	2 (50%)	2 (100%)	1 (50%)	1 (100%)	1 (50%)	1 (50%)
14 (P)	7	5 (71%)	2 (40%)	0 (0%)	0 (0.0%)	0 (0%)	0 (0%)
15	37	28 (76%)	24 (86%)	6 (25%)	2 (33%)	2 (7%)	2 (8%)
16	49	25 (51%)	17 (68%)	16 (94%)	12 (75%)	12 (48%)	12 (71%)
17	18	17 (94%)	11 (65%)	2 (18%)	1 (50%)	1 (6%)	1 (9%)
18 (P)	10	4 (40%)	2 (50%)	1 (50%)	1 (100%)	1 (25%)	1 (50%)
19	14	9 (64%)	7 (78%)	5 (71%)	5 (100%)	4 (44%)	4 (57%)
22^a^	1	1 (100%)	1 (100%)	1 (100%)	1 (100%)	1 (100%)	1 (100%)
23 (P)	7	3 (43%)	1 (33%)	1 (100%)	0 (0%)	0 (0%)	0 (0%)
25	3	3 (100%)	3 (100%)	2 (67%)	2 (100%)	2 (67%)	2 (67%)
26 (P)	2	2 (100%)	2 (100%)	2 (100%)	2 (100%)	2 (100%)	2 (100%)
27	11	9 (82%)	8 (89%)	3 (38%)	3 (100%)	3 (33%)	3 (38%)
30	21	20 (95%)	20 (100%)	10 (50%)	8 (80%)	8 (40%)	8 (40%)
31^a^	0	0 (0%)	0 (0%)	0 (0%)	0 (0.0%)	0 (0.0%)	0 (0%)
33	1	1 (100%)	1 (100%)	1 (100%)	1 (100%)	1 (100%)	1 (100%)
34	72	24 (33%)	7 (29%)	1 (14%)	1 (100%)	1 (4%)	1 (14%)
35	68	48 (71%)	16 (33%)	6 (38%)	3 (50%)	3 (6%)	3 (19%)
36^a^	1	1 (100%)	1 (100%)	1 (100%)	1 (100%)	1 (100%)	1 (100%)
37	7	3 (43%)	1 (33%)	1 (100%)	1 (100%)	1 (33%)	1 (100%)
38 (P)	1	1 (100%)	1 (100%)	1 (100%)	0 (0%)	0 (0%)	0 (0%)
39	36	25 (69%)	24 (96%)	15 (63%)	13 (87%)	13 (52%)	13 (54%)
40	0	0 (0%)	0 (0%)	0 (0%)	0 (0.0%)	0 (0.0%)	0 (0%)
44	35	28 (80%)	5 (18%)	2 (40%)	2 (100%)	2 (7%)	2 (40%)
45	38	7 (18%)	2 (29%)	1 (50%)	1 (100%)	1 (14%)	1 (50%)
46 (P)^a^	3	2 (67%)	2 (100%)	1 (50%)	1 (100%)	1 (50%)	1 (50%)
47	27	23 (85%)	23 (100%)	11 (48%)	7 (64%)	7 (30%)	7 (30%)
48	1	1 (100%)	1 (100%)	0 (0%)	0 (0.0%)	0 (0%)	0 (0%)
**ALL SITES**	**511**	**322 (63%)**	**202 (63%)**	**96 (48%)**	**73 (76%)**	**72 (22%)**	**72 (36%)**

(P), paediatric only site where screened numbers were likely to be lower.

a Site activated during final 6 mnths of recruitment.

b Low proportions indicated highly inclusive approach to screening or problematic exclusion of potentially eligible patients.

c Low proportions indicated decision not to approach people with symptomatic brain cavernoma.

d Low proportions indicated potential difficulties discussing the trial. All above needed further investigation via qualitative data collection.

### Regulatory approvals and consent processes

Ethical approval for QRI activity was granted by the University of Bristol Faculty of Health Sciences Research Ethics Committee (REC) pre-accrual (reference 111186). The Leeds East Research Ethics Committee approved the CARE trial protocol, which included the integrated QRI (version 2.0, Mar 22, 2021). The trial co-sponsors were the University of Edinburgh and NHS Lothian Health Board. Written patient consent to participate in QRI data collection was taken independently of consent to participate in the CARE pilot trial and enabled consent to audio-recording of recruitment discussions, interviews or both. Healthcare professionals provided written consent to audio-recording of recruitment discussions and spoken consent (captured in audio recordings and recorded in writing by the researcher taking consent) to audio-recording of professional workshops and interviews.

### Role of funding

The funder of this study had no role in study design, data collection, data analysis, data interpretation, writing of the report or decision to submit.

## Results

Participants in the CARE-QRI are shown in Table 3. Triangulation of QRI screening log (Table 2) and qualitative data (Table 1, Table 3 and 4) revealed barriers and facilitators to improve informed consent and recruitment, specific to this pilot phase trial. These are presented below, with corresponding QRI actions developed and implemented in collaboration with the chief investigators, principal investigators and trial management group (Table 1, Fig. 1). Findings of the CARE pilot trial are reported in a paired publication.^13^

**Table 3:** Characteristics of participants included in qualitative data analysis.

**Neurologist workshop (pre accrual)**	**Total = 19**
N CARE sites represented	15
N Trial management group members	3
N specialist paediatric neurologists	7
**Neurosurgeon workshop (pre accrual)**	**Total = 16**
N CARE sites represented	15
N Trial management group members	3
N specialist paediatric neurosurgeons	3
**Recruitment discussions (during accrual)**	**Total = 79**
N patients	71
N CARE sites represented (18 given audio recorder)	13
N consultant neurosurgeons	14
N neurosurgical registrars	4
N neurologists	2
N recordings per site, range (median)	1–20 (4)
**Professional interviews**	**Total = 19**
Pre-accrual	9
During accrual	10
N CARE sites represented	11
N consultant neurosurgeons	10
N neurosurgical registrars	2
N neurologists	3
N Trial management group members	5
**Patient interviews (during accrual)**	**Total = 11**
N CARE sites represented	5
N decliners	10
Chose medical management	6
Chose neurosurgery	2
Chose SRS	1
N withdrawals	1
Sex	
N female	6
Age	
18–30	2
31–40	2
41–50	1
51–60	4
61–70	2

**Table 4:** Qualitative data in support of findings.

1	**Usual care and equipoise** “There's a balance of risk, a pattern of morbidity … a spectrum of how likely it is that that morbidity will be realized, that has to be balanced against the perceived effect on the natural history of the disease… I think there's a large grey area where, depending on the previous experience of the surgeon…they might be prepared to operate.” [R196 IM3 p26]
2	**Challenges for equipoise in current management of refractory epilepsy** “The neurosurgeon may not see the patients early enough in order to make that decision about early intervention, the neurologist will cycle through a whole range of agents with combinations before they refer the patient with epilepsy on to neurosurgeons” [R140, SIV, p5] “In our hospital in [SITE] if we have a symptomatic cavernoma due to a seizure, I tend to refer them early to the surgical clinic rather than trialling two drugs” [Neurologist 236, Neurologist workshop, p44]
3	**Patient preferences** “So, if I look at it from that perspective…my symptoms without surgery are not strong enough for me to warrant going in to have quite invasive brain surgery. The risk of surgery, it's just not [an option].” [P0407 Int, p5, declined randomisation neurosurgery vs medical management, chose medical management]” “The majority of specialists I've spoken to say ‘Your situation is quite stable, and I don't think you need any kind of treatment at the minute’. And some of them actually, they recommend surgery. But some say ‘No the surgery could be too risky and the gamma knife is the way forward’.” [P0409, Rec cons, p5, declined trial, chose medical management] “[All clinicians] I've spoken to have said to go for [neurosurgery]. Because, if it's not meant to be there, get it out.” [P0422 Rec Cons, declined trial, chose neurosurgery]
4	**MDT influence on recruitment** “The MDT is not unified in equipoise, that's more clear to me now than it was before we opened. And I would say, the fact that patients come through the MDT provides nothing but the opportunity for me to say, ‘Could I please talk to them about the CARE study?’ But I'll quite often be told ‘no’.” [R196 Int, site screened >30 patients, only 33% of eligible patients approached] “[MDT members] wouldn't outright say ‘not suitable for consideration in CARE’, but they would often have… a preference as to how they think the patient should be managed. There's a reason they don't want to go ahead and approach the patient…. The [neurovascular] MDT is led by a particular consultant, who will have quite a say in how they want things to move forward.” [R189 Int p9, site screened >30 patients, only 18% of eligible patients approached]
5	**Exclusion of SRS** “We are not big believers in gamma knife for cavernoma. We tend to operate on the cavernomas, including the brainstem ones, provided they've got an appropriate accessible corridor or are subpial. The general view of the MDT is ‘not for gamma knife’” [R125 SIV case discussion]. “I certainly approached other consultants just going back through our screening logs, ‘Did you want to rediscuss this patient [for SRS] at the MDT?’ They've come back and said ‘Actually no…the plan was just to carry on conservative management’.” [R196 Int p6] “I think the other issue is that a lot of people have been practising a certain way for 20 years. So they really believe in their management algorithm. So detaching yourself from your algorithm, which you've had in place for 20 years, you believe in, that is hard.” [R119 Int p8] “Radiosurgery is [seen as] a competitor to the microsurgeon because the microsurgeon sees patients boarding with their feet, going for minimally invasive treatment. The patients don't come for microsurgical resection anymore.” [R215 Int p10]
6	**Cavernoma management for children** “General feeling in paediatrics… is that 95% of these, if you can do a surgical excision, then most paediatric neurosurgeons would excise them, because they're young they have got 70, 80, 90 years of life left…Children don't usually get referred for radiotherapy for brain tumours unless they are over 5, so that's where… that 5 year old cut-off's come from…we worry about the late effects of radiosurgery, whether it be single or multiple doses.” R153, Int, p5 “I mean I've referred a couple of brainstem cavernomas to [Site offering SRS]… but obviously one of the late effects of brain radiotherapy is cavernoma formation, it's quite paradoxical.” [R235 Int p10] “Unsuitable for trial as unsuitable for surgery or SRS.” Free text screening log report why 5/7 symptomatic cavernoma patients screened at paediatric site were not approached.
7	**Challenges of determining eligibility** “There are a lot of patients that I've been seeing who are referred as symptomatic cavernoma but...when I see them that's not the case. You have to see quite a lot of patients with cavernoma to identify ones who may actually be suitable for the study. It does take quite a time for the epilepsy work up, perhaps to have more invasive EEG, neuropsychology, perhaps a psychiatry assessment. All of those things are taking much longer, unfortunately, post-pandemic.” [R144 Int p3]
8	**Controversy over eligibility criteria** “If they're presenting with headaches and they've got a cavernoma, how do you know if the headache is not due to a micro-haemorrhage from cavernoma? You don't but you can see a cavernoma. Therefore, by definition it has bled.” [R205 Int p9]
9	**Exclusion of those showing FND with no evidence of haemorrhage from SRS referral** “[if] there's no evidence that it’s bled, for example… they screened them out themselves rather than just refer everything to [SRS centre]” [R189, Int, p5]
10	**Managing expectations of people (and their clinicians) referred for SRS prior to trial opening** “[with] SRS…we are working months behind, because patients would have been referred at a time pre-CARE. Obviously, I can talk to them about it, but then you're in the [SRS] clinic and they've come for [SRS] treatment. It's much, much harder for them to then reverse that decision and decide to go into a trial.” [R178, Int, p22] “So a lot of the referrals to me are referrals for radiosurgery, so they're not being referred to enrol them in a trial. I if I get referred a patient for gamma knife and then and it comes back that I randomise a patient and they end up having conservative management, then I might find [the referring neurosurgeon] says well you know patient, has a bleed, I didn't refer the patient for randomisation, I referred them for gamma knife.” [R119 Int p4 and 5]
11	**Length of follow-up** “My gut feeling, would be that to remove the cavernoma, to remove that risk of haemorrhage [brings benefit] but whether you'd have a long enough follow-up [within a CARE main phase trial] to determine that. It's almost like the ARUBA trial in that, if you look four or five years later, you're wasting your time. You're looking at the lifetime risk of haemorrhage, not do they have a haemorrhage in the next four years.” [R178 Int p17] “You will of course see all the morbidity of [neurosurgery] very, very early. You may not see some of the benefits for decades. And particularly the seizure benefits, we could argue if you're causing harm early, that. it would take a very long time to balance that out.” [R196 Int p8]
12	**Influence of COVID-19 pandemic** “I just see the level of anxiety and preparation to come in for an operation at the moment, you know, patients still have to isolate….There's a lot more to put your life on hold for, you know isolate, do pre-OPS, come in. You may be cancelled on the day. All of that's an extra level of stress and anxiety for people compared to, if you're going in for SRS, which is on a more sessional basis, isn't it?” [R189 p 6]
13	**Surgeons offering intervention to a broader pool of patients than within usual care** “I don't normally use radiosurgery. There are reasons to think it could do something, but til the evidence is out there I wasn't planning on using it. Now there is a trial trying to get that evidence–if you want to try radiosurgery I'm happy organising it through the trial…I really don't operate on very many cavernomas. The vast majority I just wait and see what happens….There is a genuine question whether surgery might change the course of things. If you're somebody who would like to consider surgery, then I believe the way to do it, is to go through the trial.” [R167 Int p8] “According to best current evidence I would proceed with [medical management]. In the context of the trial, as we don't know for sure that medical management is the best, he should go for randomisation.” [R138 SIV case discussion]
14	**PI influence over the MDT/MDT equipoise** “Because there are just the four of us [in the MDT], I run it usually.” [R138, SIVp2 site recruited >4 patients] “We don't get cavernomas into the MDT unless there is a specific reason for it. If their diagnosis is in doubt or something of that sort. But by and large, there are two of us who do vascular work in [SITE], both of us pretty much sing from the same hymn sheet….We don't discuss every cavernoma through MDT.” [R205 Int, site recruited >4 patients] “The patient is counselled on all the potential options of just taking an observation route, microsurgical resection with its risks depending location, age and suitability, co-morbidities and so the peri-operative risk will be evaluated. Then stereotactic radiosurgery, what it can offer, what we can't guarantee, what we can't prove, like for example the controversy that we will never be able to say, “You are cured,” because it will always be visible on an MRI scan. Then at the end of that decision-making process the patient usually makes an informed decision.” [R215 Int, site recruited >4 patients]
15	**Offering SRS based on evidence of lower risk** “I think SRS patients, by and large, have already had surgery ruled out, so they have a choice of do absolutely nothing or have the SRS. The dose that we use is relatively small. The risks aren't enormous. Does it work? Does it not work? We don't know, but I just think that that's far more randomisable [than neurosurgery vs medical management].” R178 Int p5 “There can be a willingness on the part of clinicians, and to an extent on the part of patients, to accept treatment that doesn't require general anaesthetic, is done in a day and is unlikely to cause a disability. But whether it's effective or not is a secondary consideration. [p10] If you cannot accept any risk, radiosurgery is probably the way to go. But I cannot guarantee that it's efficacious.” [p26] [R196 Int]
16	**Acceptability of intervention with SRS for people with cavernoma** “There can be a willingness on the part of clinicians, and to an extent on the part of patients, to accept treatment that doesn't require general anaesthetic, is done in a day and is unlikely to cause a disability. But whether it's effective or not is a secondary consideration. [p10] If you cannot accept any risk, radiosurgery is probably the way to go. But I cannot guarantee that it's efficacious.” [p26] [R196 Int] “The operation I don't want. Not sure about the radiation.” [P1305 Rec cons, p9, accepted randomisation between SRS vs MM]
17	**SRS to prevent future bleed, not for seizure control** “I would question what benefit SRS would be to seizure outcome. So, we're now moved away from the benefit to the cavernoma outcome, and we're discussing seizure outcome. Again, am I telling patients that their seizures are going to be fewer, that their burden of disease is going to be less, that they'll be able to reduce their medication, etc? The same conversations that you would have if the patient who had a clear focus of epilepsy had that surgically removed? I just don't think that it’s the same thing.” [R178 Int p16]
18	**Patients perceiving the CARE trial as an opportunity to access neurosurgery** “The general consensus was, unless you're having severe seizures every day and it's disabling you then the cavernoma wouldn't be removed. After that, it was ‘Right, it sucks but I've just got to carry on’. (p8 and 9)The fact that [the trial] gave me the opportunity to go for surgery, it was such a huge relief. Without the CARE trial, I don't think that would have happened” [P1407 Int, p26, 7 year history of seizures, declined trial, chose neurosurgery]

R, Recruiter; IM, Investigator Meeting; SIV, Site initiation visit; P, participant; Int, interview; Rec cons, recruitment consultation.

### Barriers and actions to address these

#### Usual care practice

**Fig. 2:**
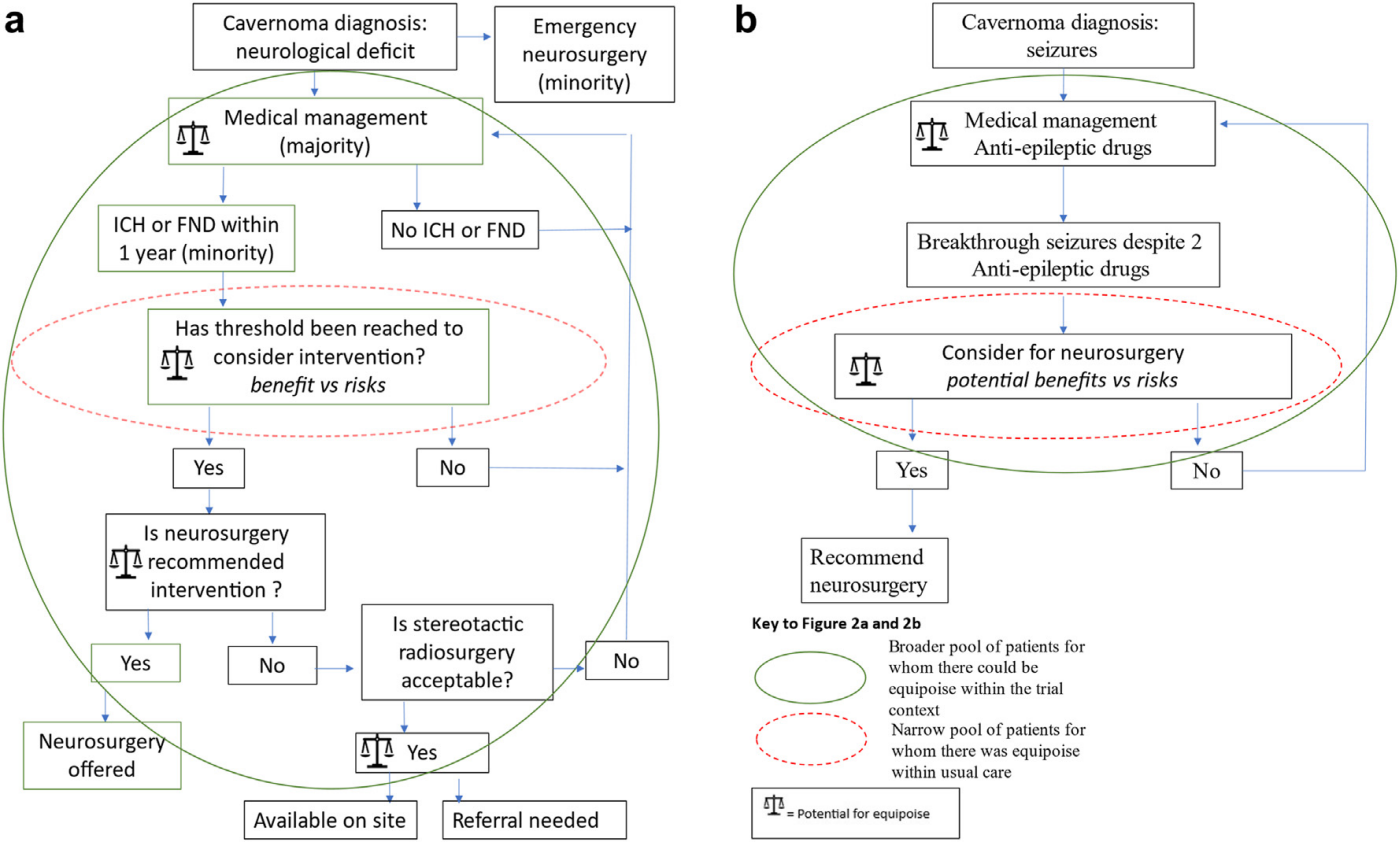
**a. Potential points of equipoise mapped against usual care pathway for cavernoma presenting with intracranial haemorrhage (ICH) or focal neurological deficit (FND). b. Potential points of equipoise mapped against usual care pathway for cavernoma presenting with seizures**.

Current UK practice for most patients presenting with symptomatic cerebral cavernous malformation defaulted to medical management, until a threshold for surgical intervention was met. Adopting a position of equipoise,^21^ for more than a narrow pool of patients, required a change from routine practice (Fig. 2a and b). The threshold for surgical intervention was conventionally defined as two or more significant haemorrhages within months or refractory seizures following trial of at least two anti-epilepsy drugs and neurosurgery was the first-line intervention considered, particularly for patients with seizures. Surgeons rejected excision when cavernoma location indicated that perceived risks outweighed potential benefits, but reported a ‘grey area’ (potential position of equipoise) where risks of surgery-related morbidity balanced risks of potential haemorrhage-related morbidity. How broad this ‘grey area’ was, varied between surgeons (Fig. 2a and b, Table 4:1). Equipoise was lost if neurosurgery was rejected as too high risk and medical management or stereotactic radiosurgery preferred.

Current approaches to surgical management for refractory epilepsy also made achieving a position of equipoise challenging: care defaulted to anti-epilepsy drugs as first-line management, then to neurosurgery if seizures became refractory (Fig. 2b). Neurologists and neurosurgeons referenced emerging evidence that earlier surgical intervention may prevent refractory epilepsy, and reported discomfort offering randomisation, preferring rapid excision (Table 4:2).

We delivered multiple interventions to encourage recruiter equipoise pre- and throughout accrual (Table 1). Pre-accrual actions included co-design of participant-facing information to incorporate a table comparing processes, benefits and risks of each intervention (Appendix p 7 and 8) training in framing the trial as a solution to the dilemma regarding what management to recommend and terminology for use in discussing the trial (‘study’ preferred over ‘trial’, terms for describing the intervention arms) and conveying equipoise. Timing of randomisation following symptom presentation (relevant for all symptomatic cavernomas but particularly for seizure management) was discussed in the CARE CI Chat 5 (pre-recorded online video discussion between the chief investigators) and via individual recruiter feedback (Table 1).

#### Patient preferences

Patients generally favoured medical management, to avoid invasive neurosurgery, particularly where symptoms resolved or caused minimal functional impairment. However, rationales underpinning patient preferences revealed these to be strongly influenced by perceived clinician recommendations, within or outside the trial (Table 4:3). Clinicians underestimated their influence on patient preferences. Recruiters assumed people with prevalent brain cavernoma would be more challenging to recruit than those newly presenting, and expressed discomfort at revising previous recommendations for medical management. The importance of including prevalent patients in screening and approach was communicated from the outset during site initiation training, and reinforced in a CARE Chat 6 (Table 1). It was notable that half the CARE pilot trial recruits experienced their most recent symptomatic episode a year or more previously, had been recommended medical management, yet accepted randomisation within the trial,^13^ evidence of the value to these patients and the trial of their inclusion. Strategies for managing patient preferences (eliciting the rationale and addressing preferences with tailored information provision^18^^,^^19^) were included in Investigator meetings (May, June, September 2022) and summarised in QRI Tips and Guidance documents (Appendix pp 13–21, Table 1/Fig. 1).

#### Role of the multi-disciplinary team

Successful recruitment required alteration of usual neurovascular multi-disciplinary team decision-making where there is uncertainty about management: consensus about management needed to be replaced by consensus about equipoise. QRI-screening log and qualitative data revealed how multi-disciplinary team decision-making could result in ‘loss’ of potential participants at all points of the recruitment pathway. Cross-site comparison of QRI-screening log data (Table 2) showed wide variation in numbers and proportions of screened patients found eligible (range 7/38 [18%]−17/18 [94%], in sites reporting 10 or more patients screened) with low numbers/proportions indicating potential exclusion of eligible patients from logs. Similarly, numbers and proportions of eligible patients approached varied greatly across sites (range 5/28 [18%]−23/23 [100%] in those sites identifying five or more patients as eligible) with low proportions indicating exclusion of people with symptomatic cerebral cavernous malformation from approach. Investigation of reasons for differences revealed variations in multi-disciplinary team thresholds for including people as eligible for approach. Multi-disciplinary team support for the trial was present in principle, yet equipoise was sometimes lacking for individual patients or among influential multi-disciplinary team members (Table 4:4). Some sites, with highly committed principal investigator support, recruited few patients, with lack of multi-disciplinary team support identified as an influential barrier.

Some principal investigators/multi-disciplinary teams ruled out referral for stereotactic radiosurgery entirely or for specific patients. We found consensus that evidence supporting the efficacy and safety of stereotactic radiosurgery was weaker than for excision, but surgeons adopted diverging positions in response. Whilst some were comfortable offering stereotactic radiosurgery in the research context, others ruled out stereotactic radiosurgery for symptomatic cerebral cavernous malformations, citing lack of evidence of benefit or concern about potential harms, although emotional issues were also acknowledged as influential (Table 4:5). These obstacles were discussed during an Investigator meeting (Table 1, September 2022) and recruiters were directed to the best available evidence to counter strong opinions.

Three sites specialising in paediatrics reported multi-disciplinary team consensus precluding referral of children for stereotactic radiosurgery, particularly those under 5 years, given the lack of evidence on potential long-term harm associated with radiation. Management recommendations defaulted to medical management if excision risks were judged too high (Fig. 2a and b). This combined with a relatively low threshold for excision of accessible lobar cavernous malformations in children, resulted in relatively few paediatric patients for whom there was equipoise, and there being only four paediatric recruits to the trial (Table 4:6).^13^ Nonetheless, these paediatric participants were recruited effectively across three sites. Analysis of paediatric recruitment discussions, found a highly effective strategy for presenting the trial was to frame it as a solution to the dilemma of which treatment pathway to follow, given uncertainty regarding management. Insights were shared with all sites in Tips and guidance documents (v2, March 2022, Appendix pp 9–12) and at Investigator meetings.

#### Diagnosis and treatment logistics

Determining eligibility for the trial frequently entailed repeat scans, re-referral for multi-disciplinary team discussion or multiple patient appointments, many appointments lasting more than 45 min. QRI-screening log free-text and recruitment discussion data indicated that some patients, presumed symptomatic and approached, were subsequently excluded as ineligible. Confirming eligibility took longer in those presenting with seizures, given work-up involved (Table 4:7). Some eligibility criteria raised controversy: some argued that headache was indicative of micro-haemorrhage, regardless of whether a bleed was scan-detected, and it was therefore inappropriate to exclude those presenting with headache (Table 4:8). Some patients experienced repeat intracranial haemorrhage, causing multi-disciplinary teams/surgeons to lose equipoise and regard neurosurgical excision as necessary (Fig. 2a and b). Repeat intracranial haemorrhage caused delay to stereotactic radiosurgery intervention delivery: haemorrhages needed time to recede, meaning treatment might occur outside the 3-month period for intervention delivery.

Stereotactic radiosurgery for treatment of symptomatic cavernoma was available at only two UK sites. At all other sites referral to one of these was needed to confirm patient suitability. Site teams raised queries about operationalising this and CARE Chat video 11 clarifying this process was disseminated in January 2022 (Fig. 1, Table 1). Usual care referral criteria for stereotactic radiosurgery differed from CARE pilot trial criteria: usual referrals required evidence of haemorrhage; trial criteria allowed randomisation of patients with focal neurological deficit alone. Qualitative findings indicated that multi-disciplinary teams excluded patients who showed focal neurological deficit alone (Table 4:9), despite the latter being eligible.

Patients referred for stereotactic radiosurgery assessment prior to sites opening, attended stereotactic radiosurgery clinics anticipating discussion about stereotactic radiosurgery. Recruiters found it challenging to engage these patients in a discussion about uncertainty and trial participation. Surgeons presenting the trial to patients referred from external sites for stereotactic radiosurgery were aware they had to manage expectations of referring neurosurgeons (Table 4:10), who might not support the trial.

Some surgeons argued that equipoise between conservative and surgical management could only be demonstrated with longer-term follow-up.^22^ Shorter follow-up, within the lifespan of a trial, captured short-term harms associated with neurosurgery but not long-term benefits, in comparison with medical management. Challenges in capturing long-term benefit were also raised in comparing neurosurgery with stereotactic radiosurgery, given that the latter was argued to show fewer short-term harms (Table 4:11).

The COVID-19 pandemic resulted in delays opening sites: 32 sites were due open by June 2021, only 14 were activated by December 2021 and 28 by close of recruitment in April 2023.^13^ COVID-19 related measures created additional barriers to patients accepting allocation to neurosurgery. Levels of anxiety and preoperative preparation increased with the need to self-isolate and the increased risk of cancellation, making stereotactic radiosurgery a more attractive option than neurosurgery for patients where intervention was recommended (Table 4:12).

### Facilitators and actions to enhance these

#### Commitment of the chief investigators, principal investigators and patient organisations

There was substantial commitment from the chief investigators, demonstrated by systematic communication with principal investigators during early recruitment via pre-recorded video messages on key issues (‘CI Chats’) and monthly online ‘CARE club’ meetings to address investigator questions (Table 1). Valuable support was given to recruitment by Cavernoma Alliance UK/Cavernoma Ireland colleagues to patients considering participation.^12^

#### Changing practice within the context of the trial

Some neurosurgeons were comfortable changing practice within the trial context: they offered randomisation between medical management and neurosurgery/stereotactic radiosurgery to those patients to whom they would usually recommend medical management outside the trial. These surgeons argued that the trial context, underpinned as it was by community equipoise, justified lowering the intervention threshold, with the explicit aim of broadening the pool of eligible patients and contributing to a future evidence base (Fig. 2a and b, Table 4:13). Adoption of this position by site principal investigators was necessary but not sufficient for optimising recruitment, but multi-disciplinary team decision-making overrode principal investigators at several sites.

#### Multi-disciplinary team equipoise

In CARE, 40 recruits (66% of target) came from four high recruiting sites (Table 2). All four sites reported either i) principal investigator equipoise and influence over multi-disciplinary team decision-making or ii) equipoise within the multi-disciplinary team and an informed decision-making approach to cavernoma management that explicitly included consideration of stereotactic radiosurgery (Table 4:14). Joint outpatient clinics involving neurosurgeons and neurologists took place at two sites and may have increased efficiency of recruitment, but numbers were too small to be definitive.

#### Offering stereotactic radiosurgery justified by evidence of lower risk

Whilst some surgeons rejected stereotactic radiosurgery given current lack of evidence regarding outcomes, others argued that the documented harms associated with stereotactic radiosurgery were less severe than for, or comparable to excision. These lower risks enabled these recruiters to feel comfortable offering randomisation within the trial with stereotactic radiosurgery as the intervention arm, with this explicitly justified by evidence of lower risk, rather than evidence of potential benefit (Table 4:15). This perspective, combined with lower patient thresholds for intervention using stereotactic radiosurgery as compared to excision (Table 4:16) explained the larger proportion of recruits for whom stereotactic radiosurgery was the preferred intervention arm (n = 44, 61%) as compared to excision (n = 19, 26%) or no preference (n = 9, 13%).^13^

Amongst surgeons comfortable with randomisation to stereotactic radiosurgery, additional controversy arose about whether stereotactic radiosurgery should be offered to those presenting with seizures. Most doing so, argued patients should know that stereotactic radiosurgery was offered to reduce haemorrhage risk, not for seizure control. Offer of stereotactic radiosurgery to those presenting with seizures with no evidence of haemorrhage was discussed during Investigator meeting 3. Discussion acknowledged that eligibility criteria permitted this, but it remained controversial (Table 4:17).

#### Patient preferences

Although patient preferences were most likely to default to medical management, some patients perceived the trial as an opportunity to access surgical interventions not previously offered. Patients with problematic seizure symptoms interpreted discussion about the trial as an opportunity to request surgical interventions they had hitherto understood to be unavailable. In parallel, some recruiters reported that information provision about the trial resulted in patients requesting surgical intervention, something not experienced when the trial was not discussed (Table 4:18). These findings highlighted the need for delicate balancing of information provision and constant monitoring for patient understanding during trial discussions: some patients declined trial participation (preferring surgical intervention) and one patient accepted randomisation then declined the allocation (to medical management), choosing neurosurgery instead.^13^ The QRI team led individual and trial wide feedback (Table 1: CARE Chat 10, Tips and Guidance document see Appendix pp 12–21, Investigator meeting 3) encouraging recruiters to establish that all patients were comfortable accepting either trial arm if allocated, prior to randomisation.

## Discussion

The CARE-QRI revealed that whilst conventions of usual care for symptomatic cerebral cavernous malformation functioned as a barrier to clinician equipoise, it was nevertheless possible for surgeons to move away from usual care practice, offering surgical intervention within the trial to people, to whom they would otherwise recommend medical management. The multi-disciplinary team overrode principal investigator equipoise at some sites; highest recruitment occurred where principal investigators controlled multi-disciplinary team decision-making or where the multi-disciplinary team had equipoise. Patient preferences generally defaulted to minimally invasive management (medical management or stereotactic radiosurgery), however patient preferences originated with perceived clinician recommendations more frequently than clinicians realised. Patients countered clinician expectations by being in equipoise despite time having elapsed since symptom presentation or using discussion of the trial to request surgical intervention. The eligible patient pool was smaller for paediatric participants, yet it was possible to recruit these if the trial was presented as a solution to the dilemma of uncertainty regarding management. The leadership of the chief investigators, trial team, principal investigators and support of Cavernoma Alliance UK/Cavernoma Ireland combined with actions to promote effective communication about the trial and clinician equipoise, enabled the recruitment target to be exceeded following a short extension.^13^

Clear differences marked the approach of clinicians who gained a position of clinical equipoise and recruited effectively: i) being comfortable offering participation in the trial to patients to whom they would otherwise recommend medical management; ii) being prepared to offer stereotactic radiosurgery in the intervention arm. Both positions were viewed as acceptable within a trial context. Offer of stereotactic radiosurgery was justified not with reference to evidence of benefit, but to evidence of lower risk of iatrogenic harm compared with neurosurgery.

The pool of patients for whom there was equipoise was broader for adults than children, resulting in only four recruits below 18 years.^13^ Conventions of usual care led children to whom neurosurgery would be recommended outside the trial being removed from the eligible patient pool, leaving a small group of patients at high risk of morbidity from haemorrhage and for whom neurosurgical excision was similarly high risk. Adult patients in this category were more likely to be recommended for randomisation between medical management and stereotactic radiosurgery, given high risks of morbidity of a re-bleed and lower risks of morbidity associated with stereotactic radiosurgery. Patient preferences for less invasive procedures mirrored recruiter preferences for the perceived lower risk intervention, combining to give the larger proportion offered randomisation with stereotactic radiosurgery as the intervention arm.^13^

Concerns about duration of follow-up required to demonstrate benefits of surgery over medical management have been raised elsewhere.^22^ Recruitment challenges identified have been previously documented: surgeons recommending a particular management pathway rather than being comfortable offering randomisation^23^; conflict between usual care vs trial pathways, its negative impact on equipoise, and the influence of the multi-disciplinary team.^24^ Previous QRI work found training enabled recruiters to become more comfortable with equipoise and uncertainty over time.^25^^,^^26^^,^^27^ Here the influence of the multi-disciplinary team was decisive and trumped any preparedness of individual principal investigators to offer participation to patients.

Limitations include that participants in qualitative data collection were supportive of the trial; negative views may not be so well documented. Failure to reach conclusions on influences of some variables (age, presentation with seizure vs bleed or both, time since cavernoma presentation) was unavoidable, given challenges of conducting a QRI within a pilot trial, designed to include a wide range of participants and observe how neurosurgeons operationalised eligibility criteria. Timing of the study immediately post the COVID-19 pandemic added logistical challenges, with other UK trials experiencing similar issues.^28^ Given this context, recruitment of 72 patients (target 60) with a mean recruitment rate of 0.2 participants per site month (target 0.114 participants per site month^13^) represented a substantial achievement. Intensive QRI-informed actions during months 1–7 of recruitment, combined with the commitment of the chief investigators and several influential principal investigators, led to improvement in recruitment months 8–20.

A definitive trial will require recruiters, including multi-disciplinary team members, receiving support to feel comfortable offering randomisation to patients for whom medical management or surgical management would conventionally be offered. This pilot trial found randomisation between medical management and stereotactic radiosurgery more feasible from the perspective of surgeons and patients than between medical management and neurosurgery. Yet the latter should not be ruled out as unacceptable to patients: some patients perceived the trial to be a gateway to receiving surgical intervention and recruiter training should raise surgeon awareness of such patients. Randomisation of children was more challenging, given the smaller pool of patients for whom there was equipoise. Yet inclusivity and pragmatism remain important principles in trials research^29^ and three recruiters within CARE successfully recruited children using effective information provision.

In conclusion, the CARE-QRI revealed how conventions of usual care, multi-disciplinary team decision-making and logistical issues created challenges for recruitment, and how targeted interventions by the QRI team, the chief investigators, principal investigators and trial management group could address these challenges to achieve recruitment that exceeded the target with a 2-month extension. Insights and actions to facilitate recruitment will inform a definitive trial.

## Contributors

JW and JLD designed the QuinteT Recruitment Intervention for the CARE pilot phase trial in collaboration with RA-SS. JW, NF, AXR and JLD implemented the QuinteT Recruitment Intervention, in collaboration with RA-SS (chief investigator of the CARE pilot trial) and NK (co-chief investigator of the CARE pilot trial) and the CARE pilot trial management group. JW, NF, AXR and JLD accessed and verified the underlying data. LF, KAH, PJAH, SCL, JJML and JS contributed to data interpretation and implementation of QuinteT Recruitment Intervention actions. RA-SS (chief investigator) and NK (co-chief investigator) conceived the idea for the CARE pilot phase trial. RA-SS (lead applicant) obtained funding, developed the protocol and implemented the CARE pilot trial with the trial management group. All authors reviewed the analyses and drafts of this manuscript, and approved the final version.

## Data sharing statement

Other researchers may apply for access to a de-identified version of the dataset used for analysis via QuinteT@groups.bristol.ac.uk following publication. Written proposals will be assessed by the University of Bristol Data Access Committee and a decision made about the appropriateness of the use of data. A data sharing agreement may need to be put in place before any data are shared.

## Declaration of interests

JW reports funding from the National Institute for Health and Care Research for this project, paid to the University of Bristol and membership of the National Institute for Health and Care Research, Research for Patient Benefit South West general funding committee. KAH reports an unrestricted educational grant from Medtronic for delivery of Stroke CT imaging day 2023 and is Clinical Lead of the South Yorkshire Integrated Stroke delivery Network. NK reports receiving personal income from consultancy with Amethyst UK Limited. RA-SS reports funding from the National Institute for Health and Care Research for this project, paid to The University of Edinburgh; funding from the Chief Scientist Office Health Improvement, Protection and Services Research Committee, Project Grant Platform randomised controlled trial for INTracerebral Haemorrhage (PLINTH): community-based feasibility study. Ref. HIPS/22/36, paid to The University of Edinburgh; consulting fees from Recursion Pharmaceuticals and Bioxodes, paid to The University of Edinburgh; participation in endpoint adjudication for Novo Nordisk, paid to the University of Edinburgh. SCL reports membership of the NIHR Health Technology Assessment general funding committee, 2016–21. JJML reports fellowship funding from the Wellcome Trust paid to the University of Edinburgh and covering his salary. The rest of the writing group declare no interests.

## Appendix A Supplementary data

CARE QRI Supplemental data

CARE Protocol V2.0 (22Mar2021).pdf

Protocol paper
