# Supplementary material for: Addressing barriers and identifying facilitators to support informed consent and recruitment in the Cavernous malformations A Randomised Effectiveness (CARE) pilot phase trial: insights from the integrated QuinteT recruitment intervention (QRI)
Source: eClinicalMedicine. 2024 Apr 18;71:102557. doi: 10.1016/j.eclinm.2024.102557 (PMC11133797; doi:10.1016/j.eclinm.2024.102557)
Supplement: CARE QRI Supplemental data [file mmc1.docx]

**Supplementary file captions:**

Consolidated criteria for reporting qualitative studies (COREQ)

Table comparing processes, benefits and risks of interventions compared within the CARE pilot trial presented in the CARE participant information leaflet. (CARE Trial - Supplementary PIL (Adult) 22Mar2021 V2.0)

QRI Tips and guidance document circulated to recruiters and site teams (v2 March 2022)

QRI Tips and Guidance documents circulated to recruiters and site teams (v3 Dec 2022)

CARE-QRI Collaborators List for PubMed

Collaborators on delegation logs at hospital sites that recruited at least one participant to the CARE pilot trial

**Consolidated criteria for reporting qualitative studies (COREQ): 32 item checklist applied to qualitative data collection, analysis and reporting within the CARE pilot trial QuinteT Recruitment Intervention (QRI). [Tong et al. 2007]**

| **Domain 1: Research team and reflexivity** | |  |
| --- | --- | --- |
| Personal Characteristics | |  |
| 1. | Interviewer/facilitator | Which author/s conducted the interviews or focus groups (‘workshops’)?  JW, JLD, NF, ARR |
| 2. | Credentials | What were the researchers’ credentials?  JW, JLD, NF, ARR all experienced post-doctoral researchers specialising in qualitative methods. |
| 3. | Occupation | What was their occupation at the time of the study?  JW: Senior Lecturer in Qualitative Health Science applied to Randomised Controlled Trials; JLD: Professor of Social Medicine; NF: Research Associate, QuinteT team; ARR: Research Fellow, QuinteT team. |
| 4. | Gender | Was the researcher male or female?  All female |
| 5. | Experience and training | What experience or training did the researcher have?  JW, JLD, NF, ARR had experience in qualitative data collection, analysis and reporting in general and all had experience in application of the data collection, analysis and reporting methods used with the QuinteT recruitment intervention in particular (18, 23, 2, 7 years’ experience respectively of applying the QRI to trial recruitment). |
| Relationship with participants | |  |
| 6. | Relationship established | Was a relationship established prior to study commencement?  None of the health professional or patient/family participants were previously known to the researchers. |
| 7. | Participant knowledge of the interviewer | What did the participants know about the researcher? e*.g. personal goals, reasons for doing the research*  All participants knew that researchers were employed to optimise recruitment and informed consent processes within the CARE pilot trial. |
| 8. | Interviewer characteristics | What characteristics were reported about the interviewer/facilitator? e.g. *Bias, assumptions, reasons and interests in the research topic*  Researcher occupation and specialist knowledge in the research methodology were reported. |
| **Domain 2: study design** | |  |
| Theoretical framework | |  |
| 9. | Methodological orientation and Theory | What methodological orientation was stated to underpin the study? *e.g. grounded theory, discourse analysis, ethnography, phenomenology, content analysis*  The QRI is a pragmatic intervention: it uses data collection and analysis methods in line with what is most appropriate for the research objective^1^. Data collection and analysis were informed by ethnography, phenomenology, and grounded theory^2^ and combined reflexive thematic analysis^3,4^, content analysis^5^ and targeted conversation analysis^6^. |
| Participant selection |  |  |
| 10. | Sampling | How were participants selected? *e.g. purposive, convenience, consecutive, snowball*  Sampling was in part purposive and in part opportunistic: 18 sites were supplied with audio recorders and recruiters were encouraged to invite all potential participants to consent to audio-recording the discussion. Healthcare professionals and patients who had consented to the audio-recording of their recruitment discussions were then purposively sampled and invited to take part in interviews. Healthcare professionals were purposively sampled to include a range of views, practice, study site, professional background and grade.  Patients were purposively sampled to include those who had declined participation across a range of study sites and those who had accepted participation only to decline the allocated intervention. |
| 11. | Method of approach | How were participants approached? e*.g. face-to-face, telephone, mail, email*  Healthcare professionals were approached via email.  Patients were approached via email and/or telephone call. |
| 12. | Sample size | How many participants were in the study?  20 health care professionals were audio recorded during recruitment consultations.  28 healthcare professionals took part in interviews or focus group (‘workshop’) discussions.  71 patients were audio-recorded during recruitment discussions.  11 patients were interviewed. |
| 13. | Non-participation | How many people refused to participate or dropped out? Reasons?  One healthcare professional agreed to be audio-recorded during recruitment consultations but did not respond to invitations to take part in an interview.  71 of a possible 212 patients agreed to audio-recording of recruitment discussions (note no report was available on how many of these 212 were in fact invited).  No participants contacted to take part in interviews declined the opportunity or withdrew. |
| Setting |  |  |
| 14. | Setting of data collection | Where was the data collected? e*.g.* Healthcare professionals were interviewed via Teams in a location of their choice.  Patients were interviewed via telephone in a location of their choice. |
| 15. | Presence of non-participants | Was anyone else present besides the participants and researchers?  Some patient participants had partners or family members present during interviews and these were subject to the same consent processes as the patient participants. |
| 16. | Description of sample | What are the important characteristics of the sample? *e.g. demographic data, date*  The healthcare participants were sampled across sites with a range of performance in terms of numbers screened, approached or recruited. Patients were sampled from those declining participation and those accepting participation but declining the allocation. |
| Data collection |  |  |
| 17. | Interview guide | Were questions, prompts, guides provided by the authors? Was it pilot tested?  Interview topic guides were used and are available on request. Questions were developed in the context of i) previous research to support recruitment in trials; ii) specific issues relevant to the CARE pilot trial. |
| 18. | Repeat interviews | Were repeat interviews carried out? If yes, how many?  Each participant gave at least one interview. Two healthcare participants gave two interviews each. Some healthcare participants participated in both focus groups and individual interviews. There were no repeat patient interviews. |
| 19. | Audio/visual recording | Did the research use audio or visual recording to collect the data?  A digitally encrypted audio-recorder was used to collect audio-recordings of recruitment consultations and patient interviews. |
| 20. | Field notes | Were field notes made during and/or after the interview or focus group?  Field notes were collected at each interview and focus group discussion. |
| 21. | Duration | What was the duration of the interviews or focus group?  The interviews and workshops lasted between 30 -90 minutes |
| 22. | Data saturation | Was data saturation discussed?  There were some areas on which saturation was not reached. These are reported where relevant in the findings and highlighted in the discussion. |
| 23. | Transcripts returned | Were transcripts returned to participants for comment and/or correction?  No transcripts were returned to participants for correction. Two healthcare professionals were contacted via email for further clarification about statements made during interviews. |
| **Domain 3: analysis and findings** |  |  |
| Data analysis |  |  |
| 24. | Number of data coders | How many data coders coded the data?  There were three data coders. |
| 25. | Description of the coding tree | Did authors provide a description of the coding tree?  Coding is available on request. |
| 26. | Derivation of themes | Were themes identified in advance or derived from the data?  The reflexive thematic analysis combined deductive and inductive analyses. Themes were derived from the newly collected data but informed by the QuinteT team’s previous work on barriers to recruitment within trials. |
| 27. | Software | What software, if applicable, was used to manage the data?  NVivo12 |
| 28. | Participant checking | Did participants provide feedback on the findings?  Patient participants were sent a summary of findings. Healthcare professionals will be sent a copy of this publication. |
| Reporting |  |  |
| 29. | Quotations presented | Were participant quotations presented to illustrate the themes / findings? Was each quotation identified? e*.g. participant number*  Participant quotations were used to illustrate the findings and each quotation is identified by a participant number. |
| 30. | Data and findings consistent | Was there consistency between the data presented and the findings?  Quotations are presented in Table 4 and were selected to support the reported findings. |
| 31. | Clarity of major themes | Were major themes clearly presented in the findings?  The findings are presented as two major themes: barriers (with associated actions); facilitators (with associated actions). |
| 32. | Clarity of minor themes | Is there a description of diverse cases or discussion of minor themes?  Outliers have been included within the report, e.g. preparedness to randomly allocate patients presenting with seizures to medical management or stereotactic radiosurgery. |

1.Seale, C. (1999). Quality in Qualitative Research. *Qualitative Inquiry*, *5*(4), 465-478. <https://doi.org/10.1177/107780049900500402>

2. Rooshenas L, Paramasivan S, Jepson M, Donovan JL. Intensive Triangulation of Qualitative Research and Quantitative Data to Improve Recruitment to Randomised Trials: The QuinteT Approach. Qual Health Res 2019;29(5):672-9.

3. Braun V, Clarke V. Using thematic analysis in psychology. Qual Res Psychol2006;3:77-101. doi:10.1191/1478088706qp063oa.

4. Saunders CH, Sierpe A, von Plessen C et al. BMJ 2023;381:e074256 http://dx.doi.org/10.1136/bmj-2022-074256

5. Krippendorf, K. (1980). Content analysis: An introduction to its methodology. Beverly Hills, CA: Sage

6. Wade J, Donovan JL, Lane JA, Neal DE & Hamdy F. It's not just what you say, it's also how you say it: opening the 'black box' of informed consent appointments in randomised controlled trials. *Social Science & Medicine*. 2009;68(11)2018 – 2028.

**Table comparing processes, benefits and risks of interventions compared within the CARE pilot trial presented in the participant information leaflet. (CARE Trial - Supplementary PIL (Adult) 22Mar2021 V2.0)**

|  | **Treatment without surgery** | **Treatment including surgery** | |
| --- | --- | --- | --- |
|  |  | **Neurosurgery** | **Stereotactic radiosurgery** |
| **What may be involved?** | - Treat symptoms - Prevent seizures - Rehabilitation - Brain scan | - Treat symptoms - Prevent seizures - Rehabilitation - Brain scan | - Treat symptoms - Prevent seizures - Rehabilitation - Brain scan |
|  |  | - Hospital admission for days - General anaesthetic - Opening in the skull - Operation to remove cavernoma - Follow-up brain scan - Must not drive for 6 months | - Hospital attendance for a day - Anaesthetic not needed - Head fixed in a temporary frame - Focussed radiation given once - Follow-up brain scans |
| **What are the possible benefits?** | - Bleed/stroke risk reduces as time passes - Avoids risks of neurosurgery or radiosurgery | - Risk of bleed/stroke lower if cavernoma removed - Less worry about symptoms returning | - Risk of bleed/stroke may be lower if cavernoma stabilised, but these benefits are uncertain - Less worry about symptoms returning |
| **What are the possible risks?** | - Future bleed/stroke due to cavernoma - Can be mild - May be disabling - Rarely be fatal - Risk higher for cavernoma in brainstem | - Bleed/stroke due to neurosurgery - Can be mild - May be disabling - Rarely be fatal - Risk higher for cavernoma in brainstem | - Bleed/stroke despite radiosurgery - Can be mild - May be disabling - Rarely be fatal - Risk higher for cavernoma in brainstem |
|  | - Epileptic seizures, which may be difficult to control | - Epileptic seizures may not go away | - Epileptic seizures may not go away |
|  | - Cavernoma remains in the brain, so the risks of stroke and seizure may never go away | - Cavernoma may come back | - Cavernoma not removed |
|  | - Worry about symptoms returning | - Complications of treatment (e.g. infection or damage to brain around the cavernoma) | - Complications of treatment (e.g. damage to brain around the cavernoma) |


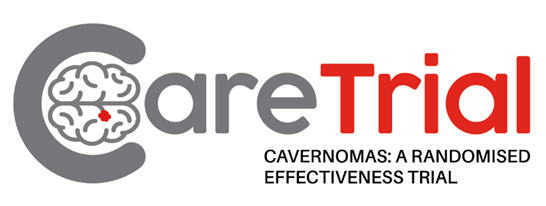
**QRI Tips and guidance document circulated to recruiters and site teams v2 March 2022**

| **Tips for recruitment and informed consent discussions March 2022** |
| --- |
| - Please approach **all patients with** **one or more symptomatic brain cavernomas** for a discussion about taking part. - This document gives suggestions to support informed consent and recruitment, including suggestions for wording which have been observed to benefit recruitment discussion. - You will have your own preferred style of giving information. Suggestions here can complement your own standard approach. |
| **Introduce the CARE study early in discussions** |
| **Purpose of discussion:** give information about the cavernoma and options for management, including taking part in the CARE study.  *“The decision we’re trying to make is whether we operate to remove the cavernoma that has caused your symptoms, or whether we decide not to operate and continue to monitor it. There is uncertainty about which of these is best for you. The CARE study is a clinical trial and if you take part, you will have a 50:50 chance of being allocated to either treatment including surgery or treatment without surgery.”*  **Key information** reassures people that the study is supported by people with cavernoma, specialist surgeons/doctors and the UK government:   - **CAUK / Cavernoma Ireland** support the study and have helplines available to answer questions. - **JLA priority setting**: the study addresses the question identified as the top priority for cavernoma research by people with cavernoma, their families and specialist surgeons/doctors. - **UK government-funded**, large international study, with sites across the UK / Republic of Ireland. |
| **Discussing options** |
| - **MDT**: Let the patient/family know that the MDT has agreed that *treatment including surgery* and *treatment without surgery* are both suitable options in their case. - **Order of presentation**: Many patients declining the study are continuing without surgery, so potential benefits of surgery need to be clearer. Try introducing surgery first and describing benefits of surgery early on, as well as the risks:   *“Surgery aims to remove the cavernoma to prevent bleeds/seizures in future. Treatment without surgery monitors you, treats any symptoms if you do have a bleed.”*  *The idea of doing surgery is very much to see if we can reduce your risk of having a bleed in the future, so to improve your quality of life in the future.*   - **Balance of risks**: the upfront risks of surgery are similar to the longterm risks for treatment without surgery – the key message is that these risks balance each other out. - **Use the CARE supplementary PIL**, in particular the table showing how treatment without surgery and treatment including surgery compare for what is involved, what the benefits are and what the risks are.  \| *“In surgery the risk is upfront, so your biggest risk is at the time of surgery. Whereas without surgery, your risk is spread out over several years.”*  *“In [patient’s] case her cavernoma is very close to these motor areas so I think her risk of neurological worsening is there with surgery. But equally if she were to have another bleed, she could also develop neurological weakness down that side, and we don’t know if she would recover or not.”* \| \| --- \|  - It can really help patients/families if you explicitly acknowledge the uncertainty about treatment options and how hard this can be to deal with.   **Acknowledging uncertainty**   - Being uncertain can leave patients, families and you feeling uncomfortable and looking for a way out of the dilemma of what to do. - Participation in the study can provide a clear direction and a way to manage this discomfort.   *“The hardest thing for people is to understand the concept of uncertainty. You know the whole reason we’re doing the trial is because we’re uncertain. And if we knew the right answer, we wouldn’t need the study.”*  *“I get the impression you are a bit like me, that you’re not sure either and that’s why it is so difficult. And I think my advice in that setting, is sometimes to enter the trial, as it does take some of that responsibility for making the decision away, in that sense. Because the trial itself deals with the uncertainty, does that make sense?”*  *“You can say, there was uncertainty and I entered a clinical study that randomised you to one or other treatment, because the doctors didn’t know at that time, which the best treatment was. And it takes you one step away from actively making a decision.”*   - You can acknowledge the potential benefits of taking part in a study like the CARE study, which include direct access to the research nurse team and a 6-month follow up scan.   **Randomisation**  *“I guess the other thing to say about clinical trials is that you usually get good information when you’re involved in clinical trials, you usually get good monitoring and access to the research nurses here, to support you and so on. So, there are soft advantages of being in studies in that there is support and people that you can ask questions to as things go on.”*  **Benefits of participation**  **Explaining the purpose of randomisation is crucial:**   - To achieve a fair comparison between treatments as we don’t know which is best. - Randomisation is the only way to create 2 groups of people that are as similar as possible, apart from which treatment they receive. - Patients will have an equal chance of being allocated to treatment including surgery or treatment without surgery   **Process of randomisation**: be clear that the computer is not ‘choosing the best option’   - Although a computer is used to allocate the treatment they will receive, it is a chance allocation and does not take into account any of the person’s medical history or clinical characteristics.   **Terminology and positive framing**   - We recommend using the terms **decision/decide, options/opt** but **avoiding** **choose/choice** as people can hear this as being encouraged to develop a preference and choose an intervention. - Avoid presenting the study as ‘*only for people who are unable to choose’* between the 2 interventions; present it as a positive solution to a dilemma, given the lack of evidence. - Patients are often frightened by the prospect of surgery, so emphasise the benefits of surgery, which aims to prevent a future bleed/recurrence of symptoms, as well as the risks. - When describing treatment without surgery, **avoid referring to it as *leave it be*, *leave things alone*, *carry on as you are*** and instead present it as an active decision to continue to have *treatment without surgery* - Avoid presenting **randomisation** in a negative light (i.e. focusing on the lack of control/choice) or only for people who cannot choose between treatments. Present it as a solution to the dilemma, given the uncertainty as to which option is best. - Use ‘*We’* to convey the (MDT) team judgement that each of these options is suitable and use **positive framing** wherever possible:  \| **Positive framing** \| \| --- \| \| *“We know your cavernoma has caused your symptoms. What we don’t know is what is the best option for preventing this happening again in future.”* \| \| *“We know the MDT agrees that both treatment including surgery and treatment without surgery are suitable options for you.”* \| \| *(For neurosurgery) “Both options are standard options that have been used in treating cavernoma for many years.”* \| \| *“The benefit of surgery is that it aims to prevent your symptoms returning. The benefit of treatment without surgery is that it avoids surgery. Both options come with risks, but those risks are very evenly balanced.”* \| \| *“Am I right in thinking that like me, you are not sure which option is best? In those cases, my advice is to enter the study as it shares the responsibility for making the decision.”* \| |
| - Check that the patient feels comfortable accepting either *treatment including surgery* or *treatment without surgery* to join the CARE study and be randomised.   **Closing the discussion**   - **Ask for written consent to use the audio recording for analysis in the Information Study at the end of your discussion as this will enable you to upload the audio recording.** |


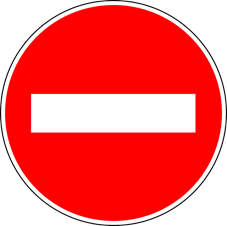
**Thank you for your help in explaining the CARE study to people with cavernoma and their families. This help is very much appreciated by the CARE study team and will in future benefit all patients being invited to join the study.**

**This document is a work in progress.**

**Feel free to note down questions/issues/solutions to share with colleagues and forward them to** [**Julia.Wade@bristol.ac.uk**](mailto:Julia.Wade@bristol.ac.uk)

**QRI Tips and Guidance documents circulated to all site teams (v3 December 2022)**


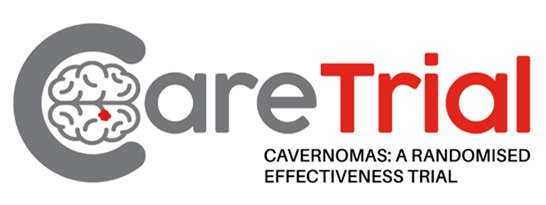


Tips and Guidance: Recruiting to CARE

Structuring your conversations about CARE

- You will have established patterns of how you communicate potential treatment options with patients. This guidance is not intended to override these skills, instead to offer a structure which may help to explain research to patients in general and help with CARE specifically.

Unpacking a patient’s understanding of brain cavernoma and management options

- It can be helpful to understand what pre-conceptions the patient has about their prognosis and treatment options.
- Some patients may have beliefs about their suitability for certain treatments, or mis-conceptions about what is available to them.
- Understanding these early can help tailor the discussions you have with patients about CARE.

Introduce options for management including the CARE study

“The decision we’re trying to make is whether we use surgery to treat the cavernoma that has caused your symptoms, or whether we decide not to use surgery. There is uncertainty about which of these is best for you. The CARE study is a clinical trial and if you take part, you will have an equal opportunity of being allocated to either treatment including surgery or treatment without surgery.”

- Briefly explain that the CARE study is comparing treatment including surgery and treatment without surgery for their cavernoma, because there is uncertainty about which of these options is best amongst the community of doctors and surgeons.
- The study is UK government-funded, with sites across the UK/Republic of Ireland and supported by Cavernoma Alliance UK.

Acknowledge that uncertainty can be uncomfortable

- A team of doctors (the MDT) has discussed their case and agreed that either treatment including surgery or treatment without surgery are equally suitable options for them.
- Being uncertain can leave patients, families and you feeling uncomfortable and looking for a way out of the dilemma of what to do.

“I get the impression you are a bit like me, that you’re not sure either and that’s why it is so difficult. And my advice in this setting is to enter the trial, as it does take some of that responsibility for making the decision away from us, because the trial itself deals with the uncertainty. Does that make sense?”

- Participation in the study can provide a clear direction and way to manage this discomfort.
- Briefly note that there are benefits and risks to both treatment with surgery and treatment without surgery, which you will cover.

"The conclusion from the multidisciplinary team meeting is that both treatment with surgery and treatment without surgery are equally good, for somebody like you. They both have advantages. And they both have downsides."

Explain management options

- If the patient has already indicated a preference for either treatment with surgery or treatment without surgery, **explain the non-preferred option first**.
- When describing the treatment options, present the **benefits** of treatment **before** discussing the **risks**.
- Use the table provided in the **CARE Supplementary Patient Information Leaflet** to guide your discussions about the treatment options.
- Balance of risks: the upfront risks of surgery are similar to the long-term risks for treatment without surgery. The key message is that these risks balance each other out.

“The idea of doing surgery is very much to see if we can reduce your risk of having a bleed (or epileptic seizure) in the future, so to improve your quality of life in the future. The idea of treatment without surgery is to manage any symptoms now or in future.”

Explain neurosurgery or stereotactic radiosurgery (SRS) as appropriate to the individual patient.

- If possible, be clear prior to the consultation whether you would recommend (1) neurosurgery, (2) SRS, or (3) either neurosurgery or SRS for the individual patient.
- Highlight that neurosurgery/SRS are intended to reduce the future risk of a bleed (or seizure or FND if applicable).
- The risks of neurosurgery/SRS are similar to the risk of a serious bleed if the cavernoma is not treated with neurosurgery/SRS
- We aren’t certain whether treatment including surgery or treatment without surgery is better, but:
  - neurosurgery techniques are well-established and the surgeon operating will have plenty of experience performing this type of surgery.
  - SRS techniques have been used for several decades and evidence indicates a balance between the risks of leaving the cavernoma and the risks of having SRS.
- For more information on the process of referring patients for SRS in CARE see [here](https://media.ed.ac.uk/media/1_clduu9f1).

Explain medical management

- The benefit of medical management is that any of the risks of neurosurgery/SRS are avoided
- However, this means that there is a continued risk of a bleed – and we cannot say for sure what the impact of a future bleed might be, or how likely it is to occur.

Explain purpose and process of randomisation

- When describing randomisation, it is important to explain both the **purpose** and **process** of randomisation.
- The **purpose** of randomisation is to **create comparable groups** which enables us to make a **fair comparison** between the two groups in the CARE pilot trial.
- Treatment is allocated at random rather than chosen, to avoid bias from the doctor or patient selecting a treatment.
- When describing the **process** of randomisation, it is helpful to avoid explaining it in a way which gives the impression the computer has ‘agency’ to make a decision about which treatment is best for the patient. Be aware that some patients perceive analogies such as ‘*tossing a coin’* or ‘*shaking a dice’* to be a flippant way to determine such a potentially life changing decision.
- The **process** can be described as a way of **providing an equal opportunity** to access either treatment option.
- **Randomisation** can be **framed positively**: to provide **a way out of the dilemma** left by the **uncertainty** as to which treatment option is best.

“You will have an equal opportunity of being allocated to either treatment including surgery or treatment without surgery”

“We use randomisation when we don’t know which treatment is best for the patient. It helps us to create equal groups of patients, without introducing bias”

Acknowledge and address patient preferences

- Patient preferences may be weaker than you think.
- Preferences are often based on a lack of knowledge or misunderstandings (e.g. unfounded fears of surgery, or perception that treatment without surgery is the ‘easy option’).
- Gently exploring patients’ preferences is part of the process of ensuring they are fully informed about their options.

Acknowledging preferences

- Using the expression “*Ok, but*” is a helpful way of acknowledging that you have listened to the patient’s preference but will balance these preferences with the reasons behind offering alternative options.
- Opening up the conversation can help to unpack the reasons behind preferences.

Addressing preferences

Patient: “I want treatment without surgery because I just want to carry on as I am.”

Recruiter: “**OK**, I understand why you might say that. **But** can I just check that you realise that for your cavernoma, surgery might be a very good option. Can I just check what your feelings are about surgery?”

- Exploring reasons underpinning patient preferences is an important part of making sure patients are making informed decisions.
- Patients may have general preferences, such as wishing to avoid surgery, without a full understanding of what surgery would entail.

Patient: “Well, I’ve had a lot of thoughts about the study and I’m **not altogether sure** but **I think** I want to go for treatment without surgery”

Recruiter: “Ok. You’d rather have treatment without surgery? So could you help me understand why that is?”

Patient: “Er, not really because, er I don’t really know enough about it to be able to, er, give you a serious reason. I just always prefer to avoid surgery if I can.”

Recruiter: “OK, that’s fine. Perhaps we could discuss what concerns
you have about surgery?”

Check willingness to accept randomly allocated arm

- Patients may have some degree of preference and still be willing to join CARE. This could stem from altruistic tendencies (wanting to help others with cavernomas) or a desire to access the increased follow-up and monitoring in CARE.
- It is key to ‘check in’ with patients prior to proceeding to randomisation that, despite these preferences, they would be willing to accept either of the arms within CARE if allocated. We suggest avoiding asking them if they have a preference: if they have a mild preference they will likely respond ‘yes’. Instead explore whether they feel able to accept either arm:

“Can I check that if you go forward with randomisation, you would be comfortable accepting allocation to either treatment including surgery or treatment without surgery?”

Acknowledge benefits of participation

- You can acknowledge the benefits of taking part in a study like the CARE study, which include direct access to the clinical and research team and a 6-month follow up scan.

“I guess the other thing to say about clinical trials is that you usually get good information when you’re involved in clinical trials, you usually get good monitoring and access to the research nurses here, to support you and so on. So, there are advantages of being in studies in that there is support and people that you can ask questions to as things go on.”

This information has been put together based on comments and experiences of your colleagues and is a work in progress. If you have comments or questions about this information or that arise as you discuss CARE with patients, we would like to hear them:

[Julia.Wade@bristol.ac.uk](mailto:Julia.Wade@bristol.ac.uk)

| - **Links to ‘CARE Chats’ that might help answer frequently asked questions** |
| --- |
| - [CARE Chat – How to complete screening logs](https://www.ed.ac.uk/usher/edinburgh-clinical-trials/our-studies/all-current-studies/care/care-study/faq/care-chats-screening-logs) |
| - [CARE Chat – Screen-as-you-go](https://www.ed.ac.uk/usher/edinburgh-clinical-trials/our-studies/all-current-studies/care/care-study/faq/care-chat-screen-as-you-go) |
| - [CARE Chat – Logistics of recruitment](https://www.ed.ac.uk/usher/edinburgh-clinical-trials/our-studies/all-current-studies/care/care-study/faq/care-chat-logistics-of-recruitment) |
| - [CARE Chat – Approaching patients diagnosed long ago & treated without surgery](https://www.ed.ac.uk/usher/edinburgh-clinical-trials/our-studies/all-current-studies/care/care-study/faq/care-chat-approaching-patients-diagnosed-long-ag) |
| - [CARE Chat – Tips for conversations about the CARE study](https://www.ed.ac.uk/usher/edinburgh-clinical-trials/our-studies/all-current-studies/care/care-study/faq/care-chats-tips-for-conversations-about-the-care) |
| - [CARE Chat – Latest top tips for recruitment conversations](https://www.ed.ac.uk/usher/edinburgh-clinical-trials/our-studies/all-current-studies/care/care-study/faq/care-chat-latest-top-tips-for-recruitment-conversa) |
| - [CARE Chat – Audio-recordings](https://www.ed.ac.uk/usher/edinburgh-clinical-trials/our-studies/all-current-studies/care/care-study/faq/care-chats-audio-recordings) |
| - [CARE Chat – Describing randomisation](https://www.ed.ac.uk/usher/edinburgh-clinical-trials/our-studies/all-current-studies/care/care-study/faq/care-chat-describing-randomisation) |
| - [CARE Chat – When to randomise](https://www.ed.ac.uk/usher/edinburgh-clinical-trials/our-studies/all-current-studies/care/care-study/faq/when-to-randomise) |
| - [CARE Chat - SRS](https://www.ed.ac.uk/usher/edinburgh-clinical-trials/our-studies/all-current-studies/care/care-study/faq/care-chats-srs) |


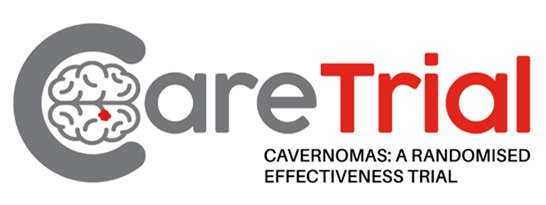


Tips and Guidance: Screening for CARE

Who is eligible for CARE?

1. People of any age

2. At **least one brain cavernoma** diagnosed by brain MRI that included a gradient echo or susceptibility-weighted sequence, according to standard diagnostic criteria

3. Clinical history attributable to a brain cavernoma of:

a. Symptomatic stroke **due to intracranial haemorrhage**, or

b. Symptomatic stroke due to a persistent or progressive non-haemorrhagic, or not otherwise specified, **focal neurological deficit**, or

c. **Epileptic seizure(s)** meeting the definition of definite or probable cavernoma-related epilepsy

4. Patient and doctor are uncertain about medical management or medical and surgical management of the symptomatic brain cavernoma, following consultation with a neurosurgeon

5. Patient has mental capacity to consent for themselves (adult participants or paediatric participants with capacity) or parent/legal guardian provides consent (paediatric participants).

**Patients must be considered equally suitable for EITHER medical management OR medical and surgical management (with neurosurgical excision or stereotactic radiosurgery, chosen by the participant and their doctor) and willing to have either type of management.**

**If patients are suitable for both types of management and equally suitable and accepting of neurosurgery or stereotactic radiosurgery, the type of surgical management that would be allocated can be decided by randomisation.**

Importance of the wider team for CARE

The CARE study is very much a team effort and sites with ‘buy-in’ from lots of different clinical professionals are likely to recruit more participants and find the recruitment process easier. It can be helpful to understand what your colleagues think about the prospect of their patients being approached for CARE, given the wider team involvement needed for CARE.

“There will also be involvement from the neurologists and one of the specialist nurses. Every day we have a lot of interaction **with the whole team**”

**“[Colleague] has been helpful**, in trying to refer some patients to me that are coming in now.”


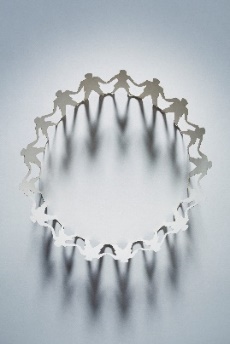


**Points to consider:**

- Who will be referring patients for discussions about CARE?
- Who at your site will be approaching patients about CARE?
- How can we work best together to screen and refer all suitable patients?

Engaging your MDTs with CARE

Different sites will have different referral pathways into CARE, with different MDTs identifying patients.

**It is helpful for a research or specialist nurse, or Associate PI (API) to attend the relevant MDTs to flag potentially eligible patients for a discussion about CARE**

Tips for screening

Due to the complexities of cavernomas, screening is usually performed by the site PI or the associate PI, with support from research nurses/coordinators. The online CRF should reflect where patients are up to in their CARE journey

Following their experiences of screening, the CARE team in Edinburgh developed an Excel spreadsheet to aid the screening process, which can be downloaded [here](https://www.ed.ac.uk/sites/default/files/atoms/files/care_screen_as_you_go_spreadsheet_v1.0_04_feb_2022.xlsx).

Where confirmation of diagnosis or eligibility is outstanding, you can save time by logging these patients on the Excel spreadsheet initially and transferring them to the CARE study database once details are confirmed.

**CARE QRI Collaborators List for PubMed**

| **Forename** | **Surname** |
| --- | --- |
| Conor | Mallucci |
| Philip M | White |
| Madeleine | Eriksson |
| Raza | Hayat |
| Elaine | Kinsella |
| Katherine | Lewis |
| Aileen R | Neilson |
| David CS | White |
| Julia | Boyd |
| Alastair | Bullen |
| Morag | Maclean |
| Andrew | Stoddart |
| Sandra | Phair |
| Helen | Evans |
| Jo | Noakes |
| Debra | Alexander |
| Catriona | Keerie |
| Christopher | Linsley |
| Garry | Milne |
| John | Norrie |
| Janet | Bunch |
| Kathryn | Douthwaite |
| Simon | Temple |
| James | Hogg |
| David | Scott |
| Pat | Spallone |
| Ian | Stuart |
| Joanna M | Wardlaw |
| Jeb | Palmer |
| Eleni | Sakka |
| Nitin | Mukerji |
| Emanuel | Cirstea |
| Susan | Davies |
| Venetia | Giannakaki |
| Ammar | Kadhim |
| Oliver | Kennion |
| Moidul | Islam |
| Lucie | Ferguson |
| Manjunath | Prasad |
| Andrew | Bacon |
| Emma | Richards |
| Jo | Howe |
| Christine | Kamara |
| Jonathan | Gardner |
| Madalina | Roman |
| Mary | Sikaonga |
| Julian | Cahill |
| Alex | Rossdeutsch |
| Varduhi | Cahill |
| Imron | Hamina |
| Kishor | Chaudhari |
| Mihai | Danciut |
| Emma | Clarkson |
| Anna | Bjornson |
| Diederik | Bulters |
| Ronneil | Digpal |
| Winnington | Ruiz |
| Mirriam | Taylor |
| Divina | Anyog |
| Katarzyna | Tluchowska |
| Jackson | Nolasco |
| Daniel | Brooks |
| Kleopatra | Angelopoulou |
| Bethany | Welch |
| Nicole | Broomes |
| Ioannis | Fouyas |
| Allan | MacRaild |
| Chandru | Kaliaperumal |
| Jessica | Teasdale |
| Michelle | Coakley |
| Paul | Brennan |
| Drahoslav | Sokol |
| Anthony | Wiggins |
| Mairi | MacDonald |
| Sarah | Risbridger |
| Pragnesh | Bhatt |
| Janice | Irvine |
| Sohail | Majeed |
| Sandra | Williams |
| John | Reid |
| Annika | Walch |
| Farah | Muir |
| Janneke | van Beijnum |
| Paul | Leach |
| Tom | Hughes |
| Milan | Makwana |
| Khalid | Hamandi |
| Dympna | McAleer |
| Belinda | Gunning |
| Daniel | Walsh |
| Oliver | Wroe Wright |
| Sabina | Patel |
| Nihal | Gurusinghe |
| Saba | Raza-Knight |
| Terri-Louise | Cromie |
| Allan | Brown |
| Sonia | Raj |
| Ruth | Pennington |
| Charlene | Campbell |
| Shakeelah | Patel |
| Francesca | Colombo |
| Mario | Teo |
| Jack | Wildman |
| Kerry | Smith |
| Elizabeth | Goff |
| Deanna | Stephens |
| Borislava | Borislavova |
| Ruth | Worner |
| Sandeep | Buddha |
| Philip | Clatworthy |
| Richard | Edwards |
| Evangeline | Clayton |
| Karen | Coy |
| Lisa | Tucker |
| Sandra | Dymond |
| Andrew | Mallick |
| Rebecca | Hodnett |
| Francesca | Spickett-Jones |
| Patrick | Grover |
| Azra | Banaras |
| Sifelani | Tshuma |
| William | Muirhead |
| Ciaran | Scott Hill |
| Rupal | Shah |
| Thomas | Doke |
| Rebecca | Hall |
| Sonny | Coskuner |
| Laura | Aslett |
| Raghu | Vindlacheruvu |
| Anthony | Ghosh |
| Teresa | Fitzpatrick |
| Lauren | Harris |
| Tom Hayton |  |
| Arlo | Whitehouse |
| Andrew | McDarby |
| Rebecca | Hancox |
| Claudia Kate | Auyeung |
| Ramesh | Nair |
| Rhys | Thomas |
| Heather | McLachlan |
| Athanasia | Kountourgioti |
| Guillelme | Orjales |
| Jan | Kruczynski |
| Sophie | Hunter |
| Niamh | Bohnacker |
| Rosette | Marimon |
| Lydia | Parker |
| Oishik | Raha |
| Puneet | Sharma |
| Christopher | Uff |
| Geetha | Boyapati |
| Marios | Papadopoulos |
| Siobhan | Kearney |
| Ravindran | Visagan |
| Ellaine | Bosetta |
| Hasan | Asif |
| Adel | Helmy |
| Liliana | Chapas |
| Silvia | Tarantino |
| Karen | Caldwell |
| Mathew | Guilfoyle |
| Smriti | Agarwal |
| Daniel | Brown |
| Sarah | Holland |
| Tamara | Tajsic |
| Clare | Fletcher |
| Aisha | Sebyatki |
| Shungu | Ushewokunze |
| Sarah | Ali |
| John | Preston |
| Carole | Chambers |
| Mohammed | Patel |
| Daniel | Holsgrove |
| Danielle | McLaughlan |
| Tracey | Marsden |
| Francesca | Colombo |
| Kathryn | Cawley |
| Hellen | Raffalli |
| Stephanie | Lee |
| Anil | Israni |
| Rachael | Dore |
| Taya | Anderson |
| Dawn | Hennigan |
| Shelley | Mayor |
| Samantha | Glover |
| Emmanuel | Chavredakis |
| Debbie | Brown |
| Giannis | Sokratous |
| John | Williamson |
| Cathy | Stoneley |
| Andrew | Brodbelt |
| Jibril Osman | Farah |
| Sarah | Illingworth |
| Anastasios Benjamin | Konteas |
| Deborah | Davies |
| Carol | Owen |
| Loretta | Kerr |

**Collaborators on delegation logs at hospital sites that recruited at least one participant to the CARE pilot trial**

Sites are listed in descending order of cumulative recruitment (quantified in brackets), indicating which people took the role of principal investigator (PI).

**James Cook University Hospital, Middlesbrough (13)** – Nitin Mukerji (PI), Emanuel Cirstea, Susan Davies, Venetia Giannakaki, Ammar Kadhim, Oliver Kennion, Md Moidul Islam, Lucie Ferguson and Manjunath Prasad; **Royal Hallamshire Hospital, Sheffield (12)** – Andrew Bacon (PI), Kirsty Harkness, Emma Richards, Jo Howe, Christine Kamara, Jonathan Gardner, Madalina Roman, Mary Sikaonga, Julian Cahill, Alex Rossdeutsch, Varduhi Cahill, Imron Hamina, Kishor Chaudhari; **Hull Royal Infirmary, Hull (8)** – Mihai Danciut (PI), Emma Clarkson and Anna Bjornson; **Wessex Neurological Centre, Southampton (7)** – Diederik Bulters (PI), Ronneil Digpal, Winnington Ruiz, Mirriam Taylor, Divina Anyog, Katarzyna Tluchowska, Jackson Nolasco, Daniel Brooks, Kleopatra Angelopoulou, Bethany Welch and Nicole Broomes; **Royal Infirmary of Edinburgh, Edinburgh (4)** – Ioannis Fouyas (PI), Allan MacRaild, Chandru Kaliaperumal, Jessica Teasdale, Michelle Coakley, James Loan, Rustam Al-Shahi Salman, Paul Brennan, Drahoslav Sokol, Anthony Wiggins, Mairi MacDonald and Sarah Risbridger; **Aberdeen Royal Infirmary, Aberdeen (4)** – Pragnesh Bhatt (PI), Janice Irvine, Sohail Majeed, Sandra Williams, John Reid, Annika Walch and Farah Muir; **University Hospital of Wales, Cardiff (3)** – Janneke van Beijnum (PI), Paul Leach, Tom Hughes, Milan Makwana, Khalid Hamandi, Dympna McAleer and Belinda Gunning; **King's College Hospital, London (3)** – Daniel Walsh (PI), Oliver Wroe Wright and Sabina Patel; **Royal Preston Hospital, Preston (2)** – Nihal Gurusinghe (PI), Saba Raza Knight, Terri-Louise Cromie, Allan Brown, Sonia Raj, Ruth Pennington, Charlene Campbell, Shakeelah Patel and Francesca Colombo; **Southmead Hospital, Bristol (2)** – Mario Teo (PI), Jack Wildman, Kerry Smith, Elizabeth Goff, Deanna Stephens, Borislava Borislavova, Ruth Worner, Sandeep Buddha and Philip Clatworthy; **Bristol Royal Hospital for Children, Bristol (2)** – Richard Edwards (PI), Evangeline Clayton, Karen Coy, Lisa Tucker, Sandra Dymond, Andrew Mallick, Rebecca Hodnett and Francesca Spickett-Jones; **National Hospital for Neurology and Neurosurgery, London (2)** – Patrick Grover (PI), Azra Banaras, Sifelani Tshuma, Neil Kitchen, William Muirhead, Ciaran Scott Hill, Rupal Shah, Thomas Doke, Rebecca Hall, Sonny Coskuner and Laura Aslett; **Essex Neurological Centre, Romford (1)** – Raghu Vindlacheruvu (PI), Anthony Ghosh, Teresa Fitzpatrick and Lauren Harris; **Queen Elizabeth Neuroscience Centre, Birmingham (1)** – Tom Hayton (PI), Arlo Whitehouse, Andrew McDarby, Rebecca Hancox, Claudia Kate Auyeung; **Charing Cross Hospital, London (1)** – Ramesh Nair (PI), Rhys Thomas, Heather McLachlan, Athanasia Kountourgioti, Guillelme Orjales, Jan Kruczynski, Sophie Hunter, Niamh Bohnacker, Rosette Marimon, Lydia Parker, Oishik Raha and Puneet Sharma; **Royal London Hospital, London (1)** – Christopher Uff (PI) and Geetha Boyapati; **St. George's Hospital, London (1)** – Marios Papadopoulos (PI), Siobhan Kearney, Ravindran Visagan, Ellaine Bosetta and Hasan Asif; **Addenbrooke's Hospital, Cambridge (1)** – Adel Helmy (PI), Liliana Chapas, Silvia Tarantino, Karen Caldwell, Mathew Guilfoyle, Smriti Agarwal, Daniel Brown, Sarah Holland, Tamara Tajsic, Clare Fletcher and Aisha Sebyatki; **Sheffield Children's Hospital, Sheffield (1)** – Shungu Ushewokunze (PI), Sarah Ali, John Preston, Carole Chambers and Mohammed Patel; **Salford Royal NHS Foundation Trust, Salford (1)** – Dan Holsgrove (PI), Danielle McLaughlan, Tracey Marsden, Francesca Colombo, Kathryn Cawley, Hellen Raffalli, Saba Raza-Knight, Stephanie Lee; **Alder Hey Children’s Hospital, Liverpool (1)** – Conor Mallucci, Anil Israni, Rachael Dore, Taya Anderson, Dawn Hennigan, Shelley Mayor and Samantha Glover; **The** **Walton Centre, Liverpool (1)** – Emmanuel Chavredakis (PI), Debbie Brown, Giannis Sokratous, John Williamson, Cathy Stoneley, Andrew Brodbelt, Jibril Osman Farah, Sarah Illingworth, Anastasios Benjamin Konteas, Deborah Davies, Carol Owen and Loretta Kerr.
