# Supplementary material for: Addressing barriers and identifying facilitators to support informed consent and recruitment in the Cavernous malformations A Randomised Effectiveness (CARE) pilot phase trial: insights from the integrated QuinteT recruitment intervention (QRI)
Source: eClinicalMedicine. 2024 Apr 18;71:102557. doi: 10.1016/j.eclinm.2024.102557 (PMC11133797; doi:10.1016/j.eclinm.2024.102557)
Supplement: CARE Protocol V2.0 (22Mar2021).pdf [file mmc2.pdf]

Academic and Clinical Central Office for Research and Development

Study Protocol

Cavernomas A Randomised Effectiveness (CARE) pilot trial, to address the effectiveness of active treatment (with neurosurgery or stereotactic radiosurgery) versus conservative management in people with symptomatic brain cavernoma

Co-sponsors	The University of Edinburgh and/or Lothian Health Board<br>ACCORD<br>The Queen's Medical Research Institute<br>47 Little France Crescent<br>Edinburgh<br>EH16 4TJ
Trial Management Group (listed alphabetically by surname after the chief investigator)	Prof Rustam Al-Shahi Salman (chief investigator)<br>Mr Neil Kitchen (co-chief investigator)<br>Dr Vijeya Ganesan<br>Dr Peter Hall<br>Dr Kirsty Harkness<br>Prof Peter Hutchinson<br>Dr Elaine Kinsella<br>Prof Steff Lewis<br>Mr Jamie Loan<br>Prof Conor Mallucci<br>Mr Matthias Radatz<br>Mr Andy Stoddart<br>Ms Carole Turner<br>Dr Julia Wade<br>Mr David White<br>Prof Phil White
Funder	National Institute for Health Research Health Technology Assessment Programme
Funding Reference Number	NIHR128694

Chief Investigator	Prof Rustam Al-Shahi Salman
Sponsor number	AC20171
REC Number	21/YH/0046
Project registration	To be confirmed
Version Number and Date	V2.0 (22Mar 2021)

CONTENTS

SCIENTIFIC ABSTRACT 9
PLAIN ENGLISH SUMMARY 10
1 INTRODUCTION 11
1.1 BACKGROUND 11
1.1.1 What are brain cavernomas? 11
1.1.2 What treatments are available in standard clinical practice for brain cavernoma? 11
1.1.3 What evidence supports medical management vs. medical and surgical management of brain cavernoma? 12
1.1.4 Observational studies comparing medical management with medical and surgical management for brain cavernoma 14
1.1.5 Summary of procedures, benefits and risks with medical management or medical and surgical management for brain cavernoma 15
1.2 RATIONALE FOR STUDY 16
1.2.1 The therapeutic dilemma 16
1.2.2 Understanding recruitment barriers with a QuinteT recruitment intervention (QRI) 16
1.2.3 This feasibility study and pilot trial will inform the feasibility of a definitive main phase trial 17
1.2.4 Patient, carer and public involvement (PCPI) 17
2 STUDY OBJECTIVES 18
2.1 OBJECTIVES 18
2.1.1 Primary objective 18
2.1.2 Secondary objectives 18
2.2 OUTCOMES 18
2.2.1 Primary outcome 18
2.2.2 Primary clinical outcome 19
2.2.3 Secondary clinical outcomes 20
2.2.4 Feasibility metrics proposed to the funder 20
3 STUDY DESIGN 20
3.1 TRIAL PROFILE 21
3.1.1 QuinteT recruitment intervention 22
4 STUDY POPULATION 23
4.1 NUMBER OF PARTICIPANTS 23
4.2 INCLUSION CRITERIA 23
4.3 EXCLUSION CRITERIA 24
4.4 CO-ENROLMENT 24
5 PARTICIPANT SELECTION AND ENROLMENT 24
5.1 IDENTIFYING AND SCREENING PARTICIPANTS 24
5.2 APPROACHING AND CONSENTING PARTICIPANTS 25
5.2.1 Consent to the QRI 27
5.2.2 Consent to participate in the CARE pilot trial 27
5.2.3 Consent to be contacted for an interview exploring reasons for declining participation 29
5.3 SCREENING AND ENROLMENT LOGS 29
5.4 RANDOMISATION 30

5.4.1 Randomisation procedures 30
5.4.2 Treatment allocation 30
5.4.3 Blinding (masking) 30
5.5 WITHDRAWAL OF PARTICIPANTS 30
5.5.1 Loss of mental capacity in adult participants in England and Wales 31
5.5.2 Loss of mental capacity in adult participants in Scotland 31
5.5.3 Loss of mental capacity in adult participants in Northern Ireland 31
5.5.4 Loss of mental capacity in adult participants in the Republic of Ireland 32
6 COMPARATOR 32
7 INTERVENTION 32
7.1 Neurosurgical excision 33
7.2 Stereotactic radiosurgery 33
8 STUDY ASSESSMENTS 33
8.1 STUDY ASSESSMENTS 33
8.1.1 Table of assessments 34
8.1.2 Screening 35
8.1.3 Informed consent 35
8.1.4 Baseline visit 35
8.1.5 Three-month adherence check 36
8.1.6 Six-month local follow-up visit 36
8.1.7 Six-monthly central follow-up visit 37
8.1.8 Patient Interviews 37
8.2 LONG TERM FOLLOW UP 38
8.3 BRAIN MAGNETIC RESONANCE IMAGING 38
8.4 OUTCOME EVENT ADJUDICATION 38
8.5 DNA SAMPLE STORAGE AND ANALYSIS 38
9 DATA COLLECTION 39
9.1 SOURCE DATA DOCUMENTATION 39
9.2 CASE REPORT FORMS 39
9.3 STUDY DATABASE 39
9.4 QRI DATA COLLECTION 39
9.4.1 Screening log data 39
9.4.2 Recordings of recruitment conversations 39
9.4.3 Patient and staff interviews 40
9.4.4 Meetings 40
9.4.5 Trial documentation 40
10 DATA MANAGEMENT AND TRANSFER 41
10.1 PERSONAL DATA 41
10.2 BRAIN MRI SCANS 41
10.3 QUINTET RECRUITMENT INTERVENTION 41
10.3.1 Recordings of recruitment conversations 41
10.3.2 Interviews 42
10.3.3 QRI documentation 42
10.4 DATA CONTROLLER 42
10.5 DATA BREACHES 43
11 STATISTICS AND DATA ANALYSIS 43
11.1 SAMPLE SIZE CALCULATION 43
11.2 PROPOSED STATISTICAL ANALYSES 43

11.3 QUINTET RECRUITMENT INTERVENTION DATA ANALYSIS 44
11.3.1 Screening and enrolment logs 44
11.3.2 Recordings of recruitment conversations and interviews 44
12 HEALTH ECONOMICS AND DATA ANALYSIS 44
13 ADVERSE EVENTS 45
13.1 DEFINITIONS 45
13.2 IDENTIFYING SAEs 46
13.3 RECORDING SAEs 46
13.3.1 Pre-existing medical conditions 46
13.3.2 Worsening of the underlying condition during the trial 46
13.4 ASSESSMENT OF AEs AND SAEs 46
13.4.1 Assessment of Seriousness 47
13.4.2 Assessment of Causality 47
13.4.3 Assessment of Expectedness 47
13.4.4 Assessment of Severity 47
13.5 REPORTING OF SAEs 48
14 PREGNANCY 48
15 OVERSIGHT ARRANGEMENTS 48
15.1 TRIAL MANAGEMENT GROUP 48
15.2 TRIAL STEERING COMMITTEE 48
15.3 DATA MONITORING COMMITTEE 49
15.4 PATIENT ADVISORY GROUP 49
15.5 INSPECTION OF RECORDS 49
15.6 STUDY MONITORING AND AUDIT 49
16 GOOD CLINICAL PRACTICE 49
16.1 ETHICAL CONDUCT 49
16.2 INVESTIGATOR RESPONSIBILITIES 50
16.2.1 Informed Consent 50
16.2.2 Study Site Staff 50
16.2.3 Data Recording 50
16.2.4 Investigator Documentation 50
16.2.5 Training 50
16.2.6 Confidentiality 51
16.2.7 Data Protection 51
STUDY CONDUCT RESPONSIBILITIES 52
16.3 PROTOCOL AMENDMENTS 52
16.4 MANAGEMENT OF PROTOCOL NON-COMPLIANCE 52
16.5 SERIOUS BREACH REQUIREMENTS 52
16.6 STUDY RECORD RETENTION 53
16.7 END OF TRIAL 53
16.8 CONTINUATION OF TREATMENT FOLLOWING THE END OF STUDY 53
16.9 INSURANCE AND INDEMNITY 53
17 REPORTING, PUBLICATIONS AND NOTIFICATION OF RESULTS 54
17.1 AUTHORSHIP POLICY AND REPORTING 54
17.2 PUBLICATION AND DISSEMINATION 54
17.3 DATA SHARING 54
18 TRIAL TIMELINE 56

19 PROTOCOL VERSION CONTROL HISTORY 57
19.1 Version 1.0 (29Jan2021) 57
19.2 Version 2.0 (22Mar2021) 57
20 REFERENCES 58

LIST OF ABBREVIATIONS

**95% CI**	95% confidence interval
**ACCORD**	Academic and Clinical Central Office for Research & Development - Joint office for The University of Edinburgh and Lothian Health Board
**CARE**	Cavernomas A Randomised Effectiveness trial
**CAUK**	Cavernoma Alliance UK
**CI**	Chief Investigator
**CRF**	Case Report Form
**DMC**	Data Monitoring Committee
**DWI**	Diffusion-Weighted Imaging
**eCRF**	Electronic Case Report Form
**ECTU**	Edinburgh Clinical Trials Unit
**FLAIR**	Fluid Attenuated Inversion Recovery
**GCP**	Good Clinical Practice
**ICF**	Informed Consent Form
**ICH GCP**	International Conference on Harmonisation for Good Clinical Practice
**MRI**	Magnetic Resonance Imaging
**PAG**	Patient, carer and public involvement Advisory Group
**PI**	Principal Investigator
**PIL**	Patient Information Leaflet
**QA**	Quality Assurance
**QRI**	QuinteT Recruitment Intervention
**QuinteT**	Qualitative Research Integrated within Trials
**RaDAR**	Rare Disease Ascertainment and Recruitment
**REC**	Research Ethics Committee
**RCT**	Randomised controlled trial
**SAIVMs**	Scottish Audit of Intracranial Vascular Malformations
**SOP**	Standard Operating Procedure
**TCC**	Trial Coordinating Centre

**TMG**	Trial Management Group
**TSC**	Trial Steering Committee

SCIENTIFIC ABSTRACT

This is a pilot randomised controlled trial (RCT) to assess the feasibility of conducting a definitive main phase RCT to address the research question commissioned by the NIHR HTA, "How effective is active treatment (with neurosurgery or stereotactic radiosurgery) versus conservative management in people with symptomatic brain cavernoma?" The terms ‘conservative management’ and ‘active treatment were used in the commission, but throughout this protocol we will refer to ‘conservative management’ as ‘medical management’ and ‘active treatment’ as ‘medical and surgical management’. We will assess: collaborator engagement; proportions of screened patients who are eligible, approached, consented, or randomised; barriers to recruitment; RCT procedure implementation; adherence; data completeness; outcome event rates; and generalisability.

At least 160 people with brain cavernomas are newly diagnosed after symptoms due to stroke or epilepsy in the UK each year. A James Lind Alliance Priority Setting Partnership found that the top research priority for cavernoma was, “Does treatment (with neurosurgery or stereotactic radiosurgery) or no treatment improve outcome for people diagnosed with a cavernoma?”. A RCT is required to answer this question, but systematic reviews and trial register searches have not revealed any such RCTs.

The Cavernomas A Randomised Effectiveness (CARE) pilot trial aims to:

1. Engage a collaboration of specialists and patient advocacy groups in the UK and Ireland.
2. Establish a pilot RCT, with an embedded qualitative study to understand the anticipated recruitment processes and address any barriers.
3. Assess the feasibility of performing a definitive main phase of the RCT.

The CARE pilot trial will include:

I. A pilot phase parallel group RCT for patients with symptomatic brain cavernoma, comparing medical management versus medical and surgical management (with neurosurgery or stereotactic radiosurgery), with randomisation stratified by preferred type of surgical management. Collaborators will keep screening logs to capture characteristics of patients screened, eligible, approached, consented and randomised. This prospective randomised open blinded end-point RCT will recruit ~60 participants.

II. A QuinteT recruitment intervention (QRI) will evaluate screening logs and incorporate qualitative research to understand recruitment processes and barriers and identify actions to address barriers.

We will use (I) and (II) to estimate the feasibility and generalisability of a definitive main phase of the CARE RCT by extending the UK collaboration to other patient support organisations and clinical communities elsewhere in the world.

PLAIN ENGLISH SUMMARY

A cavernoma is a cluster of blood vessels that form blood-filled 'caverns' in the brain that look like a raspberry. Cavernomas can bleed into the brain and cause a stroke. Cavernomas can also cause a seizure or epilepsy. About 160 people in the UK each year are diagnosed with a cavernoma that has caused symptoms. Stroke and seizure may lead to disability, handicap and occasionally death. In standard practice in the UK, most people with cavernomas have medical management (which may involve scans, drugs, or rehabilitation) to manage these symptoms. About one fifth also have 'surgical management' with either brain surgery to remove a cavernoma or stereotactic radiosurgery to stabilise it with radiation. Surgical management can cause death, disability, and handicap.

The pros and cons of medical management versus medical and surgical management are finely balanced. The most reliable way of finding out which management is best is to do a randomised trial, in which suitable patients are allocated to medical management or medical and surgical management at random. This has never been done with cavernomas, and this was the top priority identified by a Priority Setting Partnership for cavernoma.

The NIHR wants research to be done to find out whether enough patients can be found for a randomised trial comparing 'medical management with 'medical and surgical management' of symptomatic cavernomas. We need to know this because cavernomas are rare and we do not know whether patients and doctors will take part.

In three years, we will:

(1) Create a network of specialists to do this study. We will include the UK and Ireland patient support organisations for people with cavernoma (Cavernoma Alliance UK - CAUK) and doctors representing the relevant specialties at all the major hospitals specialising in decisions about cavernoma treatment in the UK and Ireland.

(2) Invite newly diagnosed patients to join a pilot phase of a randomised controlled trial. Of 190 people diagnosed with brain cavernoma in 18 months, we estimate that 60 of them will enrol in the randomised trial. We will study why some patients take part in the randomised trial and others don't. We will use this information to change the methods of the trial if recruitment to the randomised trial goes slowly.

(3) Estimate whether enough patients can be found for a full-scale randomised trial to be done to find out whether medical management or medical and surgical management of symptomatic brain cavernomas is best.

We involved people with cavernoma, carers, and representatives of CAUK with patients and carers on 6 July 2019: all approved the design of the project and the extent of patient and public involvement. The focus group wanted the trials to be as inclusive of patients as possible. The focus group recognised how the project would benefit from them contributing their 'lived experience' of brain cavernoma.

People with cavernoma, carers, and representatives of CAUK will also keep an eye on the research by forming an advisory group and meeting regularly to discuss the research. Two representatives of this group will join and advise the steering committee.

We will publish our findings in medical journals. We will work with CAUK to produce a plain English summary and circulate it to patients via newsletters, email, the web, and social media.

1 INTRODUCTION

1.1 BACKGROUND

1.1.1 What are brain cavernomas?

Cerebral cavernous malformations or ‘cavernomas’ are intracranial vascular malformations that are diagnosed using histopathological examination or magnetic resonance imaging (MRI). Although most cavernomas are solitary and sporadic, around one-fifth are multiple with autosomal dominant inheritance due to mutations in three genes (1), so there are implications for relatives as well.

Large brain MRI cohorts have shown that the asymptomatic prevalence of brain cavernomas is 0.16%, currently affecting ~106,000 people in the UK (2). Some of these people present to medical attention with symptoms such as epileptic seizures or stroke due to either intracranial haemorrhage or ‘focal neurological deficits’ anatomically related to the cavernoma that do not appear to be due to haemorrhage (3). The incidence of symptomatic cavernoma in the UK was 0.24 per 100,000 per year at the turn of the millennium (4), so approximately 160 people are newly-diagnosed with symptomatic cavernoma in the UK annually. The impact of cavernoma is disproportionately high in comparison to their frequency, because they are usually diagnosed in children and young adults of working age (4).

People with cavernoma face a considerable risk of recurrent stroke due to intracranial haemorrhage, which is reliably known over five years after diagnosis (5), but is likely to continue for their lifetime. The 5-year risk of intracranial haemorrhage ranges from ~3.8% for people with non-brainstem cavernoma who have presented without a stroke to ~30.8% for people with brainstem cavernoma who have presented with stroke due to intracranial haemorrhage or focal neurological deficit.

People with cavernoma who present with an epileptic seizure almost inevitably develop epilepsy within one year, and only half of people with cavernoma-related epilepsy achieve two-year seizure-freedom (6).

These persistent symptoms also cause economic consequences for people with cavernoma, carers, the NHS, social services, and lost productivity in the UK workforce (7).

1.1.2 What treatments are available in standard clinical practice for brain cavernoma?

‘Medical management’ constitutes standard medical care alone (e.g. prevention of epileptic seizures with anti-epileptic drugs, and rehabilitation of neurological deficits, according to UK guidelines (8)). This is the most frequently used management plan for people with brain cavernoma in the UK (9).

Surgical management of brain cavernoma with neurosurgical excision or stereotactic radiosurgery is used in standard clinical practice for some patients to try to prevent recurrent epileptic seizures and stroke due to intracranial haemorrhage or non-haemorrhagic focal neurological deficit, which can result in death, disability,

handicap, and psychological consequences for patients and carers (10). Surgical management is given in addition to medical management in standard clinical practice, as described above, so throughout this protocol we will refer to this as 'medical and surgical management' for clarity.

Medical and surgical management in the CARE pilot trial involves health technologies that are available in standard clinical practice in the UK and Republic of Ireland; these are either neurosurgical excision (performed by neurosurgeons at 37 regional adult or paediatric neuroscience centres) or stereotactic radiosurgery (using Gamma Knife performed at the National Centre for Stereotactic Radiosurgery in Sheffield or the Queen Square Radiosurgery Centre). Neurosurgical excision is the most frequently-used form of surgical treatment for brain cavernoma in the UK, but it involves a craniotomy and the risk of complications is much higher for some cavernomas deep within the brain or brainstem that cannot be accessed without traversing brain tissue with important functions. Stereotactic radiosurgery (using Gamma Knife) is non-invasive and may be used because neurosurgery is too risky or a patient wants a non-invasive treatment. There are some emerging technologies for the surgical treatment of brain cavernomas, including minimally invasive therapeutic approaches for brain cavernoma such as magnetic resonance thermography-guided laser interstitial thermal therapy, or stereotactic laser ablation (11). Although medical and surgical management in the CARE pilot trial will continue to be neurosurgical excision or Gamma Knife stereotactic radiosurgery plus medical management, we will collect details of each type of surgical treatment used after randomisation to allow us to quantify the use of emerging technologies.

Medical and surgical management can have complications that can be fatal or disabling (9; 12; 13), and there are few reliable data about the benefits and risks of medical management versus medical and surgical management (8; 14; 15), so most patients have medical management (9).

Although drugs like propranolol, antiplatelet agents, anticoagulant agents and statins are not licensed for the treatment of brain cavernoma, some clinicians may use them off-label for patients who are unsuitable for medical and surgical management because these drugs may have disease-modifying effects (16).

1.1.3 What evidence supports medical management vs. medical and surgical management of brain cavernoma?

A search of ClinicalTrials.gov trial register on 17 November 2020 using the terms, "cavernoma OR cavernous angioma OR cavernous malformation" revealed five RCTs of drug therapies for brain cavernoma, but no completed, ongoing, or planned RCTs comparing medical management with medical and surgical management.

In several systematic reviews of observational cohort studies comparing medical management to medical and surgical management of brain cavernoma, or one form of surgical management to another, there were no studies at low risk of bias that demonstrated sufficiently "dramatic" associations between medical management versus medical and surgical management of brain cavernoma and clinical outcomes that would make a RCT unnecessary (14; 17).

We performed or updated (to 2018-2019) several systematic reviews and meta-analyses including:

i. observational cohort studies that compared medical and surgical management involving stereotactic radiosurgery or neurosurgery against medical management in a concurrent or historical control group and reported clinical outcome (14; 18)

ii. observational cohort studies without comparison groups reporting clinical outcomes after either medical management (5), neurosurgery (9; 19), or stereotactic radiosurgery (18; 19); and
iii. decision analysis comparing all management strategies using a Markov model with a time horizon of five years (20)

The best available evidence from observational studies comparing medical management with medical and surgical management is summarised in a table (see 1.1.4 below) and in more detail in the following paragraphs.

1.1.3.1 <u>Neurosurgery versus medical management</u>

There are seven observational cohort studies that compare neurosurgery and medical management (9; 21; 22; 23; 24; 25; 26). The best available comparative data on an entire incident brain cavernoma population found neurosurgery to be associated with harm over five years (hazard ratios 2.2-3.6) (9)), although other comparative studies restricted to brainstem/deep cavernomas have suggested both harm (risk ratios 1.9-7.8) and benefit (risk ratios 0.5-0.6) on the risk of intracranial haemorrhage over 4-6 years (21; 22; 23; 24), but the long-term difference in risk is unknown and might favour neurosurgery.

1.1.3.2 <u>Stereotactic radiosurgery versus medical management</u>

In the only observational cohort study comparing stereotactic radiosurgery with medical management at one hospital in Korea (27) (see table below), stereotactic radiosurgery might have been harmful, but the risk ratio was incalculable because of the paucity of outcomes. Indirect comparisons imply that stereotactic radiosurgery might be superior to medical management over five years. In a systematic review and meta-analysis of 30 cohort studies of patients undergoing stereotactic radiosurgery for brain cavernoma (median 61% of whom had brainstem cavernoma and median 91% of whom had presented with intracranial haemorrhage), during a median follow-up of 48 (IQR 35-62) months after stereotactic radiosurgery, the annual incidence of the composite of death, intracranial haemorrhage or focal neurological deficit was 3.6% (95% CI 3.17-4.16) (18). Using these data to estimate the five-year risk (16.9%) after stereotactic radiosurgery and comparing the risk indirectly to the cumulative 5-year risks of intracranial haemorrhage with medical management that range from ~18% to ~31% for comparable patient groups (5), suggests that stereotactic radiosurgery might be superior to medical management over five years. A systematic review of stereotactic radiosurgery restricted to brainstem cavernoma suggested that treatment was beneficial by comparing intracranial haemorrhage risks before and after treatment (13), but their findings are unreliable because they may simply reflect the untreated clinical course of brain cavernoma in which intracranial haemorrhage risk declines over time (5).

Our summary of the procedures, benefits and risks for patients and carers is also summarised in a table (see1.1.5 1.1.5 below).

1.1.4 Observational studies comparing medical management with medical and surgical management for brain cavernoma.

Study	Population	Intervention	Comparator	Outcomes / Time	Medical vs. medical and surgical management absolute &/or relative risk(s) of ICH
**Neurosurgery vs. medical management**					
Brain cavernomas in any location					
Moultrie *et al.* 2014 (9)	134 adults (40 had caused ICH/FND)	Surgery (n=25)	Medical management (n=109)	Functional outcome (at least 2 successive ratings of >1 on the mRS), or new ICH/FND during 5y follow-up	Functional outcome: 13/25 vs. 40/109 (**aHR 2.2, 95% CI 1.1–4.3**) ICH/FND: 8/25 vs. 17/109 (**aHR 3.6, 95% CI 1.3–10.0**)
Kida *et al.* 2015 (25)	78 adults (53 had caused ICH)	Surgery (n=29)	Medical management (n=49)	ICH during 3.8-4.6y follow-up	2/29 vs. 16/49 (**RR 0.6, 95% CI 0.1–2.6**)
Brainstem/deep cavernomas					
Esposito *et al.* 2003 (20)	30 adults (26 had caused ICH/FND)	Surgery (n=13)	Medical management (n=17)	ICH/FND over average 3.9y	6/13 vs. 1/17 (**RR 7.8, 95% CI 1.1–57.4**)
Mathiesen *et al.* 2003 (21)	68 adults (48 had caused ICH/FND)	Surgery (n=29)	Medical management (n=34)	ICH over average 4.6y	4/29 vs. 8/34 (**RR 0.6, 95% CI 0.2–1.7**)
Tarnaris *et al.* 2008 (22)	21 adults (17 had caused ICH/FND)	Surgery (n=6)	Medical management (n=15)	ICH over average 6.5y	3/6 vs. 4/15 (**RR 1.9, 95% CI 0.6–6.0**)
Huang *et al.* 2010 (23)	30 adults (30 had caused ICH/FND)	Surgery (n=22)	Medical management (n=8)	"Deterioration" over average 4y	3/22 vs. 2/8 (**RR 0.5, 95% CI 0.1–2.7**)
Brain cavernomas not in brainstem/deep locations					
Kivelev *et al.* 2009 (24)	33 adults (15 had caused ICH)	Surgery (n=18)	Medical management (n=15)	ICH over average 7.7y	0/18 vs. 4/15 (RR incalculable)
**Stereotactic radiosurgery vs. medical management**					
Yoon *et al.* 1998 (26)	41 adults with cavernomas in any location (20 had caused ICH/FND)	Gamma Knife stereotactic radiosurgery (n=22)	Medical management (n=19)	ICH, adverse radiation effects (ARE) over 2-3.5y	ICH: 2/22 vs. 0/19 (RR incalculable) ARE 5/22 vs. 0/19 (RR incalculable)

aHR = adjusted hazard ratio; ARE = adverse radiation effects; FND = focal neurological deficit; ICH = intracranial haemorrhage; mRS = modified Rankin Scale; RR = risk ratio (estimated from aggregate data).

1.1.5 Summary of procedures, benefits and risks with medical management or medical and surgical management for brain cavernoma

	Medical management	Medical and surgical management	
		Neurosurgery	Stereotactic radiosurgery
**What may be involved?**	• Treat symptoms<br>• Prevent seizures<br>• Rehabilitation<br>• Brain scan	• Treat symptoms<br>• Prevent seizures<br>• Rehabilitation<br>• Brain scan	• Treat symptoms<br>• Prevent seizures<br>• Rehabilitation<br>• Brain scan
		• Hospital admission for days<br>• General anaesthetic<br>• Opening in the skull<br>• Operation to remove cavernoma<br>• Follow-up brain scan<br>• Must not drive for 6 months	• Hospital attendance for a day<br>• Anaesthetic not needed<br>• Head fixed in a temporary frame<br>• Focussed radiation given once<br>• Follow-up brain scans
**What are the possible benefits?**	• Bleed/stroke risk reduces as time passes<br>• Avoids risks of neurosurgery or radiosurgery	• Risk of bleed/stroke lower if cavernoma removed<br>• Less worry about symptoms returning	• Risk of bleed/stroke may be lower if cavernoma stabilised, but these benefits are uncertain<br>• Less worry about symptoms returning
**What are the possible risks?**	• Future bleed/stroke due to cavernoma<br>○ Can be mild<br>○ May be disabling<br>○ Rarely be fatal<br>○ Risk higher for cavernoma in brainstem<br>• Epileptic seizures, which may be difficult to control<br>• Cavernoma remains in the brain, so the risks of stroke and seizure may never go away<br>• Worry about symptoms returning	• Bleed/stroke due to neurosurgery<br>○ Can be mild<br>○ May be disabling<br>○ Rarely be fatal<br>○ Risk higher for cavernoma in brainstem<br>• Epileptic seizures may not go away<br>• Complications of treatment (e.g. infection or damage to brain around the cavernoma)<br>• Cavernoma may come back	• Bleed/stroke despite radiosurgery<br>○ Can be mild<br>○ May be disabling<br>○ Rarely be fatal<br>○ Risk higher for cavernoma in brainstem<br>• Epileptic seizures may not go away<br>• Complications of treatment (e.g. damage to brain around the cavernoma)<br>• Cavernoma not removed

1.2 RATIONALE FOR STUDY

1.2.1 The therapeutic dilemma

The shortage of high-quality evidence to inform the management of patients with brain cavernomas has prevented clinical guidelines in the UK and USA from making strong recommendations about whether to use medical management or medical and surgical management for brain cavernomas (8; 15). These uncertainties were confirmed by patients and carers in a James Lind Alliance Priority Setting Partnership in the UK, which found that the top research priority for cavernoma was, “Does treatment (with neurosurgery or stereotactic radiosurgery) or no treatment improve outcome for people diagnosed with brain or spine cavernoma?” (28).

Therefore, in 2018 the NIHR HTA commissioned research to address the question, “How effective is treatment (with neurosurgery or stereotactic radiosurgery) versus conservative management in people with symptomatic brain cavernoma?” The NIHR’s commissioning brief reported that feedback from experts suggested that a randomised controlled trial (RCT) with at least 10 years of follow-up would be needed to better guide clinical care and that it would be necessary to conduct a multinational trial in countries with similar healthcare settings to the UK to ensure sufficient numbers for a robust trial.

1.2.2 Understanding recruitment barriers with a QuinteT recruitment intervention (QRI)

Resolving this therapeutic dilemma is likely to be challenging because of the low incidence of symptomatic brain cavernoma despite a high prevalence, because the availability of surgical management varies in everyday clinical practice (8; 15), and because accumulated expertise in specialist centres has guided clinical practice hitherto despite the lack of high quality evidence (29). Recruitment to the CARE pilot trial is likely to remain challenging given the history of RCTs comparing medical management versus medical and surgical management of intracranial vascular malformations with invasive procedures (30; 31). The reasons for poor recruitment to such trials have not been studied, so qualitative research is needed to investigate the potential barriers to recruitment and optimise recruitment processes in the CARE pilot trial. Many RCTs experience recruitment challenges due to difficulties that recruiters have in explaining concepts like uncertainty, equipoise and randomisation (32). Discussions with members of our collaboration during the development of this proposal have raised concerns about clinical equipoise amongst neurosurgeons, partly due to treatment preferences according to the anatomical location of the brain cavernoma, concerns about exposing children to radiation, scepticism about the effects of stereotactic radiosurgery, and the availability of stereotactic radiosurgery in the NHS for brain cavernoma at only two sites in the UK (although patients may be referred from any hospital) (29). Also, patients may have treatment preferences (e.g. for less invasive procedures), and patient/family preferences may affect RCTs involving children in particular (33).

An integrated QRI aims to understand recruitment barriers (e.g. related to selection of patients during screening and recruitment processes, or equipoise, etc.) and optimise informed consent and recruitment processes in the CARE pilot trial (32; 33; 34). Embedding a QRI allows the identification and understanding of generic and trial-specific recruitment challenges (35; 36; 37), and enables the development of tailored plans to address these issues. A QRI (38) has been integrated into over 30

RCTs, including trials comparing surgery and medical management (39) and there is observational evidence of the benefits associated with a QRI in at least five RCTs (40).

1.2.3 This feasibility study and pilot trial will inform the feasibility of a definitive main phase trial

The NIHR HTA commissioned a UK feasibility study and pilot phase RCT to demonstrate the ability to recruit enough patients to answer the research questions and sufficient numbers in the UK such that the trial results would be applicable to the NHS. The CARE pilot trial was funded by this NIHR HTA commissioned call. A decision about whether to proceed a definitive main phase trial will be made in light of the results of the CARE pilot trial.

1.2.4 Patient, carer and public involvement (PCPI)

Between August 2014 and November 2015 we worked with people with cavernoma, carers, and representatives of the patient support organisation Cavernoma Alliance UK (CAUK) on the Steering Group of the James Lind Alliance Priority Setting Partnership that identified and prioritised the topic of this application as the top priority for further research into cavernoma. Since November 2015, individuals in the Steering Group of the James Lind Alliance Priority Setting Partnership – including patients and carers – were involved in reviewing the commissioning brief for the NIHR HTA commissioned call for research. In May-June 2016, we worked with CAUK to gather the views of patients and carers who are members of the organisation, about research to address this top priority for further research into cavernoma. We consulted 731 CAUK members affected by cavernoma or parents/guardians of affected children, by emailing them a link to a web-based survey describing the CARE trial. 70% of respondents had not received surgical management for a cavernoma and a minority (28%) of these respondents indicated that they would not participate in the RCTs proposed. Between December 2018 and June 2019, we consulted representatives and members of CAUK, including patients with the condition, who have reviewed and shaped the design of the CARE pilot trial. In July 2019, all members of CAUK were invited by the Chief Executive of the organisation to participate in a focus group on 6th July. Four carers, six patients, the Chief Executive Officer of CAUK and the Chief Investigator (CI) attended the meeting. This focus group of patients, carers, and family members considered the overall design of this project. The main themes of the discussion were: (1) The group recognised that, "many people have had to make difficult decisions without the information they need" and that in addressing this "difficult dilemma", their involvement could improve participation by contributing their 'lived experience' of brain cavernoma to the clinical experience of the co-applicants and the planned qualitative research; (2) The group approved the extent of the patient and public involvement that is planned; (3) The group wanted the CARE pilot trial to be as inclusive of patients as possible. In particular, they wanted the CARE pilot trial to include patients who have: (a) first presented with symptoms or been diagnosed some time ago, (b) multiple cavernomas (one of which might have been treated), and (c) partially treated cavernoma (for whom there is uncertainty about further treatment); (4) All participants approved the project's design. In particular, they approved a choice of the safest treatment according to cavernoma location, using the "wealth of experience" of the clinical community in the UK, permitting patient preferences, and allowing treatment if needed during follow-up; (5) The group accepted that participants would receive standard care; (6) The group asked not only that the project should include a diverse sample of patients with brain cavernoma, but

also that the analyses should account for this diversity (e.g. age, time since symptoms, single vs. multiple cavernoma, and genetic mutations).

2 STUDY OBJECTIVES

2.1 OBJECTIVES

2.1.1 Primary objective

Assess the feasibility of performing a definitive main phase of a RCT comparing medical management to medical and surgical management (with neurosurgery or stereotactic radiosurgery) for improving outcome for people with symptomatic brain cavernoma.

2.1.2 Secondary objectives

- Set up a collaboration of the patient advocacy organisations for cavernoma in the UK and Ireland and representatives of clinical neurology, neurosurgery, and stereotactic radiosurgery at neuroscience centres throughout the UK and Ireland.
- Evaluate screening logs and conduct qualitative research with patients and clinicians to understand recruitment processes and barriers, as well as actions to address any barriers, as part of a QuinteT recruitment intervention (QRI) to optimise informed consent and recruitment.
- Conduct the CARE pilot trial for approximately 60 patients with symptomatic brain cavernoma, comparing medical management of the brain cavernoma versus medical and surgical management (neurosurgery or Gamma Knife stereotactic radiosurgery) for improving outcome.

2.2 OUTCOMES

2.2.1 Primary outcome

We will estimate these measures of feasibility to inform the extent to which international cooperation would be needed to recruit an adequate sample size in a CARE definitive main phase RCT, and what proportion of participants might be recruited from the UK during the study:

1. What proportion of the collaborating centres take part and recruit participants to the CARE pilot trial?
2. Can the investigators implement trial procedures correctly?
3. What proportion of screened patients is eligible?
4. What proportions of eligible patients are approached and randomised (and why are eligible patients not approached or not randomised)?
5. What is the distribution of participants between neurosurgery and stereotactic radiosurgery?
6. Do participants adhere to the allocated intervention and follow-up?
7. How complete are baseline, imaging and outcome data?
8. What are the outcome event rates?

9. How do the baseline characteristics, outcome event rates and differences between treatment groups compare to observational data about outcomes during medical management or after medical and surgical management?
10. What estimates of effect size/variability should be used in the design of the CARE definitive main phase trial?
11. What is the sample size required for a definitive trial to address the overall question over a 10-year follow-up?
12. Can the CARE pilot trial data describe care pathways, linked to health states and outcomes, to develop a robust economic model to evaluate cost effectiveness in a CARE definitive main phase trial?
13. Which international research partners in other countries could contribute to the CARE definitive main phase trial?

2.2.2 Primary clinical outcome

Intracranial haemorrhage or new persistent/progressive focal neurological deficit due to brain cavernoma or surgical management (neurosurgery or stereotactic radiosurgery), whether fatal (leading to death within 30 days of the outcome event) or non-fatal.

2.2.2.1 Intracranial haemorrhage

The definition of an intracranial haemorrhage attributable to brain cavernoma is, "a clinical event involving both acute or subacute onset symptoms (any of headache, epileptic seizure, impaired consciousness, new/worsened focal neurological deficit referable to the anatomic location of the cavernous malformation as well as radiological, pathological, surgical, or rarely only cerebrospinal fluid evidence of recent extra- or intra-lesional haemorrhage. The mere existence of a haemosiderin halo, or solely an increase in cavernoma diameter without other evidence of recent haemorrhage, are not considered to constitute haemorrhage" (3).

2.2.2.2 New persistent/progressive focal neurological deficit

The definition of a non-haemorrhagic focal neurological deficit attributable to brain cavernoma is, "a new or worsened focal neurological deficit referable to the anatomic location of the brain cavernoma, which may present with other clinical features of intracranial haemorrhage, but without evidence of recent blood on timely brain imaging or pathological examination, or examination of the cerebrospinal fluid. These cases may be accompanied by an increase in cavernoma diameter alone or oedema on brain MRI (3).

The definition of a focal neurological deficit (not otherwise specified) attributable to brain cavernoma is identical to non-haemorrhagic focal neurological deficit, with the exception that pathological investigation, cerebrospinal fluid examination, or timely brain imaging have not been performed at all or at the correct time to establish whether haemorrhage, oedema, or cavernoma growth underlie the clinical deterioration (3). These focal neurological deficits may be persistent (lasting >24 hours, and staying static or improving), or progressive (lasting >24 hours with further deterioration) (3).

New persistent/progressive focal neurological deficits attributable to brain cavernoma treatment may be referrable to the anatomic location of the brain cavernoma (e.g. haemorrhage after neurosurgical treatment, or radionecrosis from stereotactic radiosurgery) or referrable to other regions of the brain (e.g. intracranial abscess following neurosurgical excision).

2.2.3 Secondary clinical outcomes

During the CARE pilot trial, investigators will collect data on the risk of several clinical primary and secondary outcomes to inform the design of a main phase RCT. The following secondary clinical outcomes will be measured at each 6-month follow-up review:

1. Death not due to a primary clinical outcome
2. Liverpool Seizure Severity Scale plus epileptic seizure frequency (number of seizures in the preceding four weeks, and attainment of one-year seizure freedom)
3. Modified Rankin Scale (mRS) score
4. National Institute of Health Stroke Scale Score (adult or paediatric)
5. EQ-5D-5L in adults and EQ-5D-Y in children
6. Karnofsky Performance Status (KPS) scale in adults and Lanksy Play-Performance Scale (LPPS) in children

We will also collect data to estimate health service use and healthcare and socioeconomic costs during the entire duration of follow-up.

2.2.4 Feasibility metrics proposed to the funder

The NIHR HTA has been provided with the following criteria for success, although these are not specific secondary outcomes of the CARE pilot trial:

- At least 30 sites in the UK and Ireland collaborate
- Project delivered according to the major milestones identified in the NIHR HTA project management plan
- Recruitment to within 10% of target
- Brain cavernoma radiographic diagnosis confirmed by expert neuroradiologist review in >95% of participants recruited
- Retention of >95% of participants at six months
- <10% treatment group switches or loss to follow-up
- QuinteT recruitment intervention is associated with an improvement in recruitment
- CARE definitive main phase trial appears feasible and affordable

3 STUDY DESIGN

The CARE pilot trial is a two-arm, parallel group randomised feasibility trial which aims to estimate the feasibility of performing a definitive main phase RCT comparing medical management to medical and surgical management (with neurosurgery or Gamma Knife stereotactic radiosurgery, according to their availability in clinical practice) for improving outcomes for people with symptomatic brain cavernoma. An integrated QRI aims us to understand recruitment barriers (e.g. related to selection of patients during screening and recruitment processes or equipoise), and optimise informed consent and recruitment processes in the CARE pilot trial (32; 33; 34). Participants will be recruited in secondary care settings in the UK and Ireland, from a collaborative network of research sites, with input from the patient advocacy organisation CAUK. Randomisation will allocate participants to groups in a 1:1 ratio, stratified by preferred type of surgical management, but if there is no clear preference for the type of surgical management, and both are available, the patient will be allocated to either neurosurgery or stereotactic radiosurgery (see section 3.1).

3.1 TRIAL PROFILE

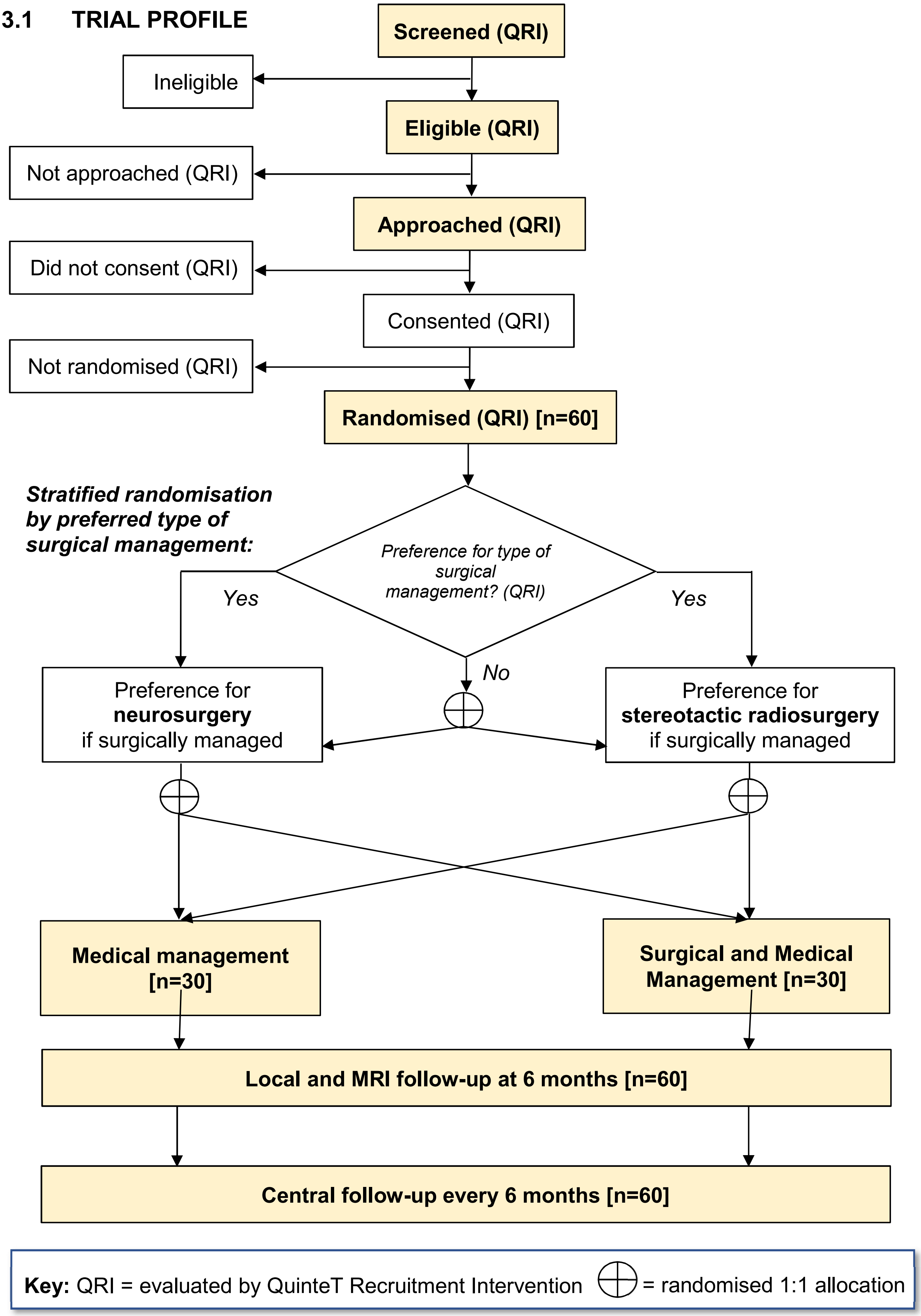

3.1.1 QuinteT recruitment intervention

The QuinteT recruitment intervention (QRI) has been presented as two distinct stages for clarity (data collection followed by feedback and training). In reality these are likely to overlap or run in tandem. For instance, new avenues of enquiry may emerge through feedback meetings, which can be a route to investigating recruitment difficulties in their own right. Insights into recruitment can emerge at any point during the RCT and instigate further investigations or intervention.

3.1.1.1 <u>Phase 1</u>

3.1.1.1.1 Before the CARE pilot trial begins recruitment

The QuinteT researcher will conduct a qualitative evaluation of what may influence recruitment during study set-up, combining evidence from previous QuinteT recruitment interventions (35; 36; 37; 38; 39; 40) and training programmes (41; 42), with data collected from patient and professional groups involved in CARE.

Qualitative work will include focus groups with healthcare professionals to explore views on eligibility and equipoise. Healthcare professionals' views will be explored in online workshops, to which we will invite relevant clinical members of the Trial Management Group (TMG), 'Consultant Cavernoma Contacts' and investigators at collaborating sites. These workshops will explore differences in views between individuals and clinical specialties regarding equipoise and identify criteria to determine patient suitability for neurosurgery or stereotactic radiosurgery, previously identified by the study team as difficult to operationalise. Discussions will also cover patient pathways into the trial, processes and management options for those declining participation, what each intervention arm involves, including potential risks and benefits, plans for follow up within the CARE pilot trial and possible advantages and disadvantages of taking part. We will organise these workshops with clinicians to maximise attendance, convenience, and efficiency by holding them virtually. The work described in this paragraph is for information only and is covered by a separate Research Ethics Committee (REC) approval (University of Bristol, Faculty of Health Sciences Research Ethics Committee Reference 111186). Qualitative work involving focus groups with healthcare professionals is therefore not covered under this protocol.

Insights into patient views to inform development of patient-facing materials, inform the design of the pathway into the trial and provide insight into the acceptability of participation in the CARE pilot trial will be obtained through the QuinteT researcher observing all CARE pilot trial Patient, carer and public involvement Advisory Group (PAG) meetings at which such issues are discussed.

A QuinteT researcher will observe all TMG and TSC meetings during which the study protocol is developed and finalised, with a focus on discussions and final presentation of equipoise and eligibility criteria.

Insights from focus groups with professionals and observation of the TMG, TSC and PAG discussions will inform the content of patient-facing information for the CARE pilot trial and site initiation visits for recruiters. The QuinteT team will provide guidance for recruiters to present CARE pilot trial information to eligible patients, carers and families during site training and initiation (see section 16.2.5.1). Guidance will raise recruiter awareness of key 'hidden' challenges when trying to recruit patients to trials comparing medical management with medical and surgical management and how these can be addressed (35; 42), as well as including insights into particular issues identified as relevant to the CARE pilot trial in how to deal with

preferences and convey equipoise between medical management and medical and surgical management.

3.1.1.1.2 During CARE pilot trial recruitment

As recruitment to the CARE pilot trial begins, recruitment processes will be investigated in-depth at study sites as they open. A QuinteT researcher will use a multi-faceted, flexible approach using triangulation of the following data to investigate site-specific or more general recruitment obstacles (34): screening logs (section 5.3); recording of recruitment consultations between recruiters and patients (section **Error! Reference source not found.**); in-depth interviews with members of the TMG, recruiters, and participants (section 9.4.3); review of study documents (section 9.4.5) and observation of monthly TMG meetings (section 9.4.4).

3.1.1.2 Phase 2

Findings from phase 1 will be presented to the CI and TMG. If recruitment difficulties are evident across the trial or at particular sites, the CI/TMG and QuinteT team will formulate a ‘plan of action’ to improve recruitment and information provision. The specific plan implemented will be grounded in the findings from analysis of the data above, with its format dependent on the nature of the recruitment barriers identified (see section 16.2.5.1).

4 STUDY POPULATION

4.1 NUMBER OF PARTICIPANTS

We aim to enrol approximately 60 participants over an estimated 18 months at approximately 45 sites in the UK and Ireland. Patient follow-up will end approximately 6 months after recruitment finishes.

4.2 INCLUSION CRITERIA

1. People of any age
2. At least one brain cavernoma diagnosed by brain MRI that included a gradient echo or susceptibility-weighted sequence, according to standard diagnostic criteria (15; 43)
3. Clinical history attributable to a brain cavernoma of:
    a. Symptomatic stroke due to intracranial haemorrhage (3), or
    b. Symptomatic stroke due to a persistent or progressive non-haemorrhagic, or not otherwise specified, focal neurological deficit (3), or
    c. Epileptic seizure(s) meeting the definition of definite or probable cavernoma-related epilepsy (44)
4. Patient and doctor are uncertain about medical management or medical and surgical management of the symptomatic brain cavernoma, following consultation with a neurosurgeon
5. Patient has mental capacity to consent for themselves (adult participants or paediatric participants with capacity) or parent/legal guardian provides consent (paediatric participants).

There is no upper time limit on when a patient may be recruited following the symptomatic presentation and diagnosis of a brain cavernoma.

Patients with multiple brain cavernomas, at least one of which has been symptomatic and not undergone removal/obliteration by surgical management, may be included.

In the case of prior surgical management (with neurosurgery or stereotactic radiosurgery), patients with a symptomatic brain cavernoma that has not been completely removed/obliterated by prior surgical management may be included.

4.3 EXCLUSION CRITERIA

1. Surgical management of a solitary symptomatic brain cavernoma with MRI evidence of cavernoma removal/obliteration
2. Spinal cavernoma alone, without symptomatic brain cavernoma
3. Asymptomatic brain cavernoma. Patients with radiographic cavernoma enlargement (with or without intralesional haemorrhage) but without new symptoms are still regarded as asymptomatic.
4. Previously randomised in the CARE pilot trial

4.4 CO-ENROLMENT

Inclusion in another RCT or observational study does not preclude participation in the CARE pilot trial as long as: participants are not overburdened; their inclusion would be unlikely to confound the CARE pilot trial's results or complicate attribution of serious adverse events and outcomes; the protocol of the other study does not preclude co-enrolment in the CARE pilot trial; and co-enrolment has been agreed with the Chief Investigators of all studies involved in co-enrolment. Research staff should obtain permission to enrol patients who are participants in other trials from the CI. A record of participants who are known to have been co-enrolled in other studies will be maintained by the TCC.

5 PARTICIPANT SELECTION AND ENROLMENT

5.1 IDENTIFYING AND SCREENING PARTICIPANTS

For a patient to be eligible for the trial, the patient and doctor must be uncertain about medical management or medical and surgical management of the symptomatic brain cavernoma. In standard clinical practice, decisions about medical management or medical and surgical management of symptomatic brain cavernomas are usually made with patients and neurologists or neurosurgeons, following discussions at multi-disciplinary meetings that may involve any or all of neurologists, neurosurgeons, stroke physicians, and radiologists. We expect uncertainty about medical management or medical and surgical management to be established during discussion between a patient and their doctor. In clinical practice, multidisciplinary meetings involving neurologists and neurosurgeons may confirm this uncertainty as well as suitability for either type of surgical management; sometimes, these multidisciplinary meetings manage this uncertainty by arriving at a consensus opinion, but investigators should note that this may make recruitment to the CARE pilot trial less likely.

The principal investigator (PI), or another clinician with delegated responsibility, is responsible for confirming eligibility for the trial, however delegated research team members can identify eligible patients. Research team members delegated this role should be members of, or affiliated to, the clinical care team. These people may identify potentially eligible patients using several sources at their site, including but not limited to data on admissions, outpatient appointments, referrals, and brain imaging that record:

- New diagnoses of symptomatic brain cavernoma made in everyday clinical practice during the recruitment period.
- Diagnoses of symptomatic brain cavernoma made at any time before the recruitment period, identified by searches of clinical or imaging databases, or clinicians' own records.
- Referrals from colleagues at other hospitals in the UK and Ireland.

Verification of eligibility will require delegated research staff to access patient medical notes.

The TMG will apply to use the Association of British Neurologists' Rare Diseases Ascertainment and Recruitment platform (RaDAR; https://www.theabn.org/general/custom.asp?page=radar), which is used by neurologists to indicate that they have seen a patient with a specified rare neurological disease (such as brain cavernoma). Once a neurologist notifies RaDAR that they have seen a patient, the neurologist will be sent the patient information leaflet about the trial to send to the patient, who can be referred to their local trial site if they are interested in discussing participation.

CAUK (and affiliated groups such as Cavernoma Ireland and Cavernoma Scotland) will share information about the trial through their website, social media platforms and any other communications channels used by them. Patients who contact, or are members of, one of the patient support organisations will be made aware of the CARE pilot trial and informed about what the CARE pilot trial involves by a CAUK member of staff. If these patients are interested in finding out more and being screened for their eligibility, CAUK may direct them to information about a Consultant Cavernoma Contact at an appropriate CARE pilot trial site. The role of CAUK will be provision of information to patients; patients will be advised to speak with their clinician about decisions related to their medical care. CAUK will record the number of patients who they identify as potentially suitable for the CARE pilot trial and suggest referral to a Consultant Cavernoma Contact.

The CI and other members of the TMG will raise awareness of the trial amongst the clinical community through presentations at conferences and meetings. This could result in referral of patients to CARE pilot trial recruitment sites from other hospitals in the UK and Ireland.

5.2 APPROACHING AND CONSENTING PARTICIPANTS

Patients in the UK and Ireland will be approached and invited to take part in adult and paediatric neurology, neurosurgery, and stroke services in secondary care, or one of the stereotactic radiosurgery services that are commissioned to provide stereotactic radiosurgery for cavernoma (29). Eligibility may have been determined by a multidisciplinary discussion, but eligible patients should be approached for recruitment to the CARE pilot trial during or after consultation with a specialist in the type of treatment that is thought to be most effective for the surgical management of the brain cavernoma. Delegated research staff involved in approaching eligible patients should be members of, or affiliated to, the clinical care team.

Potential adult participants or the parent/guardians of potential paediatric participants may approached in person or by telephone (or another technology that supports remote consultations e.g. NHS Near Me). An invite letter may be sent in advance of approaching the patient. The short and supplementary PIL will be used to introduce and discuss the trial.

There is no specific time window for approaching eligible patients for their consent (see section 4.2 above), but they should be approached whenever uncertainty arises about whether to pursue medical management or medical and surgical management of a symptomatic brain cavernoma. The oral explanation given should be performed by the PI or another member of the research team delegated to perform this task and must cover all the elements specified in the relevant PIL and ICF. The patient or the parent/guardian will be given as much time as they require to consider the study information and given every opportunity to ask questions.

The PI or another clinician with delegated responsibility, is responsible for confirming eligibility for the trial, ensuring informed consent is obtained and that the informed consent form (ICF) is signed and dated by all parties before randomisation and any protocol-specific procedures are carried out. Local research staff should follow the laws that govern consent procedures in their jurisdiction. Members of the research team will have undergone standardised training on trial-related procedures. Health Research Authority guidance on applying a proportionate approach to seeking consent has been followed (45). Adult patients lacking mental capacity to consent for themselves will not be included in this trial (see section 4.2). If an adult patient loses mental capacity during the course of the research and subsequently regains mental capacity, their consent to continue taking part in the trial will be confirmed.

Face to face informed consent discussions with potential participants may not be feasible (e.g. due to the COVID-19 pandemic). In order to avoid patients making additional trips to hospital, written informed consent may be recorded in the following ways (in addition to being done in person):

1. Remotely

When completed remotely, the patient should return the signed form, or a scan or legible photograph of all sections of it, to a research team member at the recruiting site by email, by post or in person..

2. Electronically (using an online form)

The following options may be employed to complete consent electronically:

- The consent form may be completed and signed electronically where an approved mechanism is available such as DocuSign.
- An electronic consent form, generated via the trial database. Participants providing consent using the online form will be required to enter a typewritten signature.

In both cases, the form should be countersigned by the research team member taking consent. There is no requirement that the counter-signature date match the date of the participant signature but the counter-signatory must be satisfied that the consent is genuine.

Regardless of the method of consent, patients or parent/guardians will be provided with information in-person, by post or by email to consider before providing consent.

The information will be discussed with the patient or parent/guardian as outlined above.

Confirmation of eligibility, consent, and the version of the PILs used should be recorded in the participant's paper and/or electronic medical records for any future source data verification, including the date of consent (and child's assent if relevant), that the participant received the PILs, who obtained consent, and signed and dated confirmation that the patient was eligible for enrolment.
Patients will be given the opportunity to consent to any or all of the following:

- Consent to recording their recruitment consultation(s) to inform the QuinteT recruitment intervention
- Consent to taking part in an interview to inform the QuinteT recruitment intervention
- Consent to participate in the CARE pilot trial

5.2.1 Consent to the QRI

All eligible patients who are approached to take part will be invited to take part in an interview with the qualitative researcher about their experiences of being invited to join the CARE pilot trial.

Some study centres will also be involved in audio-recording conversations where the CARE pilot trial is discussed (including conversations held in person and by remote methods). In study centres selected to participate in collecting audio-recordings, eligible patients will be invited to consent to these conversations being audio-recorded, before discussion of the CARE pilot trial begins. Information on the rationale and process for recording recruitment discussions is covered in the relevant CARE PIL. Missed recordings of recruitment conversations are not required to be recorded as protocol deviations.

Participants will be given sufficient time to consider whether they wish to take part in the QRI. Participants will only be consented if they and the local research team feel they have had enough time to consider and ask questions about the QRI. Consent to take part will be documented on the relevant verbal and/or written consent forms. Written consent to audio-recordings will cover all future recruitment discussions. Patient participation in both interviews and audio-recordings is optional. If written consent to record conversations is given, the recordings will be transferred to the University of Bristol for analysis (see section 10.3.1). If no written consent form is received, all recordings for that participant will be deleted, no further recordings will be made and no invitation to interview extended.

5.2.2 Consent to participate in the CARE pilot trial

5.2.2.1 Adults

The participant will be asked to complete a consent form. The research team member and the participant should each sign and date the ICF to confirm that consent has been obtained. Written informed consent should always be sought from the participant where possible. If this is not possible because the participant cannot write, the member of the research team can gain witnessed verbal consent. The participant should receive a copy of the completed ICF, a copy should be filed in the patient's medical records and the original ICF should be filed in the investigator site file (ISF) along with the randomisation form. The participant should also receive a copy of the current PIL.

5.2.2.2 Children

Childrens' PILs are available for children 0-5 years old, 6-10 years old and 11-16 years old. Children aged 6-10 and 11-15 who are capable of understanding it will be given the option of providing assent.

The parent/guardian should receive a copy of the current parent/guardian short and supplementary PIL and appropriate children's PIL. If the parent/guardian wishes for the child to participate in the CARE pilot trial, then they will be asked to sign the ICF. Both the parent/guardian and the person delegated to take consent will each sign and date the ICF. The parent/guardian should receive a copy of the fully completed ICF, a copy should be filed in the patient's medical records and the original ICF should be filed in the investigator site file (ISF) along with the randomisation form. The same would apply in the case of assent being given.

5.2.2.2.1 Children and young people in England, Wales and Northern Ireland

Health Research Authority (HRA) guidance states (46):

- "There is no statute in England, Wales or Northern Ireland governing a child's right to consent to take part in research other than a Clinical Trial of an Investigational Medicinal Product (CTIMP), i.e. consent for non-CTIMPs. However common law presumes that young people aged between 16 and 18 are usually competent to give consent to treatment."
- "Case law suggests that if a young person has sufficient understanding and intelligence to understand fully what is proposed, and can use and weigh this information in reaching a decision (i.e. they are 'Gillick competent'), he or she can give consent to treatment."
- "In the absence of law relating specifically to research, it is commonly assumed that the principle of 'Gillick competence' can be applied not only to consent for treatment, but also to consent for research."
- "When a young person is believed to be competent, consent from those with parental responsibility is not legally necessary. However, the involvement of parents in decision-making is encouraged in most circumstances."
- "When a child or young person is not competent, the Children Act and the Children Act (Northern Ireland) Order permits parents (and those with parental responsibility) to consent to medical treatment on their behalf. Consent of only one parent is required."

5.2.2.2.2 Children and young people in Scotland

Health Research Authority (HRA) guidance states (47):

- "There is no specific provision in Scots law governing a child's right to consent to take part in research, other than a Clinical Trial of an Investigational Medicinal Product (CTIMP), i.e. consent for non-CTIMPs."
- In the case of medical treatment, "young people aged 16 and over are deemed to be competent to give consent for medical treatment unless proven otherwise. Children and young people under 16 have a statutory right to give consent to surgical, medical or dental procedures or treatments if they are deemed, by a medical practitioner, to be competent to do so."
- "It is commonly accepted that we can extrapolate a child / young person's right to give consent for treatment, to give them the right to give consent to

take part in non-CTIMP research. It is commonly assumed that they also have a legal right to object to participation."

- "The Children (Scotland) Act permits parents (or those with parental responsibility) to give consent on behalf of a young person under 16 who is not competent. Consent of only one parent is required."

The above guidance will be followed for this trial in relation to participants in Scotland under the age of 16.

5.2.2.2.3 Children and young people in the Republic of Ireland

Consent will be obtained in line with ICH-GCP and all applicable laws and regulations. In line with the HSE National Consent Policy, consent to a child's participation in a study must be obtained from a parent/legal guardian for all paediatric participants under 18 years old (48). Whenever the child has sufficient competence to provide it, a child's assent must be sought in a child-appropriate manner.

5.2.2.2.4 Re-consenting paediatric patients

When a child recruited into the trial reaches the age of 16 years (or 18 years old in the Republic of Ireland) and is therefore deemed competent to provide consent, they should be re-consented if still willing to participate at their next 6-month follow up review. No further data will be collected until a signed consent form has been received.

5.2.3 Consent to be contacted for an interview exploring reasons for declining participation

Patients or their parents/carers who decline participation in the CARE pilot trial will be invited to consent to take part in an interview with the QRI researcher, exploring their experiences of being approached and invited to take part in the study. Where parents/carers consent to take part in an interview, it will be acceptable for the child/young person to attend and contribute if they choose.

5.3 SCREENING AND ENROLMENT LOGS

Research teams at each site will use screening logs to record non-identifying demographic and clinical details of patients who are screened, including: initials, age (years), sex, brain cavernoma diagnosis (yes vs. no), brain cavernoma location (brainstem vs. other), type of brain cavernoma presentation (symptomatic [type] vs. not symptomatic), prior treatment of brain cavernoma, patient certainty about brain cavernoma treatment (yes vs. no, with preferences), clinician certainty about cavernoma treatment (yes vs. no, with preferences), eligibility for the CARE pilot trial (yes vs. no, with reasons for ineligibility), whether approached to take part (yes vs. no, with reasons for not approaching), whether consent was given to the CARE pilot trial (yes vs. no, with reasons for declining), and whether the patient was randomised in the CARE pilot trial (yes vs. no, with reasons for not being randomised and preferred management outside of CARE).

Collection of this information is essential to fulfilling the objectives of the feasibility study that will determine whether a CARE definitive main phase trial could proceed

(see section 2.2.1 above). The proportions of screened patients who are eligible, approached, agree to take part, and randomised (see trial profile, section 3.1) will be quantified to identify points in the recruitment pathway at which patients are being 'lost' to recruitment. Screening logs will be analysed according to the SEAR (Screened, Eligible, Approached, Randomised) framework (49).

5.4 RANDOMISATION

5.4.1 Randomisation procedures

If consent to randomisation in the CARE pilot trial is provided, complete baseline data must be collected by the research team at the baseline visit before randomisation. These data include demographic, clinical, and radiographic information, as well as the consensus preference agreed between each patient and their clinician for neurosurgery or Gamma Knife stereotactic radiosurgery should randomisation allocate them to medical and surgical management (if there is no clear preference for the type of surgical treatment, and both are available in clinical practice, the patient will be randomly allocated to neurosurgery or Gamma Knife stereotactic radiosurgery; see section 3.1). Participants in these two strata will be assigned 1:1 to medical management or medical and surgical management using permuted blocks. Allocation will be concealed until participants are enrolled and assigned by using central web-based randomisation.

A detailed description of the randomisation system including details on block size is held in the statistics master file by Edinburgh Clinical Trials Unit (ECTU).

5.4.2 Treatment allocation

The participant, or the parent/guardian of paediatric participants, and research team at the recruiting site will be notified of the assigned treatment allocation after randomisation.

5.4.3 Blinding (masking)

Treatment allocation in the CARE pilot trial is not blinded (masked), and is therefore open to participants, the clinicians caring for them and local research staff.

We will aim to keep outcome event assessors blind to treatment allocation. We will aim to measure how often assessors are unblinded to treatment allocation during the process of event adjudication.

5.5 WITHDRAWAL OF PARTICIPANTS

Participants are free to completely withdraw, or discontinue any individual component of the study, at any point or a participant can be withdrawn by the PI. In the case of loss of mental capacity in adult participants during the trial, researchers will follow the appropriate local regulations and guidance regarding loss of mental capacity in research (noting that these differ between nations, see below). The participant will remain in the trial unless withdrawn by their representative. Data collected until the time of withdrawal will be retained. If withdrawal occurs, the primary reason for

withdrawal must be documented in the participant’s case report form (CRF). The participant will have the option of withdrawal from any or all of:

- consent to be contacted about other research studies
- consent to recording of recruitment conversation(s)
- consent to complete a recorded interview with the QuinteT researcher
- DNA sample provision
- allocated treatment policy
- in-person follow-up
- brain MRI at 6-months
- participant postal follow-up questionnaires
- participant follow-up questionnaire conducted by telephone
- long-term follow-up using record linkage
- use of de-identified data or brain imaging by other research studies

5.5.1 Loss of mental capacity in adult participants in England and Wales

In England and Wales, regulations advise that advice should be sought from the participant's representative on whether the research should be carried out in relation to the participant and what they think the wishes and feelings of the participant would be if they had mental capacity (50).

Where the participant representative (consultee) requests that the participant who has lost mental capacity be withdrawn, a delegated member of the research team will discuss with this person to determine if they think the participant should be withdrawn taking into consideration what the wishes and feelings of the participant would be thought to be if they still had the mental capacity to decide for themselves. If it is agreed that the participant should be withdrawn from the trial, the appropriate trial form will be completed.

5.5.2 Loss of mental capacity in adult participants in Scotland

In Scotland, there is no specific legal provision for adults who lose capacity while taking part in non-CTIMPs. We will respect the participant's original consent to take part however will also consider the participant's representative's views.

Where the participant representative (nearest relative, welfare attorney or welfare guardian) requests that the participant who has lost mental capacity be withdrawn, a delegated member of the research team will discuss with this person to determine if they think the participant should be withdrawn taking into consideration what the wishes and feelings of the participant would be thought to be if they still had the mental capacity to decide for themselves. If it is agreed that the participant should be withdrawn from the trial, the appropriate trial form will be completed (51).

5.5.3 Loss of mental capacity in adult participants in Northern Ireland

In Northern Ireland, section 138 of Part 8 of the Mental Capacity Act (Northern Ireland) 2016 applies which states that consent can be considered to endure provided that the study has not changed significantly since consent was given. We will respect the participant's original consent to take part however will also consider the participant's representative's views.

Where the participant representative (consultee) requests that the patient who has lost mental capacity be withdrawn, a delegated member of the research team will

discuss with this person to determine if they think the participant should be withdrawn taking into consideration what the wishes and feelings of the participant would be thought to be if they still had the mental capacity to decide for themselves. If it is agreed that the participant should be withdrawn from the trial, the appropriate trial form will be completed (52).

5.5.4 Loss of mental capacity in adult participants in the Republic of Ireland

Health Service Executive Policy (48) states that:

"Outside of clinical trials, there is currently no legal framework for a person who lacks decision-making capacity to participate in research. In the absence of any such legal regulations, it is recommended that as a matter of best practice the same principles should apply to both clinical trials and other forms of research. This means that consent for participation in any form of research on behalf of an adult lacking decision-making capacity must be obtained from the person's legal representative".

The same policy defines 'legal representative' as:

"…a person not connected with the conduct of the trial who by virtue of his/her family relationship with an adult lacking decision-making capacity, is suitable to act as the legal representative and is willing and able to do so or (if there is no such individual) a person who is not connected with the conduct of the trial, who is a solicitor nominated by the relevant health care provider.".

6 COMPARATOR

Medical management constitutes standard medical care alone for brain cavernoma, according to UK guidelines (8). This may include anti-epileptic drug therapy to prevent epileptic seizures (e.g. following the recommendations of the Surgical Task Force of the ILAE Commission on Therapeutic Strategies (44)), rehabilitation of neurological deficits (e.g. physiotherapy, speech and language therapy), medical treatment of other neurological symptoms (e.g. headache, body pain, spasticity, dysaesthesia), and psychological support. Provision of these interventions varies because of the extent of the evidence to support their use, and their availability in everyday clinical practice around the UK and Ireland according to the nature of regional and national healthcare systems.

Some clinicians arrange repeat brain MRI for patients with brain cavernoma. This may be done with good reason in order to confirm the diagnosis following intracranial haemorrhage, in case of diagnostic doubt, to guide treatment decisions, or to investigate new symptoms as recommended by recent guidelines (15). But in other cases repeat brain MRI is done to 'monitor' brain cavernomas to reassure patients, although the evidence that this strategy is beneficial is lacking.

7 INTERVENTION

Medical and surgical management in the CARE pilot trial is defined as neurosurgical excision or Gamma Knife stereotactic radiosurgery for brain cavernoma, in addition to all components of medical management described in section 6 above. These interventions will be accessed and delivered according tco what is available in standard clinical practice in the participant's health service.

It is expected (but not mandated by the trial protocol) that surgical management will be delivered within 3 months of randomisation to the trial.

7.1 Neurosurgical excision

Surgery will be undertaken by a consultant neurosurgeon responsible for neurosurgical aspects of the clinical care of the cavernoma patient in CARE. The neurosurgical technique employed will be that used by the consultant neurosurgeon in clinical practice. Adjuncts such as image direction, microscopy, ultrasonic aspiration, awake/general anaesthesia surgery, cortical mapping/stimulation, and intra-operative MRI, will be used as considered appropriate by the consultant neurosurgeon.

It is recommended (but not mandated by this protocol) that a post-operative MRI scan is performed within 72 hours of surgery and used along with the surgeon's assessment to confirm complete resection or incomplete resection. A copy of this scan will be taken by the research team and uploaded to the scan database for the trial.

7.2 Stereotactic radiosurgery

Stereotactic radiosurgery will be performed at the National Centre for Stereotactic Radiosurgery in Sheffield or the Queen Square Radiosurgery Centre, which are the two referral centres in the UK that are commissioned to provide Gamma Knife stereotactic radiosurgery for cavernoma (29).

Standard clinical treatment protocols will be used which involve targeting the brain cavernoma, but not the surrounding haemosiderin ring. Treatment dosages will range from 12-16Gy depending on size, shape, definition and site of the cavernoma.

If ICH has occurred from the cavernoma, Gamma Knife stereotactic radiosurgery will be carried out once the haematoma is judged to have been reabsorbed to minimise radiation exposure and reduce volume of treatment as much as possible.

8 STUDY ASSESSMENTS

8.1 STUDY ASSESSMENTS

This section outlines the study assessments to be completed by the research team. The schedule of study assessments is provided on the following page.

8.1.1 Table of assessments

Assessment	Identification and Screening	Baseline visit	Within 3 months of baseline	6-month local in-person follow-up	6-monthly central follow-up
Assessment of eligibility	X				
Screening end enrolment logs	X				
Consent to recruitment conversation recordings	X [1]				
Consent to qualitative interview	X				
Recording of patient recruitment conversations	X [2]	X [2]			
Consent to randomisation	X [3]	X [3]			
Demographic, clinical, socio-economic, medication, and radiographic data		X			
DNA sample		X			
Provision of diagnostic brain imaging		X			
Randomisation		X			
Questionnaires		X		X	X
Cavernoma surgical management			X		X
Repeat brain MRI				X	
Outcomes and adverse events				X	X
Qualitative interview			X [4]		

1 – Research teams will be asked to capture verbal consent to audio-recordings of recruitment conversations when the approach is made to the participant. If this is not possible at this time, consent may be captured during subsequent recruitment conversations.

2 – Recordings of recruitment conversations with patients should be captured (as requested) wherever the CARE pilot trial is discussed (illustrated here but not restricted to Screening and Baseline Visit).

3 – Consent to participation in CARE may be collected at the Baseline Visit or in advance, during the Screening stage.

4 – Interviews with patients will take place within 3 months of being invited to take part in the trial.

8.1.2 Screening

Potential participant identification and screening should be carried out as per sections 5.1 and 5.2.

Approached patients who decline to take part will be given the opportunity to take part in an interview to discuss why they decided not to participate as per section 5.2.3.

Research teams should complete screening and enrolment logs as per section 5.3.

8.1.3 Informed consent

It is likely that consent to participate in the CARE pilot trial will be captured during a clinical consultation between the patient and a clinician who is also a member of the CARE pilot trial research team. The consenting procedures outlined in section 5.2. will be followed.

8.1.4 Baseline visit

Baseline visits may be conducted remotely or in person, depending on patient, carer or parent/guardian preference, and restrictions on working practices. These visits will be conducted by research team staff who are members of, or affiliated to, the clinical care team.

Research team staff will collect the following data at the baseline visit from all study participants: demographics, socioeconomic characteristics (e.g. employment, education, and carer needs), medical history (including details of the type of presentation of the symptomatic brain cavernoma and family history) and medications (including drug therapy).

The patient reported questionnaires that should be completed are EQ5D-5L for adults or EQ5D-3Y for children and Liverpool Seizure Severity Scale (LSSS).

The patient should be assessed by the research team member (assisted by parent/guardian where required) using the following scales:

1. Modified Rankin Scale (mRS) score
2. National Institute of Health Stroke Scale Score (adult or paediatric) (if examined in person)
3. Karnofsky Performance Status (KPS) scale in adults and Lanksy Play-Performance Scale in children (LPS)

If the visit is done face to face, research team staff will collect a venous blood sample of up to 10mL from patients who consent into an EDTA tube for genetic analysis. Samples will be shipped immediately by first class post and in adherence with UN3373 guidelines to the central laboratory at the Edinburgh Clinical Research Facility.

The research team at each site is responsible for entering these data onto the study Electronic Case Report Form (eCRF). Once baseline data are complete, randomisation may proceed. After randomisation is performed, the PI and other research staff on the delegation log at the participant's site will be sent email

confirmation or randomisation and treatment allocation, with a reminder about the subsequent scheduled activities in the trial.

Research teams will upload the relevant pseudo-anonymised DICOM images of the brain imaging (including diagnostic brain MRI) that confirmed the mode of presentation and diagnosis of the symptomatic cavernoma to the trial imaging database. Images may also be copied to CD and posted to the brain imaging management team for upload. These scans will be stored for subsequent validation by a senior neuroradiologist to confirm or refute eligibility.

8.1.5 Three-month adherence check

The PI and research staff at a site where a participant was randomised will be sent an email prompt around three months after baseline to report whether surgical management was undertaken after randomisation, regardless of whether the participant was allocated to surgical management by randomisation. This will allow detection of cross-overs between the two arms of the trial.

Adherence to the randomised allocation will be assessed by comparing treatment allocation with the completion of the surgical management case report form. Lack of adherence to the randomised treatment allocation will not be recorded as a protocol deviation or violation.

8.1.6 Six-month local follow-up visit

Participants will be asked to attend for their first six-month follow-up visit in person in order to perform brain MRI (which will be permitted between 5-7 months after randomisation) to assess cavernoma presence and size as a measure of the efficacy of surgical management. These images should be uploaded to the trial imaging database or research teams may post CDs to the MRI management team for upload. The radiology department at each site will issue the clinical report of any brain MRI performed for the CARE pilot trial. A copy of MRI brain scans performed before or after surgical management (if performed) will be taken by the research team and uploaded to the scan database for the trial. A copy of the MRI performed on the day of treatment for patients undergoing stereotactic radiosurgery will be taken by the research team and uploaded to the database for the trial (or copied to CD and posted to the MRI management team for upload).

Research teams will record details of any clinical outcome events that have occurred since randomisation, whether surgical management was used, including specific operative techniques or methods of stereotactic radiosurgery. Although surgical management in the CARE pilot trial will continue to be neurosurgical excision or stereotactic radiosurgery, we will collect details of each type of surgical management used after randomisation to allow us to quantify the use of emerging technologies, such as minimally invasive therapeutic approaches for brain cavernoma such as magnetic resonance thermography-guided laser interstitial thermal therapy, or stereotactic laser ablation (41).

Imaging studies performed because of the occurrence of an outcome event will be collected by the research team and uploaded to the scan database for the trial.

The patient reported questionnaires that should be completed are EQ5D-5L for adults or EQ5D-3Y for children and Liverpool Seizure Severity Scale (LSSS).

The patient should be assessed by the research team member (assisted by parent/guardian where required) using the following scales:

1. Modified Rankin Scale (mRS) score
2. National Institute of Health Stroke Scale Score (adult or paediatric) (if examined in person)
3. Karnofsky Performance Status (KPS) scale in adults and Lanksy Play-Performance Scale in children (LPS)

If a blood sample for genetic analysis was not collected as the Baseline Visit, research team staff will collect a venous blood sample of up to 10mL from patients who consent into an EDTA tube. The sample will be shipped immediately by first class post and in adherence with UN3373 guidelines to the central laboratory at the Edinburgh Clinical Research Facility.

8.1.7 Six-monthly central follow-up visit

Thereafter, staff at the TCC, will perform six-monthly follow-up (+/- one month) by post in all patients who do not withdraw from follow-up in the CARE pilot trial, after checking the participant's vital status with their general practitioner. If a response is not received by the TCC within a fortnight, a research team member (based within ECTU) will contact non-responders and follow-up data by telephone or email.

Follow-up questionnaires will confirm participants' current domicile and general practitioner, and ask about disability, health-related quality of life, the occurrence of primary or secondary clinical outcomes, serious adverse events, and the occurrence of surgical management of the brain cavernoma (as described above). These questionnaires will also ask for information about relevant concomitant medications, such as anti-epileptic drugs. We will also record the use of drugs like propranolol, antiplatelet agents, anticoagulant agents and statins, which may have disease-modifying effects (49).

The patient reported questionnaires that should be completed are EQ5D-5L for adults or EQ5D-3Y for children and Liverpool Seizure Severity Scale (LSSS).

The patient should be assessed by the research team member (assisted by parent/guardian where required) using the following scales:

1. Modified Rankin Scale (mRS) score
2. Karnofsky Performance Status (KPS) scale in adults and Lanksy Play-Performance Scale in children

8.1.8 Patient Interviews

In-depth interviews will be conducted by the qualitative researcher with a sample of eligible patients who have been approached to take part in the trial (including those accepting or declining participation) (see section 9.4). Purposive sampling will be used to identify patients who have declined participation from a variety of study sites, to gain insight into study-wide and site-specific reasons patients may have for declining. Purposive sampling of patients accepting participation in the CARE pilot trial will also be considered if findings from analysis of recorded recruitment conversations indicates this will be helpful. Interviews will take place within three months of the decision about trial participation (see 8.1.1).

8.2 LONG TERM FOLLOW UP

We will ask study participants to consent to long-term follow up (i.e. beyond the planned follow-up in the CARE pilot trial), including the use of routinely collected data (such as hospital admissions, procedures, and death certificates), in case the CARE pilot trial is successful and runs seamlessly into a definitive main phase trial.

8.3 BRAIN MAGNETIC RESONANCE IMAGING

Participants who consent to be randomised should undergo repeat brain MRI once at six months (± one month) after randomisation.

Brain MRI is usually undertaken after surgical management in clinical practice, but not always during medical management. If a participant undergoes brain MRI with the required sequences as part of their routine clinical care before the 6-month local follow up visit, the research team will request the brain MRI and upload the scan to the trial imaging database. Otherwise, repeat brain MRI should be performed six months after randomisation (± one month), regardless of treatment allocation, treatment received, and timing of treatment, for research purposes.

As a minimum standard, T1-weighted, T2-weighted, and haem-sensitive sequences (gradient recalled echo or susceptibility weighted imaging) will be required within standard sequence parameters and with an acceptable slice thickness and voxel size. We will collect any other sequences performed (e.g. Fluid Attenuated Inversion Recovery (FLAIR) post-contrast, T1 or FLAIR, and Diffusion-Weighted Imaging [DWI] sequences) to ascertain the frequency of their use for follow-up of brain cavernoma in everyday clinical practice.

8.4 OUTCOME EVENT ADJUDICATION

Clinical outcomes including death and stroke-like events will be adjudicated by a member of the TMG using all available source data (with patient identifiers and any information about cavernoma treatment redacted by the research team before upload to trial database) including clinical correspondence, brain imaging reports, and death certificate. Brain imaging performed during follow-up will be reviewed by a consultant neuroradiologist. Outcome assessors will aim to remain blinded to the brain cavernoma treatment policy that was allocated at randomisation, and if possible any medical and surgical management of the brain cavernoma received. If blinding could not be maintained, this will be documented.

8.5 DNA SAMPLE STORAGE AND ANALYSIS

A venous blood sample of up to 10mL will be collected into an EDTA tube for genetic analysis. Samples will be shipped immediately by first class post and in adherence with UN3373 guidelines to the central laboratory at the Edinburgh Clinical Research Facility for DNA extraction and future analysis. This sample will be stored for subsequent investigation of genetic modifiers of treatment effect, which are currently unknown (1). The relevant approvals will be sought for future research involving these samples.

9 DATA COLLECTION

Data items to be collected are described in section 8. This section describes the methods of data collection.

9.1 SOURCE DATA DOCUMENTATION

Source documents are those in which information is recorded and documented for the first time. The location of source data collected from the CARE pilot trial participants is detailed in the CARE pilot trial Source Data Plan. Investigators will be required to retain paper copies of completed ICFs. Otherwise, clinical data will be entered directly into the eCRF by the research team and TCC staff based on information in the medical records, which will be regarded as source data.

9.2 CASE REPORT FORMS

Documents reflecting the data required at each study assessment will be made available to research teams, to support entry into the study database of: Screening Log, Consent to Contact form, Consent and Status Log, Baseline Visit CRF, 6-Month Follow-up CRF, Serious Adverse Events Log and Change of Status form. Site research teams will be responsible for transcribing these data into the database. Data will be transcribed by those staff delegated to do so on the delegation log held at site.

9.3 STUDY DATABASE

The study database will be created and maintained by ECTU. This database will be compliant with the relevant regulations and Sponsor Standard Operating Procedures (SOPs). Trained and delegated members of the research team will be given password-protected logins to the database. The data will be stored in a secure server in the University of Edinburgh.

9.4 QRI DATA COLLECTION

9.4.1 Screening log data

Screening logs will collect de-identified data on patients screened, identified as eligible, approached and accepting randomisation into the CARE pilot trial (see section 5.4) and identify points in the pathway where patients may be ‘lost’ to recruitment. Findings will guide data collection using the qualitative methods outlined below.

9.4.2 Recordings of recruitment conversations

Patients will be invited to consent to the recording of all conversations during which participation in the CARE pilot trial is discussed. These conversations provide insight into both how the study is presented to patients and how patients interpret that

information. Analysis of these conversations can reveal misunderstandings about that trial that can then be addressed in recruiter training.

9.4.3 Patient and staff interviews

A sample of eligible patients who have been approached to take part in the trial (including those accepting and declining participation) will be invited to take part in an in-depth interview with the qualitative researcher based at the University of Bristol. This interview will take place within three months of being invited to take part in the trial.

Interviews with patients will explore views on the presentation of trial information, understanding of study processes (e.g. randomisation), and reasons underlying decisions to consent or decline to participate in the CARE pilot trial. Numbers of interviews will be guided by the concept of ‘data saturation’ with final sample size (up to a maximum of 20 interviews) determined by the point at which three new interviews fail to shed insights.

Staff involved in the trial will also be invited to take part in an in-depth interview. Interviews with health professionals will use purposeful sampling. Interviews with staff will include members of the trial TMG, including the CI, and those closely involved in the design, management leadership and coordination of the trial (approximately n=4-8); clinicians or researchers involved in trial recruitment (approximately n=12-20).

Interviews with TMG members and investigators at sites will investigate their perspectives on the CARE pilot trial and experiences of recruitment (where relevant). Key topics explored will include views about the study design and protocol; understandings of the evidence on which the study is based; perceptions of uncertainty/equipoise in relation to the intervention arms; views about how the arms/protocol are delivered in clinical centres; methods for identifying eligible patients; views on eligibility, and examples of actual recruitment successes and difficulties.

Interviews will take place at a mutually convenient time by telephone or video-conferencing and will be recorded using University of Bristol approved methods for data capture and storage (this may include MS Teams and Zoom, depending on current policies).

9.4.4 Meetings

A QuinteT researcher will observe all TMG and TSC meetings during which the study protocol is developed and finalised, with a focus on discussions and final presentation of equipoise and eligibility criteria.

9.4.5 Trial documentation

The QRI team will continue to review the wording of patient information leaflets (PIL) and consent forms in line with any feedback from the above that indicates content that is unclear or potentially open to misinterpretation.

10 DATA MANAGEMENT AND TRANSFER

10.1 PERSONAL DATA

The following personal data will be collected as part of this research: contact details (including home address, telephone numbers, email address,date of birth and contact information for relatives/carers), demographic information (including age and sex), socioeconomic information, medical history (including prior symptoms from brain cavernoma, major co-morbidities, medication history, family history), and unique healthcare identifier (such as the Community Health Index [CHI] in Scotland, NHS Number, or equivalent in other nations). Unique healthcare identifiers will be collected to enable long term patient follow-up and ensure correct identification of patients when contacting GPs or sites for follow-up.

Personal data will be processed by site research teams, the TCC at the University of Edinburgh and qualitative research staff at the University of Bristol:

- Personal data will be stored at site by research teams on NHS computers (desktop and laptop). Computers will be password protected and kept in locked offices. All paper files containing personal data will be held in filing cabinets in NHS offices that will be locked when unattended. Study documentation will be accessed by the study team only.
- Personal data will also be entered into the secure trial database which will be hosted on a University of Edinburgh server and will be accessed by the TCC to perform 6-monthly follow-up with patients and long term follow up via record linkage.
- Contact information will be accessed by/passed to the qualitative researcher based at University of Bristol to contact patients for interview.
- Screening log data will be accessed by the qualitative researcher based at University of Bristol as part of the research.

Additional information on personal data in relation to the qualitative aspect of the trial is included in section 10.3.

10.2 BRAIN MRI SCANS

Diagnostic brain imaging will be managed by the Systematic Management, Archiving & Reviewing of Trial Images Service (SMARTIS) at the University of Edinburgh. We will establish a scan database (housekeeping system) using established models, to track all scan episodes, completeness and assessments; this will interface with the trial database. De-identified brain MRI scans will be uploaded to this database by research teams or by SMARTIS staff if CDs are posted to them. Scan collection, quality assurance, curation, and backup will be conducted by SMARTIS staff at the Brain Research Imaging Centre (BRIC), University of Edinburgh. Prof Phil White, or another neuroradiologist involved in the trial, will review the diagnostic and follow-up brain MR imaging using standardised review proforma derived from pre-existing validated work (Scottish Audit of Intracranial Vascular Malformations - SAIVMs).

10.3 QUINTET RECRUITMENT INTERVENTION

10.3.1 Recordings of recruitment conversations

Recruitment conversations will be recorded by a research team member using a method of secure data capture and storage in line with University of Bristol procedures (as outlined on the University of Bristol website). Audio-recordings will be transferred by secure data transfer by the approved qualitative research team members onto a secure drive at the University of Bristol for long-term storage and analysis. Audio-recordings will be labelled with the participant identification number; identifiable patient details will not be used.

Audio-recordings will be subject to targeted transcription and edited to protect the anonymity of respondent. Transcription will be undertaken by an approved transcription service/transcriber that has signed the necessary confidentiality agreements with the University of Bristol. Data will be managed using NVivo software and stored on encrypted drives at the University of Bristol, in line with the university's data storage policies and in line with GDPR legislation.

At the end of the study, audio-recordings will be kept for at least 10 years before they will be destroyed. Transcripts will be stored indefinitely in secure research data storage designated 'controlled access', so can only be accessed by approved individuals who are interested in conducting their own analyses of the data. These individuals will have to submit an application to do this, which will be assessed by an independent committee. However, all data will have identifiable information removed before they are made available, and there will be no way to identify any individuals mentioned in interviews/appointments.

10.3.2 Interviews

Approved qualitative research team members from University of Bristol will access participants' contact details via the trial database or be securely passed them by the research team for the purposes of contacting patients who have consented to interviews as part of the QRI. Team members will be provided with an individual user account for the database with restricted, password-controlled access.

Interviews with patients and staff will be recorded directly by the qualitative researcher using processes for secure data capture and storage in line with University of Bristol procedures (as outlined on the University of Bristol website). Recordings will be held on a secure drive with restricted access at the University of Bristol for long-term storage and analysis. Recordings will be labelled with the participant identification number; identifiable patient details will not be used. At the end of the trial, recordings will be held for a minimum of 10 years after which they will be destroyed.

Data from the QRI will be shared at the end of the trial as outlined in section 17.3.

10.3.3 QRI documentation

Paper or electronic documentation which is generated through the process of performing the QRI will be stored securely at the University of Bristol with access restricted only to approved personnel.

10.4 DATA CONTROLLER

The University of Edinburgh and NHS Lothian are joint data controllers.

10.5 DATA BREACHES

Any data breaches will be reported to the University of Edinburgh and NHS Lothian Data Protection Officers who will onward report to the relevant authority according to the appropriate timelines if required.

11 STATISTICS AND DATA ANALYSIS

11.1 SAMPLE SIZE CALCULATION

Symptomatic brain cavernoma incidence data indicate that ~240 people would be newly-diagnosed during 18 months of recruitment (4). We aim for all of these patients to be screened, but if 10% are missed and 10% decline to participate, we expect research teams to identify ~190 patients. In the ARUBA trial, 226/726 (31%) of the eligible patients approached were randomised (30), so we expect ~60 patients with symptomatic brain cavernoma to be randomised in the CARE pilot trial.

11.2 PROPOSED STATISTICAL ANALYSES

In this pilot phase, analyses are descriptive only, and there will be no formal statistical tests.

We will quantify the number and proportions (with 95% confidence intervals to reflect their precision) of patients who are screened, eligible, approached, consent and are randomised. We will construct a CONSORT diagram to summarise the distribution and progress of participants in the trial including the numbers of withdrawals (50).

We will report descriptively the following: the number and the proportion of the collaborating sites that take part and recruit participants to the CARE pilot trial; research teams' implementation of trial procedures measured by number and type of protocol deviation; the numbers of participants allocated to neurosurgery and stereotactic radiosurgery; adherence to the allocated intervention; completeness of follow-up that would be due at each 6-month interval; completeness of baseline, imaging and outcome data; the frequency of outcome events overall and in an intention-to-treat analysis keeping patients in the treatment group to which they were allocated during all available follow-up.

We will also compare descriptively the characteristics of eligible patients who are screened and do not participate in the CARE pilot trial to eligible patients who are randomised using the characteristics recorded on the screening logs to assess generalisability (external validity) and any recruitment bias.

We will assess measures of functional outcome, to assess which has suitable statistical properties for use in a main phase trial (such as lack of floor/ceiling effects). We will assess whether such a measure (like the method we have used before (9)) would be more suitable as a primary outcome in place of intracranial haemorrhage.

11.3 QUINTET RECRUITMENT INTERVENTION DATA ANALYSIS

11.3.1 Screening and enrolment logs

The QuinteT researcher will analyse data using the SEAR framework to observe differences between sites in recruitment patterns as new sites open (51). Simple descriptive analyses will identify points in the recruitment pathway at which patients are lost to recruitment to the cohort or trials and the reasons why. Detailed eligibility and recruitment pathways will be compiled for sites, noting the point at which patients receive information about the study, which members of the clinical team they meet, and the timing and frequency of appointments. Recruitment pathways will be compared with details specified in the trial protocol and pathways from other sites to identify practices that are potentially more/less efficient. Numbers of eligible and recruited patients will be compared across sites and considered in relation to estimates specified in the grant application/study protocol. These data will be triangulated with qualitative findings (see below) to identify barriers and potential solutions to recruitment.

11.3.2 Recordings of recruitment conversations and interviews

Audio recordings of recruitment conversations will be sought from a purposefully sampled range of recruiting sites (showing higher and lower recruitment) to ensure maximum variation and recordings will be analysed by the QuinteT researcher. The audio recordings will be used to explore information provision, management of patient treatment preferences, and randomisation decisions to identify recruitment difficulties and improve information provision. Audio-recorded recruitment consultations will be subjected to targeted transcription with relevant sections first identified then transcribed and identifying data removed before fuller analysis. Analysis will employ content, thematic, and novel analytical approaches, including targeted conversation analysis (52) and quanti-qual appointment timing (the ‘Q-Qat method’) (53), as described in the QuinteT recruitment intervention protocol [24]. Interview data will be analysed thematically using constant comparative approaches derived from Grounded Theory methodology (54).

Findings from the investigation of recruitment to the CARE trials will be fed back to the CI, TMG, and collaborator Bauld, where appropriate, to determine a plan of actions to optimise recruitment to the pilot trials. Actions may include feedback to individuals or in groups as appropriate and will include template patient pathways, individualised or generic ‘tips’ sheets for recruiters and delivery of recruiter training. Group feedback and training will be timed to coincide with the meetings of professional associations mentioned above.

12 HEALTH ECONOMICS AND DATA ANALYSIS

We will collect self-reported health service use and social/economic outcomes using bespoke question sets that will inform future economic analyses (9; 10). If data collection is confirmed as feasible, then a previously developed decision model (20) will be updated and further developed to incorporate data collected within this study to provide a putative estimate of cost-effectiveness and its drivers. In the context of the CARE pilot trial, the health economics objectives are to: (i) design and test an optimal mechanism for the capture of resource use and cost data in community NHS

settings, NHS secondary care, participants' out of pocket expenses and carer costs, (ii) estimate expected effect size and variance of relevant outcomes including health-related utility and quality-adjusted life years, and (iii) identify and measure the potential cost implications of surgical management of cavernomas. We will measure health-related utility (55), healthcare-related resource use and costs using participant questionnaires before randomisation and at each follow-up timepoint (56). These costs will be ratified by the study team through scrutiny of the patient pathway in both arms of the trials using available medical records to populate CRFs. We will assign unit costs using standard national costing sources where available, or through consultation with relevant service business managers. Costs will be summarised from the perspectives of (a) the NHS and personal social services, and (b) wider society (including participants' and their carers' out-of-pocket costs and lost productivity).

13 ADVERSE EVENTS

The PI is responsible for the detection and documentation of events meeting the criteria and definitions detailed below. This task may also be carried out by another suitably qualified clinician in the research team at that site who has been delegated this role. Only clinical outcomes and relevant serious adverse events (SAE) related to medical and surgical management that occur after randomisation until the final 6-month follow-up review must be recorded in the eCRF. Participants will be instructed to contact their local research team if any symptoms develop at any time after being randomised.

13.1 DEFINITIONS

An **adverse event** (AE) is any untoward medical occurrence in a clinical trial participant which does not necessarily have a causal relationship with an investigational medicinal product (IMP).

An **adverse reaction** (AR) is any untoward and unintended response to an IMP which is related to any dose administered to that participant.

A **serious adverse event** (SAE), **serious adverse reaction** (SAR). Any AE or AR that at any dose:

- results in death of the clinical trial participant;
- is life threatening*;
- requires in-patient hospitalisation^ or prolongation of existing hospitalisation;
- results in persistent or significant disability or incapacity;
- consists of a congenital anomaly or birth defect;
- results in any other significant medical event not meeting the criteria above.

*Life-threatening in the definition of an SAE or SAR refers to an event where the participant was at risk of death at the time of the event. It does not refer to an event which hypothetically might have caused death if it were more severe.

^Any hospitalisation that was planned prior to enrolment will not meet SAE criteria. Any hospitalisation that is planned post enrolment will meet the SAE criteria.

13.2 IDENTIFYING SAEs

Participants will be asked about the occurrence of SAEs wherever contact is made with them between randomisation and the final central six monthly follow up review. Open-ended and non-leading verbal questioning of the participant will be used to enquire about SAE occurrence. Only events which are clinical outcomes on the trial or are related to medical and surgical management will be recorded as AEs and SAEs. Participants will also be asked if they have been admitted to hospital, used any new medicines or changed concomitant medication regimens. If there is any doubt as to whether a clinical observation is an SAE, the event will be recorded. SAEs might also be identified via information from support departments e.g. laboratories.

13.3 RECORDING SAEs

When an SAE occurs, it is the responsibility of the PI, or another suitably qualified clinician in the study team who is delegated to record and report SAEs, to review all documentation (e.g. hospital notes, laboratory and diagnostic reports) related to the event. It is the PIs responsibility, or another suitably qualified clinician that has been delegated this role, to assess whether an AE is an outcome in the trial. The PI or delegated research team member will then record all relevant information in the CRF/AE log and on the SAE form (if the AE meets the criteria of serious). If the AE is detected by central means of follow-up, the TCC will initiate the collection of this information but enlist the help of local site research staff to acquire the relevant clinical and imaging information. Information to be collected includes type of event, onset date, clinical assessment of severity and causality, date of resolution as well as treatment required, investigations needed and outcome.

13.3.1 Pre-existing medical conditions

Pre-existing medical conditions (i.e. existed prior to informed consent) should be recorded as medical history and only recorded as SAEs if medically judged to have worsened during the trial and meet the definition of an SAE.

13.3.2 Worsening of the underlying condition during the trial

Medical occurrences or symptoms of deterioration that are expected to be due to the participant's underlying condition should be recorded in the participant's medical notes and only be recorded as SAEs if medically judged to have unexpectedly worsened during the trial. Events that are consistent with the expected progression of the underlying disease should not be recorded as SAEs.

13.4 ASSESSMENT OF AEs AND SAEs

Each AE which may be a clinical outcome for the trial or may be related to surgical management must be assessed for seriousness, causality, severity and ARs must be assessed for expectedness by the PI or another suitably qualified clinician in the study team who has been delegated this role.

The CI may not downgrade an event that has been assessed by an Investigator as an SAE or a related and unexpected SAE, but can upgrade an AE to an SAE, SAR or SUSAR if appropriate.

13.4.1 Assessment of Seriousness

The Investigator will make an assessment of seriousness as defined in Section 13.1.

13.4.2 Assessment of Causality

The Investigator will make an assessment of whether the AE/SAE is likely to be related to the study intervention according to the definitions below.

Unrelated: where an event is not considered to be related to the treatment allocated at randomisation.

Possibly Related: The nature of the event, the underlying medical condition, concomitant medication or temporal relationship make it possible that the AE has a causal relationship to the treatment allocated at randomisation.

13.4.3 Assessment of Expectedness

If the AE is judged to be related to the study interventions, the Investigator will make an assessment of expectedness.

Expected: The type of event is expected in line with the treatment allocated at randomisation.

Unexpected: The type of event was not listed in the protocol or is not an expected clinical occurrence.

13.4.4 Assessment of Severity

The Investigator will make an assessment of severity for each AE/SAE and record this on the CRF or SAE form according to one of the following categories:

Mild: an event that is easily tolerated by the participant, causing minimal discomfort and not interfering with every day activities.

Moderate: an event that is sufficiently discomforting to interfere with normal everyday activities.

Severe: an event that prevents normal everyday activities.

Note: the term ‘severe’, used to describe the intensity, should not be confused with ‘serious’ which is a regulatory definition based on participant/event outcome or action criteria. For example, a headache may be severe but not serious, while a minor stroke is serious but may not be severe.

13.5 REPORTING OF SAEs

Once the Investigator becomes aware that an SAE has occurred in a study participant, the information will be reported to the ACCORD (Academic and Clinical Central office for Research and Development) Research Governance & Quality Assurance (QA) Office **immediately or within 24 hours**. If the Investigator does not have all information regarding an SAE, they should not wait for this additional information before notifying ACCORD. The SAE report form can be updated when the additional information is received.

The SAE form will be emailed to ACCORD via Safety@accord.scot. Only forms in a PDF format will be accepted by ACCORD via email.

The Investigator will follow up each event until resolution. Where missing information has not been sent to ACCORD after an initial report, ACCORD will contact the investigator and request the missing information.

All reports faxed to ACCORD and any follow up information will be retained by the Investigator in the Investigator Site File (ISF).

The sponsor is responsible for reporting SAEs that are considered to be “possibly related” to the treatment allocation and “unexpected”, to the REC within 15 days of becoming aware of the event.

The TCC will provide SAE line listings from ACCORD for circulation prior to DMC meetings.

14 PREGNANCY

Although pregnancy is not considered an AE or SAE; as a matter of safety, the Investigator will be required to record any female participant’s pregnancy which occurs while participating in the study. The Investigator will need to record the information on a Pregnancy Notification Form and submit this to the ACCORD office within 14 days of being made aware of the pregnancy. All pregnant female participants will be followed up until the outcome of the pregnancy.

15 OVERSIGHT ARRANGEMENTS

15.1 TRIAL MANAGEMENT GROUP

The trial will be coordinated by a TMG, consisting of the CI, grant holders, Trial Manager and PAG members. The roles and responsibilities of the TMG and the names of committee members are detailed in the TMG charter.

The Trial Manager will coordinate and oversee the trial and will be accountable to the CI. The Data Manager will be responsible for checking the CRFs for completeness, plausibility and consistency. Any queries will be resolved by the Investigator or delegated member of the site team.

15.2 TRIAL STEERING COMMITTEE

A Trial Steering Committee (TSC) will be established to oversee the conduct and progress of the trial. The terms of reference of the TSC, reporting arrangements and the names of committee members are detailed in the TSC charter.

15.3 DATA MONITORING COMMITTEE

An independent Data Monitoring Committee (DMC) will be established to oversee the safety of participants in the trial. The terms of reference of the Data Monitoring Committee and the names of committee members are detailed in the DMC charter. The DMC Charter will be signed by the appropriate individuals before recruitment to the trial starts.

15.4 PATIENT ADVISORY GROUP

The patient advocacy organisation CAUK will organise input from a diverse Patient Advisory Group which will aim to meet bi-monthly. Two representatives of this PAG will join the TSC. The terms of reference of the Patient Advisory Group and the names of committee members are detailed in the PAG Terms of Reference.

15.5 INSPECTION OF RECORDS

Investigators and institutions involved in the study will permit trial related monitoring and audits on behalf of the sponsor, REC review, and regulatory inspection(s). In the event of audit or monitoring, the Investigator agrees to allow the representatives of the sponsor direct access to all study records and source documentation. In the event of regulatory inspection, the Investigator agrees to allow inspectors direct access to all study records and source documentation.

15.6 STUDY MONITORING AND AUDIT

The ACCORD Sponsor Representative will assess the study to determine if an independent risk assessment is required. If required, the independent risk assessment will be carried out by the ACCORD Quality Assurance Group to determine if an audit should be performed before/during/after the study and, if so, at what frequency.

Risk assessment, if required, will determine if audit by the ACCORD QA group is required. Should audit be required, details will be captured in an audit plan. Audit of Investigator sites, study management activities and study collaborative units, facilities and 3rd parties may be performed.

16 GOOD CLINICAL PRACTICE

16.1 ETHICAL CONDUCT

The study will be conducted in accordance with the principles of the International Conference on Harmonisation Tripartite Guideline for Good Clinical Practice (ICH GCP). Before the study can commence, all required approvals will be obtained and any conditions of approvals will be met.

16.2 INVESTIGATOR RESPONSIBILITIES

The PI is responsible for the overall conduct of the study at the site and compliance with the protocol and any protocol amendments. In accordance with the principles of ICH GCP, the following areas listed in this section are also the responsibility of the PI. Responsibilities may be delegated to an appropriate member of study site staff. A Delegation Log will be prepared for each site, detailing the responsibilities of each member of staff working on the trial.

16.2.1 Informed Consent

The PI is responsible for ensuring informed consent is obtained before any protocol specific procedures are carried out. The decision of a participant to participate in clinical research is voluntary and should be based on a clear understanding of what is involved.

Participants must receive adequate oral and written information – appropriate PILs and ICFs will be provided. The oral explanation to the participant will be performed by the PI or qualified delegated person, and must cover all the elements specified in the PIL and ICF. The participant must be given every opportunity to clarify any points they do not understand and, if necessary, ask for more information. The participant must be given sufficient time to consider the information provided. It should be emphasised that the participant may withdraw their consent to participate at any time without loss of benefits to which they otherwise would be entitled. The participant will be informed and agree to their medical records being inspected by regulatory authorities and representatives of the sponsor(s).
The PI or delegated member of the research team and the participant will sign and date the ICF(s) to confirm that consent has been obtained. The participant will receive a copy of this document and a copy filed in the Investigator Site File (ISF) and participant's medical notes (if applicable).

16.2.2 Study Site Staff

The PI and research team must be familiar with the protocol and the study requirements. It is the PIs responsibility to ensure that all staff assisting with the study are adequately informed about the protocol and their trial related duties.

16.2.3 Data Recording

The PI is responsible for the quality of the data recorded in the CRF at each Investigator Site.

16.2.4 Investigator Documentation

The PI will ensure that the required documentation is available in local Investigator Site files.

16.2.5 Training

16.2.5.1 Recruitment site training

Research teams will be trained on the trial protocol, sponsor SOPs and QRI processes by the trial team and qualitative researcher (in person or remotely). This will be completed before the site is permitted to open to recruitment.

QRI training of PIs and recruiters will take place as needed and as indicated by QRI findings as described in 3.1.1.2 above. Findings from data collected during the QRI will be presented to the CI and TMG and a plan of action formulated to improve recruitment and information provision. Generic challenges such as how to explain study processes (e.g. randomisation) may be addressed through dissemination of 'tips and guidance' documents. Supportive feedback will be a core component of the plan of action, with the exact nature and timing dependent on the issues that arise. Site-specific feedback may cover institutional barriers, while multi-centre group feedback sessions may address widespread challenges, that would benefit from discussion. All group feedback sessions will be aided by de-identified data extracts from interviews and recorded recruitment conversations. Individual confidential feedback will also be offered, particularly where recruiters experience specific difficulties or where there is a need to discuss potentially sensitive issues. Investigator meetings and site visits may also be employed to discuss technical or clinical challenges (e.g. discomfort surrounding eligibility criteria).

16.2.5.2 GCP training

For non-CTIMP (i.e. non-drug) studies all researchers are encouraged to undertake Good Clinical Practice (GCP) training in order to understand the principles of GCP. However, this is not a mandatory requirement unless deemed so by the sponsor. GCP training status for all research team members should be indicated in their respective CVs or a GCP certificate may be provided.

16.2.6 Confidentiality

All laboratory specimens, evaluation forms, reports, and other records must be identified in a manner designed to maintain participant confidentiality. All records must be kept in a secure storage area with limited access. The PI and research site staff involved with this study may not disclose or use for any purpose other than performance of the study, any data, record, or other unpublished information, which is confidential or identifiable, and has been disclosed to those individuals for the purpose of the study. Prior written agreement from the sponsor or its designee must be obtained for the disclosure of any said confidential information to parties not involved in the trial.

16.2.7 Data Protection

All PIs and research team staff (including central research team staff and qualitative research staff) involved with this study must comply with the requirements of the appropriate data protection legislation (including the General Data Protection Regulation and Data Protection Act) with regard to the collection, storage, processing and disclosure of personal information.

Computers used to collate the data will have limited access measures via user names and passwords.

Published results will not contain any personal data and be of a form where individuals are not identified and re-identification is not likely to take place.

STUDY CONDUCT RESPONSIBILITIES

16.3 PROTOCOL AMENDMENTS

Any changes in research activity, except those necessary to remove an apparent, immediate hazard to the participant in the case of an urgent safety measure, must be reviewed and approved by the CI.

Amendments will be submitted to a sponsor representative for review and authorisation before being submitted in writing to the appropriate REC, and local R&D for approval prior to participants being enrolled into an amended protocol.

16.4 MANAGEMENT OF PROTOCOL NON-COMPLIANCE

Prospective protocol deviations, i.e. protocol waivers, will not be approved by the sponsors and therefore will not be implemented, except where necessary to eliminate an immediate hazard to study participants. If this necessitates a subsequent protocol amendment, this should be submitted to the REC, and local R&D for review and approval if appropriate.

Protocol deviations will be recorded in a protocol deviation log and logs will be submitted to the sponsors every 3 months. Each protocol violation will be reported to the sponsor within 3 days of becoming aware of the violation. All protocol deviation logs and violation forms should be emailed to QA@accord.scot

Deviations and violations are non-compliance events discovered after the event has occurred. Deviation logs will be maintained for each site in multi-centre studies. An alternative frequency of deviation log submission to the sponsors may be agreed in writing with the sponsors.

The following will not be recorded as protocol deviations:

- Missed audio-recordings of conversations by research teams.
- Lack of adherence to the randomised treatment allocation.

16.5 SERIOUS BREACH REQUIREMENTS

A serious breach is a breach which is likely to effect to a significant degree: (a) the safety or physical or mental integrity of the participants of the trial; or(b) the scientific value of the trial.

If a potential serious breach is identified by the CI, a site PI or delegates, the co-sponsors must be notified via seriousbreach@accord.scot within 24 hours. It is the

responsibility of the co-sponsors to assess the impact of the breach on the scientific value of the trial, to determine whether the incident constitutes a serious breach and report to REC as necessary.

16.6 STUDY RECORD RETENTION

All trial documentation will be kept for a minimum of three years from the protocol defined end of trial point. When the minimum retention period has elapsed, trial documentation will not be destroyed without permission from the sponsor.

QRI audio-recordings will be kept for at least 10 years before they will be destroyed and electronic transcripts will be stored indefinitely in secure research data storage.

16.7 END OF TRIAL

The end of study is defined as the last participant's last visit. This will be a 6-month follow up review.

The PIs or the co-sponsor(s) have the right at any time to terminate the study for clinical or administrative reasons.

The end of the study will be reported to the REC, and Research and Development Offices and co-sponsors within 90 days, or 15 days if the study is terminated prematurely. The PIs will inform participants if the study is closed prematurely and ensure that the appropriate follow up is arranged for all participants involved.

End of study notification will be reported to the co-sponsors via email to resgov@accord.scot.

16.8 CONTINUATION OF TREATMENT FOLLOWING THE END OF STUDY

There are no provisions for ancillary or care for participants after the trial ends, because the interventions in the CARE pilot trial are provided in standard clinical practice and aftercare will occur as normal in standard practice.

16.9 INSURANCE AND INDEMNITY

The co-sponsors are responsible for ensuring proper provision has been made for insurance or indemnity to cover their liability and the liability of the CI and staff. The following arrangements are in place to fulfil the co-sponsors' responsibilities:

- The protocol has been designed by the CI, researchers employed by the University and the TMG. The University has insurance in place (which includes no-fault compensation) for negligent harm caused by poor protocol design by the CI and researchers employed by the University.
- Sites participating in the study will be liable for clinical negligence and other negligent harm to individuals taking part in the study and covered by the duty of care owed to them by the sites concerned. The co-sponsors require individual sites participating in the study to arrange for their own insurance or indemnity in respect of these liabilities.
- Sites which are part of the United Kingdom's National Health Service will have the benefit of NHS Indemnity.

- Sites outside the United Kingdom may be responsible for arranging their own indemnity or insurance for their participation in the study, and will be responsible for compliance with local law applicable to their participation in the study.

17 REPORTING, PUBLICATIONS AND NOTIFICATION OF RESULTS

17.1 AUTHORSHIP POLICY AND REPORTING

On completion of the study, the study data will be analysed and tabulated, and a clinical study report will be prepared in accordance with the International Conference on Harmonisation guidelines.

A final research report will be prepared as required by the funder. A summary report of the study will be provided to the REC within one year of the end of the study.

The success of the CARE pilot trial will be determined by the collaboration of a large number of doctors, nurses, other health professionals, patients, relatives, and the patient support organisation CAUK. For this reason, the credit for the main results will be given, not exclusively to the TMG, but to all collaborators with the trial. The primary trial publication will be drafted by a writing committee drawn from the TMG, whose membership has been approved by the TSC. Authorship will be under a group name for the CARE pilot trial collaboration and include the writing committee. People included on active sites' delegation logs will be included in any listing of collaborators in trial publications. The manuscript will be approved by the TSC before submission for publication.

17.2 PUBLICATION AND DISSEMINATION

Publications will be managed in line with funder requirements. We will submit manuscripts to peer reviewed journals, describing the findings of the QuinteT recruitment intervention and the CARE pilot trial (in addition to the final report for publication in the HTA journal). We will pay for these papers to be published open access. We will also present our findings at meetings of the Association of British Neurologists, the Society of British Neurological Surgeons, the British Paediatric Neurosurgery Society, and the British Paediatric Neurology Association.

We will disseminate a plain English summary of the findings of the CARE pilot trial to participants and public audiences with input from, and acknowledgement of, the Patient Advisory Group. We will offer to present our project and its findings to the annual meetings of CAUK, which is a national event that gives people affected by cavernoma a voice to talk about the issues that matter to them. We will produce an easy access report of our findings to share with the public and patients, and we will post it in the public domain on the CAUK website. We will keep the public, patients, and carers informed about study progress and results via social media channels (Facebook and Twitter).

17.3 DATA SHARING

Ownership of the data arising from this study resides with the study team.

Following publication of the primary paper, a de-identified individual participant data set will be prepared for sharing purposes. All data requests should be submitted to the CI for consideration. Access to de-identified data may be granted following review by CI and TMG.

Data collected during PAG discussions or in QuinteT recruitment intervention data collection with patients may include quotes that will be useful to CAUK in producing or optimising existing patient or carer information; where participant consent has been given, these data (after removing or disguising identifiers) will be made available by the QuinteT research group in Bristol to CAUK in order to maximise their impact.

At the end of the study, QRI audio-recordings will be kept for at least 10 years before they will be destroyed. Transcripts will be stored indefinitely in secure research data storage, which can be accessed by approved individuals who are interested in conducting their own analyses of the data. These individuals will have to submit an application to do this, which will be assessed by an independent committee. However, all data will have identifiable information removed before they are made available, and there will be no way to identify individuals mentioned in interviews/appointments.

18 TRIAL TIMELINE

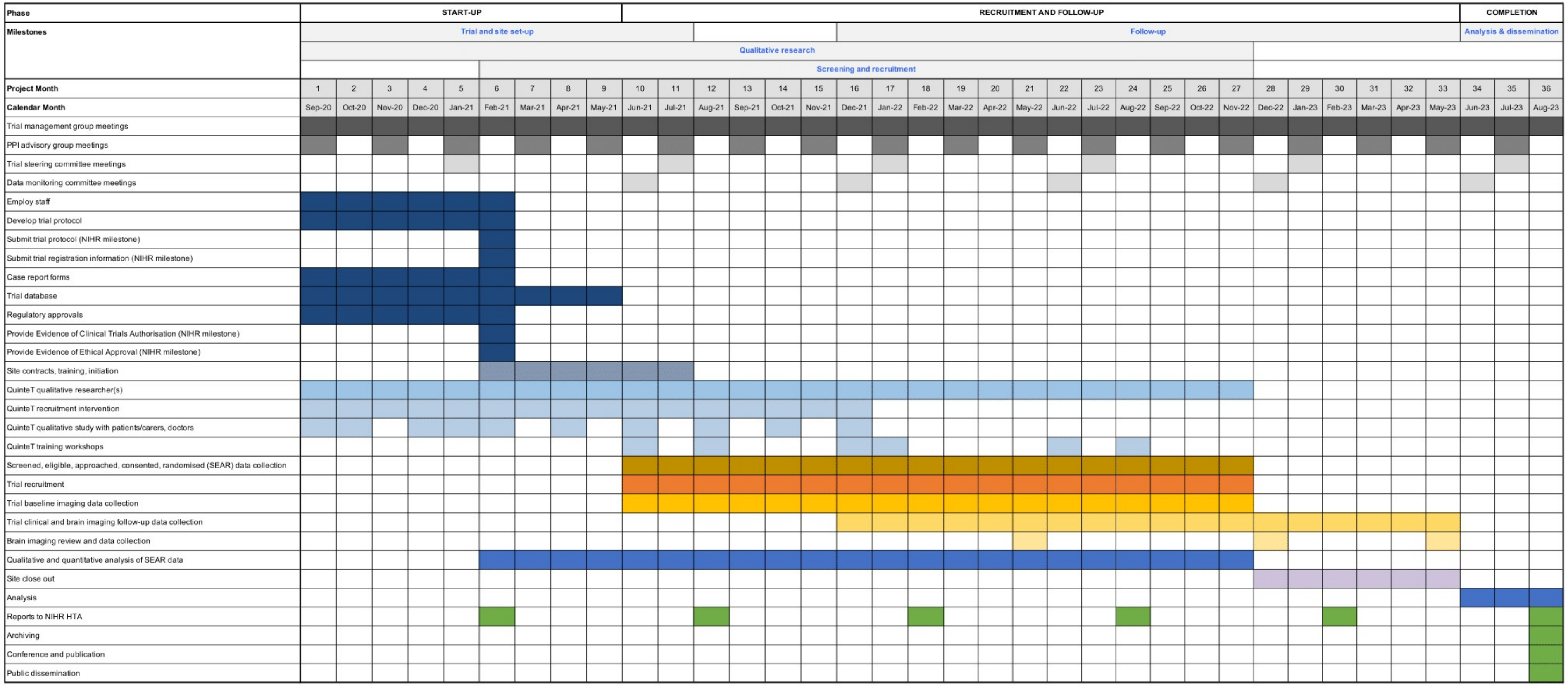

Footnote: Trial delivery timings are targets, variations will not be recorded as a protocol deviation/violation.

19 PROTOCOL VERSION CONTROL HISTORY

19.1 Version 1.0 (29Jan2021)

Original sponsor-approved version, submitted as part of application for REC review.

19.2 Version 2.0 (22Mar2021)

Protocol updated following REC meeting comments. Summary of changes:

- REC reference added to cover page table (page 1).
- Specific reference to Gamma Knife stereotactic radiosurgery added throughout and clarification added that neurosurgery and Gamma Knife stereotactic radiosurgery will be used according to their availability in clinical practice (section 3, 7 and throughout).
- Clarification added that imaging studies performed because of the occurrence of an outcome event will be collected by the research team and uploaded to the scan database for the trial (section 8.1.6)
- Trial timeline added (section 18).
- Version history table added (section 19).

20 REFERENCES

1. **Labauge P, Denier C, Bergametti F, Tournier-Lasserve E.** Genetics of cavernous angiomas. *Lancet Neurol.* 2007, Vol. 6, 3, pp. 237-244.
2. **Morris Z, Whiteley WN, Longstreth WT, Jr., Weber F, Lee YC, Tsushima Y, et al.** Incidental findings on brain magnetic resonance imaging: systematic review and meta-analysis. *BMJ.* 2009, Vol. 339, b3016.
3. **Al-Shahi Salman R, Berg MJ, Morrison L, Awad IA, Angioma Alliance Scientific Advisory Board.** Hemorrhage from cavernous malformations of the brain: definition and reporting standards. *Stroke.* 2008, Vol. 39, 12, pp. 3222-30.
4. **Al-Shahi R, Bhattacharya JJ, Currie DG, Papanastassiou V, Ritchie V, Roberts RC, et al.** Prospective, population-based detection of intracranial vascular malformations in adults: the Scottish Intracranial Vascular Malformation Study (SIVMS). *Stroke.* 2003, Vol. 34, 5, pp. 1163-1169.
5. **Horne MA, Flemming KD, Su IC, Stapf C, Jeon JP, Li D, et al.** Clinical course of untreated cerebral cavernous malformations: a meta-analysis of individual patient data. *Lancet Neurol.* 2016, Vol. 15, 2, pp. 166-173.
6. **Josephson CB, Leach JP, Duncan R, Roberts RC, Counsell CE, Al-Shahi Salman R, et al.** Seizure risk from cavernous or arteriovenous malformations: prospective population-based study. *Neurology.* 2011, Vol. 76, 8, pp. 1548-1554.
7. **Miller CE, Quayyum Z, McNamee P, Al-Shahi Salman R, SIVMS Steering Committee.** Economic burden of intracranial vascular malformations in adults: prospective population-based study. *Stroke.* 2009, Vol. 40, 6, pp. 1973-1979.
8. **Samarasekera N, Poorthuis M, Kontoh K, Stuart I, Respinger C, Berg J, et al.** Guidelines for the management of cerebral cavernous malformations in adults. . *Genetic Alliance UK & Cavernoma Alliance UK.* 2012.
9. **Moultrie F, Horne MA, Josephson CB, Hall JM, Counsell CE, Bhattacharya JJ, et al.** Outcome after surgical or conservative management of cerebral cavernous malformations. *Neurology.* 2014, Vol. 83, 7, pp. 582-589.
10. **Bicalho VC, Bergmann A, Domingues F, Frossard JT, de Souza J.** Cerebral Cavernous Malformations: Patient-Reported Outcome Validates Conservative Management. *Cerebrovasc Dis.* 2017, Vol. 44, 5-6, pp. 313-319.
11. **Polster SP, Cao Y, Carroll T, Flemming K, Girard R, Hanley D, et al.** Trial Readiness in Cavernous Angiomas With Symptomatic Hemorrhage (CASH). *Neurosurgery.* 2019, Vol. 84, 4, pp. 954-964.
12. **Qiao N, Ma Z, Song J, Wang Y, Shou X, Zhang X, et al.** A systematic review and meta- analysis of surgeries performed for treating deep-seated cerebral cavernous malformations. 2015, Vol. 29, 4, pp. 493-499.
13. **Poorthuis M, RInkel LA, Lammy S, Al-Shahi Salman R.** Stereotactic radiosurgery for cerebral cavernous malformations: a systematic review and meta-analysis. *Neurology.* 2019, Vol. (in press).
14. **Poorthuis M, Samarasekera N, Kontoh K, Stuart I, Cope B, Kitchen N, et al.** Comparative studies of the diagnosis and treatment of cerebral cavernous malformations in adults: systematic review. . *Acta Neurochir (Wien) .* 2013, Vol. 155, 4, pp. 643-649.
15. **Akers A, Al-Shahi Salman R, I AA, Dahlem K, Flemming K, Hart B, et al.** Synopsis of Guidelines for the Clinical Management of Cerebral Cavernous Malformations: Consensus Recommendations Based on Systematic Literature Review by the Angioma Alliance Scientific Advisory Board Clinical Experts Panel. *Neurosurgery.* 2017, Vol. 80, 5, pp. 665-680.
16. **Rooshenas L, Paramasivan S, Jepson M, Donovan JL.** Intensive Triangulation of Qualitative Research and Quantitative Data to Improve Recruitment to Randomized Trials: The QuinteT Approach. *Qual Health Res.* 2019, Vol. 29, 5, pp. 672-679.

17. **Glasziou P, Chalmers I, Rawlins M, McCulloch P.** When are randomised trials unnecessary? Picking signal from noise. *BMJ.* 2007, Vol. 334, 7589, pp. 349-351.
18. **Poorthuis MH, Klijn CJ, Algra A, Rinkel GJ, Al-Shahi Salman R.** Treatment of cerebral cavernous malformations: a systematic review and meta-regression analysis. *J Neurol Neurosurg Psychiatry.* 2014, Vol. 85, 12, pp. 1319-1323.
19. **Rinkel LA, Al-Shahi Salman R, Rinkel GJ, Greving JP.** Radiosurgical, neurosurgical, or no intervention for cerebral cavernous malformations: A decision analysis. *Int J Stroke.* 2019, Vol. (in press).
20. **Esposito P, Coulbois S, Kehrli P, Boyer P, Dietemann JL, Rousseaux P, et al.** Place of the surgery in the management of brainstem cavernomas. Results of a multicentric study]. *Neurochirurgie.* Vol. 49, 1, pp. 5-12.
21. **Mathiesen T, Edner G, Kihlstrom L.** Deep and brainstem cavernomas: a consecutive 8-year series. *J Neurosurg.* 2003, Vol. 99, 1, pp. 31-37.
22. **Tarnaris A, Fernandes RP, Kitchen ND.** Does conservative management for brain stem cavernomas have better long-term outcome? *Br J Neurosurg.* 2008, Vol. 22, 6, pp. 748-757.
23. **Huang AP, Chen JS, Yang CC, Wang KC, Yang SH, Lai DM, et al.** Brain stem cavernous malformations. *J Clin Neurosci.* 2010, Vol. 17, 1, pp. 74-79.
24. **Kivelev J, Niemela M, Kivisaari R, Dashti R, Laakso A, Hernesniemi J.** Long-term outcome of patients with multiple cerebral cavernous malformations. *Neurosurgery.* 2009, Vol. 65, 3, pp. 450-455.
25. **Kida Y, Hasegawa T, Kato T, Sato T, Nagai H, Hishikawa T, et al.** Natural History of Symptomatic Cavernous Malformations and Results of Surgery. *Jpn J Neurosurg (Tokyo).* 2015, Vol. 24, pp. 108-118.
26. **Yoon PH, Kim DI, Jeon P, Ryu YH, Hwang GJ, Park SJ.** Cerebral cavernous malformations: serial magnetic resonance imaging findings in patients with and without gamma knife surgery. . *Neurol Med Chir (Tokyo) .* 1998, Vol. 38, Suppl, pp. 255-261.
27. **Lu XY, Sun H, Xu JG, Li QY.** Stereotactic radiosurgery of brainstem cavernous malformations: a systematic review and meta-analysis. *J Neurosurg.* 2014, Vol. 120, 4, pp. 982-987.
28. **Al-Shahi Salman R, Kitchen N, Thomson J, Ganesan V, Mallucci C, Radatz M, et al.** Top ten research priorities for brain and spine cavernous malformations. *Lancet Neurol .* 2016, Vol. 15, 4, pp. 354-355.
29. **Stereotactic, NHS England Clinical Reference Group for.** *Clinical Commissioning Policy: Stereotactic Radiosurgery / Radiotherapy for Cavernous Venous Malformations (Cavernomas).* s.l. : NHS England, 2013. p. D05/P/g.
30. **Raymond J, Darsaut TE, Molyneux AJ, Team collaborative Group.** A trial on unruptured intracranial aneurysms (the TEAM trial): results, lessons from a failure and the necessity for clinical care trials. *Trials.* 2015, Vol. 10, 8, p. e0136619.
31. **Magro E, Gentric JC, Darsaut TE, Ziegler D, Bojanowski MW, Raymond J.** Responses to ARUBA: a systematic review and critical analysis for the design of future arteriovenous malformation trials. *J Neurosurg.* 2 126 2017, Vols. 486-494.
32. **Donovan JL, Paramasivan S, de Salis I, Toerien M.** Clear obstacles and hidden challenges: understanding recruiter perspectives in six pragmatic randomised controlled trials. *Trials.* 2014, Vol. 15, 5.
33. **Beasant L, Brigden A, Parslow RM, Apperley H, Keep T, Northam A, et al.** Treatment preference and recruitment to pediatric RCTs: A systematic review. *Contemp Clin Trials Commun.* 2019, Vol. 14, 100335.
34. **Donovan JL, Rooshenas L, Jepson M, Elliott D, Wade J, Avery K, et al.** Optimising recruitment and informed consent in randomised controlled trials: the development and implementation of the Quintet Recruitment Intervention (QRI). *Trials.* 2016, Vol. 17, 1, p. 283.
35. **Donovan J, Mills N, Smith M, Brindle L, Jacoby A, Peters T, et al.** Quality improvement report: Improving design and conduct of randomised trials by embedding them in qualitative research: ProtecT (prostate testing for cancer and treatment) study. *BMJ.* 2002, Vol. 325, 7367, pp. 766-770.

36. **Mills N, Donovan JL, Wade J, Hamdy FC, Neal DE, Lane JA.** Exploring treatment preferences facilitated recruitment to randomized controlled trials. *J Clin Epidemiol.* 2011, Vol. 64, 10, pp. 1127-1136.
37. **Mills N, Gaunt D, Blazeby JM, Elliott D, Husbands S, Holding P, et al.** Training health professionals to recruit into challenging randomized controlled trials improved confidence: the development of the QuinteT randomized controlled trial recruitment training intervention. *J Clin Epidemiol.* 2018, Vol. 95, pp. 34-44.
38. **Paramasivan S, Huddart R, Hall E, Lewis R, Birtle A, Donovan JL.** Key issues in recruitment to randomised controlled trials with very different interventions: a qualitative investigation of recruitment to the SPARE trial (CRUK/07/011). T. *Trials.* 2011, Vol. 12, 78.
39. **Rooshenas L, Scott LJ, Blazeby JM, Rogers CA, Tilling KM, Husbands S, et al.** he QuinteT Recruitment Intervention supported five randomized trials to recruit to target: a mixed- methods evaluation. *J Clin Epidemiol.* 2019, Vol. 106, pp. 108-120.
40. **Mills N, Blazeby JM, Hamdy FC, Neal DE, Campbell B, Wilson C, et al.** Training recruiters to randomized trials to facilitate recruitment and informed consent by exploring patients' treatment preferences. *Trials.* 2014, Vol. 15, 323.
41. **Willie JT, Malcolm JG, Stern MA, Lowder LO, Neill SG, Cabaniss BT, et al.** Safety and effectiveness of stereotactic laser ablation for epileptogenic cerebral cavernous malformations. *Epilepsia.* 2019, Vol. 60, 2, pp. 220-232.
42. **Treweek S, Pitkethly M, Cook J, Fraser C, Mitchell E, Sullivan F, et al.** Strategies to improve recruitment to randomised trials. *Cochrane Database Syst Rev 2018.* 2018, Vol. 2, MR000013.
43. **Rigamonti D, Drayer D P, Johnson P C, Hadley N M, Zabramski J, Spetzler, R F.** The MRI appearance of cavernous malformations (angiomas). *J Neurosurg.* 1987, Vol. 67, 4, pp. 518-524.
44. **Rosenow F, Alonso-Vanegas M A, Baumgartner C, Blümcke I, Carreño M, Gizewksi, E R, Hamer, H M, Knake S, Kahane P, Lüders H O, Mathern G W, Menzler K, Miller J, Otsuki T, Ozkara C, Pitkänen A, Roper S N, Sakamoto A C, Sure U, Walker M C, Steinhoff B J, Sur.** Cavernoma-related epilepsy: review and recommendations for management--report of the Surgical Task Force of the ILAE Commission on Therapeutic Strategies. *Epilepsia.* 2013, Vol. 54, 12, pp. 2025-2035.
45. **Authority, Health Research.** Applying a proportionate approach to the process of seeking consent (V1.01). [Online] 17 January 2017. [Cited: 22 September 2020.] https://www.hra.nhs.uk/media/documents/applying-proportionate-approach-process-seeking-consent.pdf.
46. **Principles of consent: Children and Young People (England, Wales and Northern Ireland). *Health Research Authority.* [Online] [Cited: 21 September 2020.] http://www.hra-decisiontools.org.uk/consent/principles-children-EngWalesNI.html.**
47. Principles of consent: Children and Young People (Scotland). *Health Research Authority.* [Online] [Cited: 21 September 2020.] http://www.hra-decisiontools.org.uk/consent/principles-children-Scotland.html.
48. National Consent Advisory Group. *National Consent Policy V1.3 (June 2019).* s.l. : Health Service Executive, 2019.
49. Chohan MO, Marchio S, Morrison LA, Sidman RL, Cavenee WK, Dejana E, et al. Emerging Pharmacologic Targets in Cerebral Cavernous Malformation and Potential Strategies to Alter the Natural History of a Difficult Disease: A Review. *JAMA Neurol.* 2019, Vol. 76, 4, pp. 492-500.
50. The Mental Capacity Act 2005 (Loss of Capacity during Research Project) (England) Regulations 2007. *https://www.legislation.gov.uk/uksi/2007/679/schedule/2/made.* [Online] [Cited: 20 January 2020.]
51. S M Eldridge, C L Chan, M J Campbell, C M Bond, S Hopewell, LThabane, G A Lancaster on behalf of the PAFS consensus group. CONSORT 2010 statement: extension to randomised pilot and feasibility trials. *BMJ.* 2016, Vol. 355, i5239.

52. Herdman M, Gudex C, Lloyd A, Janssen M, Kind P, Parkin D, et al. Development and preliminary testing of the new five-level version of EQ-5D (EQ-5D-5L). *Qual Life Res.* 2011, Vol. 20, 10, pp. 1727-1736.
53. Wade, J, Donovan, JL, Lane, JA, Neal, DE, Hamdy, FC. It's not just what you say, it's also how you say it: opening the 'black box' of informed consent appointments in randomised controlled trials. *Social Sciene and Medicine (1982).* 2018-2028, 2009, Vol. 68, 11, pp. 2018-2028.
54. Paramasivan, S, Strong, S, Wilson, C, Campbell, B, Blazeby, JM, Donovan, JL. A simple techniqeu to identify key recruitment issues in randomised controlled trials. *Trials.* 2015, Vol. 16, 88.
55. Strauss, A. Corbin, J. *Grounded theory methodology. Handbook of qualitative research .* s.l. : Sage Publications Inc, 1994. pp. 273-85.
56. Royston P, Parmar MK. Restricted mean survival time: an alternative to the hazard ratio for the design and analysis of randomized trials with a time-to-event outcome. *BMC Med Res Methodol.* 2013, Vol. 13, 152.
57. Zanello M, Meyer B, Still M, Goodden JR, Colle H, Schichor C, et al. Surgical resection of cavernous angioma located within eloquent brain areas: International survey of the practical management among 19 specialized centers. *Seizure.* 2019, Vol. 69, pp. 31-40.
58. Wilson C, Rooshenas L, Paramasivan S, Elliott D, Jepson M, Strong S, et al. Development of a framework to improve the process of recruitment to randomised controlled trials (RCTs): the SEAR (Screened, Eligible, Approached, Randomised) framework. *Trials.* 2018, Vol. 19, 1, p. 50.
59. Rooshenas L, Elliott D, Wade J, Jepson M, Paramasivan S, Strong S, et al. Conveying Equipoise during Recruitment for Clinical Trials: Qualitative Synthesis of Clinicians' Practices across Six Randomised Controlled Trials. *PLoS Med .* :e1002147, 2016, Vol. 13, 10, p. e1002147.
